# Supplementary material for: Putative Identification of New Phragmaline-Type Limonoids from the Leaves of Swietenia macrophylla King: A Case Study Using Mass Spectrometry-Based Molecular Networking
Source: Molecules. 2023 Nov 15;28(22):7603. doi: 10.3390/molecules28227603 (PMC10673509; doi:10.3390/molecules28227603)
Supplement: Supplementary file 1 [file molecules-28-07603-s001.zip › molecules-2599863-supplementary.pdf]

molecules

Supplementary Information

for

Putative Identification of New Phragmaline-Type Limonoids from the Leaves of *Swietenia macrophylla* King: A Case Study Using Mass Spectrometry-Based Molecular Networking

José Diogo E. Reis [1,2,†], Paulo Wender P. Gomes [3,4,†,*], Paulo R. da C. Sá [5], Sônia das G. S. R. Pamplona [1], Consuelo Yumiko Y. e Silva [1,6], Maria Fátima das G. F. da Silva [7], Anupam Bishayee [8] and Milton Nascimento da Silva [1,2,6,*]

1 Laboratory of Liquid Chromatography, Institute of Exact and Natural Sciences, Federal University of Pará, Belém 66075-110, Brazil; reisdiogo190@gmail.com (J.D.E.R.); sgsrp@ufpa.br (S.d.G.S.R.P.); yumikoyoshioka@yahoo.com.br (C.Y.Y.e.S.)
2 Chemistry Post-Graduation Program, Institute of Exact and Natural Sciences, Federal University of Pará, Belém 66075-110, Brazil
3 Collaborative Mass Spectrometry Innovation Center, Skaggs School of Pharmacy and Pharmaceutical Sciences, University of California San Diego, La Jolla, CA 92093, USA
4 Skaggs School of Pharmacy and Pharmaceutical Sciences, University of California San Diego, La Jolla, CA 92093, USA
5 Federal Institute of Pará, Campus Castanhal, Castanhal 68740-970, Brazil; paulo.sa@ifpa.edu.br
6 Pharmaceutical Science Post-Graduation Program, Institute of Health Sciences, Federal University of Pará, Belém 66075-110, Brazil
7 Department of Chemistry, Federal University of São Carlos, São Carlos 13565-905, Brazil; dmfs@ufscar.br
8 College of Osteopathic Medicine, Lake Erie College of Osteopathic Medicine, Bradenton, FL 16509, USA; abishayee@lecom.edu or abishayee@gmail.com
* Correspondence: wendergomes@health.ucsd.edu (P.W.P.G.); yumilton@yahoo.com.br (M.N.d.S.)
† These authors contributed equally to this work.

List of Supporting Information

Table S1. HRMS and MS/MS data of the metabolites in *S. macrophylla* leaves extract detected by UPLC–MS/MS.
Table S2. *In-house* database containing 270 combinations of putative phragmalin-type limonoids.
Table S3. General fragmentation reactions observed in phragmalin-limonoid type.
Figure S1. Molecular network from *Swietenia macrophylla* G. King dichloromethane extract (DCMEt) using UPLC-HRMS in positive ionization mode ESI$^+$ (https://gnps.ucsd.edu/ProteoSAFe/status.jsp?task=8b7d836ad2694de0b932cf65db8c3fae). The network consist of 218 molecular features and the dataset be available in MassIVE CCMS MSV000087547 (https://massive.ucsd.edu/ProteoSAFe/dataset.jsp?task=f6307135be7c416584e8327d36c07146).
Figure S2. Fragmentation pattern of limonoid **1** computed from tandem MS data.
Figure S3. Fragmentation pattern of limonoid **2** computed from tandem MS data.
Figure S4. Fragmentation pattern of limonoid **3** computed from tandem MS data.
Figure S5. Fragmentation pattern of limonoid **4** computed from tandem MS data.
Figure S6. Fragmentation pattern of limonoid **5** computed from tandem MS data.
Figure S7. Fragmentation pattern of limonoid **6** computed from tandem MS data.
Figure S8. Fragmentation pattern of limonoid **7** computed from tandem MS data.
Figure S9. Fragmentation pattern of limonoid **8** computed from tandem MS data.
Figure S10. Fragmentation pattern of limonoid **9** computed from tandem MS data.
Figure S11. Fragmentation pattern of limonoid **10** computed from tandem MS data.
Figure S12. Fragmentation pattern of limonoid **11** computed from tandem MS data.
Figure S13. Fragmentation pattern of limonoid **12** computed from tandem MS data.
Figure S14. Fragmentation pattern of limonoid **13** computed from tandem MS data.
Figure S15. Fragmentation pattern of limonoid **14** computed from tandem MS data.
Figure S16. Fragmentation pattern of limonoid **15** computed from tandem MS data.
Figure S17. Fragmentation pattern of limonoid **16** computed from tandem MS data.
Figure S18. Fragmentation pattern of limonoid **17** computed from tandem MS data.
Figure S19. Fragmentation pattern of limonoid **18** computed from tandem MS data.
Figure S20. Fragmentation pattern of limonoid **19** computed from tandem MS data.
Figure S21. Fragmentation pattern of limonoid **20** computed from tandem MS data.
Figure S22. Fragmentation pattern of limonoid **21** computed from tandem MS data.
Figure S23. Fragmentation pattern of limonoid **22** computed from tandem MS data.
Figure S24. Fragmentation pattern of limonoid **23** computed from tandem MS data.
Figure S25. Fragmentation pattern of limonoid **24** computed from tandem MS data.
Figure S26. Fragmentation pattern of limonoid **25** computed from tandem MS data.
Figure S27. Fragmentation pattern of limonoid **26** computed from tandem MS data.
Figure S28. Fragmentation pattern of limonoid **27** computed from tandem MS data.
Figure S29. Fragmentation pattern of limonoid **28** computed from tandem MS data.
Figure S30. Fragmentation pattern of limonoid **29** computed from tandem MS data.
Figure S31. Fragmentation pattern of limonoid **30** computed from tandem MS data.
Figure S32. Fragmentation pattern of limonoid **31** computed from tandem MS data.
Figure S33. Fragmentation pattern of limonoid **32** computed from tandem MS data.

Figure S34. Fragmentation pattern of limonoid **33** computed from tandem MS data.
Figure S35. Fragmentation pattern of limonoid **34** computed from tandem MS data.
Figure S36. Fragmentation pattern of limonoid **35** computed from tandem MS data.
Figure S37. Fragmentation pattern of limonoid **36** computed from tandem MS data.

Table S1. HRMS and MS/MS data of the metabolites in *S. macrophylla* leaves extract detected by UPLC–MS/MS.

Limonoid	RT (min)	$[M + H]^+$		Molecular Formula	Error (ppm)	MS² fragments (Intensity)	Limonoid name
		Theoretical mass (Da)	Accurate mass (Da)				
Standard							
**1**	10.7	773.3021	773.3009	$C_{39}H_{48}O_{16}$	1.55	755.2943 (100), 671.2351 (1.78), 653.2260 (0.84), 571.1826 (0.41), 553.1697 (0.41), 511.1585 (2.39), 493.1478 (0.63)	12*α*-acetoxyl-20*β*,21*β*-22*α*,23*α*-diepoxyswietephragmin C
**2**	10.8	699.2653	699.2641	$C_{36}H_{42}O_{14}$	1.71	681.2563 (11.77), 639.2449 (17.08), 621.2339 (17.53), 603.2226 (7.68), 579.2230 (30.56), 561.2123 (36.99), 543.2010 (8.70), 539.1902 (19.05), 525.2115 (9.76), 521.1803 (7.13), 497.1787 (5.15), 483.1996 (5.75), 479.1691 (29.13), 465.1894 (9.18), 461.1585 (100)	2-deacetyl-6-acetoxyswietephragmin I
**3**	11.8	699.2653	699.2642	$C_{36}H_{42}O_{14}$	1.57	639.2445 (100), 621.2340 (69.27), 603.2225 (30.12), 579.2221 (3.70), 539.1913 (53.03), 521.1802 (73.98), 511.1935 (2.95), 503.1713 (8.58), 497.1794 (9.96), 495.2019 (3.71), 493.1846 (5.89), 479.1692 (85.86), 461.1577 (74.62), 451.1736 (16.62)	2-deacetyl-12*α*-acetoxyswietephragmin I
**4**	20.3	749.2809	749.2807	$C_{40}H_{44}O_{14}$	0.26	731.2728 (100), 661.2288 (2.78), 643.2185 (0.66), 635.2495 (2.78), 583.2158 (1.29), 539.1896 (0.43), 521.1814 (0.28), 479.1679 (1.29), 461.1568 (0.74)	3*β*-*O*-detigloyl-3*β*-*O*-benzoyl-6-*O*-acetylswietephragmin D
**5**	21.4	749.2809	749.2801	$C_{40}H_{44}O_{14}$	1.06	731.2728 (84.15), 689.2610 (2.99), 671.2506 (5.22), 661.2280 (0.93), 643.2156 (0.32), 601.2081 (0.67), 583.1951 (0.47), 575.2282 (0.75), 539.1898 (0.22), 487.1760 (0.23), 461.1576 (0.18)	3*β*-*O*-detigloyl-3*β*-*O*-benzoyl-12*α*-acetoxyswietephragmin D
**6**	21.5	727.2966	727.2962	$C_{38}H_{46}O_{14}$	0.55	709.2883 (100), 657.2626 (0.06), 639.2449 (1.03), 621.2331 (0.45), 613.2652 (1.03), 609.2310 (0.07), 603.2210 (0.07), 579.2211 (0.09), 561.2241 (0.07),	12*α*-acetoxyswietephragmin D

						539.1913 (0.39), 525.2061 (0.07), 521.1810 (0.28), 479.1678 (0.06), 461.1579 (0.52)	
**7**	22.9	727.2966	727.2958	$C_{38}H_{46}O_{14}$	1.1	709.2883 (100), 667.2769 (1.97), 649.2654 (2.86), 639.2439 (0.32), 621.2316 (0.22), 603.2237 (0.14), 579.2228 (0.43), 561.2112 (0.45), 553.2416 (0.49), 539.1931 (0.23), 465.1859 (0.18), 461.1578 (0.16)	6-*O*-acetyl-3′-demethylswietephragmin E
**8**	24.1	739.2966	739.2972	$C_{39}H_{46}O_{14}$	0.81	721.2885 (26.40), 679.2724 (0.13), 661.2657 (1.24), 639.2440 (0.39), 579.2263 (0.10), 561.2104 (0.14), 539.1929 (0.17)	12*α*-acetoxyl-8,9,30-*ortho*-tigloylate-swietemacrophine
**9**	25.6	763.2966	763.2964	$C_{41}H_{46}O_{14}$	0.26	745.2890 (100), 679.2402 (1.55), 661.2302 (10.34), 649.2664 (8.10), 643.2185 (1.56), 601.2065 (0.26), 583.2148 (1.12), 565.2051 (0.38), 539.1909 (0.84), 521.1804 (0.51), 479.1695 (2.42), 461.1579 (1.16)	3*β*-*O*-detigloyl-3*β*-*O*-benzoyl-6-*O*-acetylswietephragmin E
**10**	26.8	763.2966	763.2950	$C_{41}H_{46}O_{14}$	2.09	661.2267 (48.55), 601.2056 (6.58), 583.1955 (3.06), 565.1890 (3.28), 505.1830 (4.82), 487.1731 (5.69), 479.1690 (12.33), 461.1578 (35.71), 451.1730 (3.12)	3*β*-*O*-detigloyl-3*β*-*O*-benzoyl-12*α*-acetoxyswietephragmin C
**11**	26.9	741.3122	741.3123	$C_{39}H_{48}O_{14}$	0.13	723.3041 (100), 657.2567 (0.30), 639.2449 (4.20), 627.2815 (3.97), 621.2335 (1.19), 539.1906 (1.01), 521.1787 (0.50), 479.1689 (1.46), 461.1581 (1.07)	6-*O*-acetylswietephragmin E
**12**	28.3	741.3122	741.3120	$C_{39}H_{48}O_{14}$	0.26	723.3043 (89.06), 681.2928 (3.45), 663.2821 (5.78), 639.2446 (1.07), 579.2230 (1.35), 567.2587 (1.01), 561.2118 (0.73), 539.1907 (0.56)	12*α*-acetoxyswietephragmin C
Annotated							
**13**	2.68	731.2551	731.2551	$C_{36}H_{42}O_{16}$	0.0	713.2472 (100), 695.2362 (0.30), 671.2348 (0.91), 653.2240 (3.05), 635.2191 (0.14), 613.2025 (0.25), 593.2021 (0.74), 571.1804 (1.37), 553.1694 (1.49), 535.1584 (0.17), 511.1583 (2.35), 493.1482 (2.02), 479.1338 (0.18), 465.1530 (0.23), 461.1215 (0.21)	6-acetoxyl-20,21-22,23-diepoxyswietephragmin I

**14**	7.20	781.2708	781.2728	$C_{40}H_{44}O_{16}$	2.56	763.2651 (50.86), 703.2379 (32.99), 675.2128 (11.21), 633.1935 (21.32), 615.1919 (14.89), 553.1708 (22.85), 511.1583 (80.91), 493.1476 (100), 483.1660 (9.82), 479.1285 (27.87), 475.1340 (16.92), 465.1535 (17.88), 461.1219 (32.05), 451.1344 (9.96)	3-detigloyl-3-benzoyl-12-acetoxyl-20,21-22,23-diepoxyswietephragmin D
**15**	7.96	781.2708	781.2713	$C_{40}H_{44}O_{16}$	0.64	763.2504 (21.23), 693.2157 (5.42), 675.2056 (6.19), 615.1839 (19.31), 571.1768 (11.85), 553.1737 (7.59), 511.1581 (71.22), 493.1480 (100), 483.1666 (12.05), 479.1317 (13.47), 475.1360 (17.10), 465.1523 (20.72), 461.1218 (36.72), 451.1428 (8.57)	3-detigloyl-3-benzoyl-6-acetoxyl-20,21-22,23-diepoxyswietephragmin D
**16**	9.89	721.2496	721.2496	$C_{38}H_{40}O_{14}$	0.0	703.2388 (11.09), 661.2302 (16.29), 643.2188 (11.31), 0625.2103 (2.78), 607.2183 (79.79), 601.2081 (2.96), 565.2065 (77.95), 547.1956 (9.42), 539.1906 (19.78), 529.1844 (7.88), 521.1808 (10.75), 497.1788 (11.86), 479.1693 (53.03), 461.1585 (100), 451.1756 (3.75)	3-detigloyl-3-benzoyl-6-acetoxylswietephragmin I
**17***	10.8	681.2547	681.2535	$C_{36}H_{40}O_{13}$	1.76	639.2441 (23.21), 621.2336 (48.11), 603.2233 (16.68), 579.2217 (9.42), 539.1906 (75.13), 525.2102 (14.98), 521.1799 (51.70), 497.1802 (6.45), 493.1865 (12.12), 479.1673 (45.84), 461.1583 (68.58)	2-dehydroxyl-12-acetoxyswietephragmin I
**18**	10.8	795.2864	795.2845	$C_{41}H_{46}O_{16}$	2.38	735.2668 (4.60), 633.1978 (14.61), 615.1875 (7.70), 511.1584 (41.88), 493.1477 (100), 483.1626 (13.54), 479.1299 (14.01), 475.1375 (18.72), 469.1467 (5.25), 465.1540 (53.41), 461.1231 (35.36), 459.1805 (4.68), 451.1385 (32.58)	3-detigloyl-3-benzoyl-6-acetoxyl-20,21-22,23-diepoxyswietephragmin E
**19***	11.1	623.2492	623.2483	$C_{34}H_{38}O_{11}$	1.44	581.2389 (0.24), 563.2278 (4.39), 545.2165 (0.16), 481.1844 (0.68), 467.2045 (0.18), 463.1735 (1.50)	2-dehydroxyswietephragmin G
**20**	11.6	773.3021	773.3006	$C_{39}H_{48}O_{16}$	1.93	755.2931 (100), 737.2772 (4.97), 671.2360 (13.97), 653.2255 (12.90), 593.2033 (18.85), 571.1821 (19.68), 561.1708 (4.99), 553.1685 (15.49), 511.1596 (71.13), 493.1477 (95.18), 479.1331 (17.84), 475.1399 (10.80), 465.1543 (13.21), 461.1217 (34.43)	2-dehydroxyl-6-hydroxyl-12-acetoxyl-20,21-22,23-diepoxyswietephragmin C

**21**	12.5	739.2966	739.2958	$C_{39}H_{46}O_{14}$	1.08	721.2869 (25.40), 639.2456 (0.81), 621.2408 (0.18), 539.1898 (0.15), 521.1777 (0.12), 479.1683 (0.24), 461.1589 (0.12)	2-dehydroxyl-6-acetoxyl-12-hydroxyl-8,9,30-*ortho*-tigloylate-swietemacrophine
**22**	13.1	761.2809	761.2801	$C_{41}H_{44}O_{14}$	1.05	743.2725 (23.01), 701.2587 (0.31), 683.2508 (1.63), 661.2281 (0.68), 643.2175 (0.10), 639.2410 (0.03), 601.2098 (0.13), 587.2244 (0.12), 583.1941 (0.14), 539.1986 (0.08), 521.1827 (0.03), 497.1748 (0.02), 493.1855 (0.07), 487.1740 (0.08), 479.1668 (0.04), 465.1853 (0.02), 461.1576 (0.08)	2-dehydroxyl-3-detigloyl-3-benzoyl-6-acetoxyl-12-hydroxyl-8,9,30-*ortho*-tigloylate-swietemacrophine
**23***	13.3	681.2547	681.2552	$C_{36}H_{40}O_{13}$	0.73	639.2402 (6.37), 621.2333 (7.94), 579.2247 (9.13), 561.2117 (24.01), 539.1914 (32.83), 521.1778 (28.94), 503.1704 (17.10), 493.1833 (10.26), 479.1683 (45.11), 465.1900 (16.46), 461.1580 (61.97)	2-dehydroxyl-6-acetoxyswietephragmin I
**24**	13.4	773.3021	773.3012	$C_{39}H_{48}O_{16}$	1.16	755.2939 (61.20), 671.2369 (0.53), 653.2250 (1.31), 611.2106 (0.14), 593.2131 (0.08), 571.1783 (0.10), 553.1721 (0.05), 511.1610 (0.26), 493.1504 (0.06), 479.1349 (0.13), 475.1290 (0.14)	6-acetoxyl-20,21-22,23-diepoxyswietephragmin E
**25**	14.2	739.2966	739.2961	$C_{39}H_{46}O_{14}$	0.67	721.2883 (27.66), 679.2798 (0.15), 661.2664 (1.45), 639.2457 (0.53), 621.2319 (0.08), 603.2207 (0.03), 597.2354 (0.02), 579.2224 (0.12), 565.2438 (0.10), 561.2102 (0.15), 539.1891 (0.19), 521.1819 (0.02), 493.1864 (0.02), 483.2010 (0.03), 479.1696 (0.05), 465.1867 (0.08), 461.1588 (0.05)	6-acetoxyl-12-deacetoxyl-8,9,30-*ortho*-tigloylate-swietemacrophine
**26**	14.4	735.2653	735.2664	$C_{39}H_{42}O_{14}$	1.49	717.2563 (14.68), 679.2400 (3.17), 661.2306 (25.45), 643.2184 (12.84), 625.2101 (3.71), 621.2348 (55.42), 601.2087 (3.73), 583.2150 (2.82), 565.2072 (100), 557.1965 (2.63), 547.1906 (17.18), 539.1909 (18.22), 529.1876 (6.46), 521.1797 (11.33), 497.1775 (10.85), 479.1685 (61.81), 461.1582 (98.12), 451.1740 (5.25)	3-detigloyl-3-benzoyl-12-acetoxyswietephragmin J
**27**	15.2	763.2966	763.2965	$C_{41}H_{46}O_{14}$	0.13	745.2875 (32.53), 703.2760 (11.78), 685.2656 (21.65), 661.2296 (10.52), 619.2180 (14.04), 601.2074 (100), 589.2418 (9.43), 583.1963 (34.69), 565.1854 (7.99), 539.1920 (7.50), 505.1837 (5.95), 487.1734 (22.00), 479.1685 (16.70), 461.1580 (53.85)	2-dehydroxyl-3-detigloyl-3-benzoyl-6-hydroxy-12-acetoxyswietephragmin C

**28**	15.4	735.2653	735.2651	$C_{39}H_{42}O_{14}$	0.27	717.2570 (100), 675.2446 (5.66), 661.2285 (0.78), 657.2336 (8.03), 643.2203 (0.19), 625.2021 (0.23), 619.2142 (0.16), 615.2366 (0.12), 601.2089 (0.82), 583.1938 (0.64), 565.1865 (0.17), 561.2112 (1.57), 539.1890 (0.19), 521.1834 (0.13), 487.1721 (0.26), 469.1534 (0.13), 461.1657 (0.19)	3-detigloyl-3-benzoyl-6-acetoxyswietephragmin J
**29***	15.5	695.2704	695.2703	$C_{37}H_{42}O_{13}$	0.14	639.2447 (0.03), 635.2346 (0.02), 621.2318 (0.24), 595.2141 (0.01), 579.2221 (0.02), 561.2120 (0.06), 543.1964 (0.01), 539.2025 (0.02), 533.2236 (0.02), 521.1804 (0.12), 517.2255 (0.02), 503.1706 (0.03), 483.1984 (0.01), 479.1680 (0.04), 465.1890 (0.05), 461.1584 (0.06)	2-dehydroxyl-6-acetoxyswietephragmin H
**30**	15.5	713.2809	713.2812	$C_{37}H_{44}O_{14}$	0.42	695.2706 (21.56), 639.2442 (11.49), 621.2332 (8.19), 599.2496 (23.94), 565.2441 (12.18), 543.2214 (63.34), 539.1906 (44.11), 525.2091 (6.11), 521.1771 (15.46), 497.1787 (6.79), 479.1684 (42.97), 461.1580 (100)	12-acetoxyswietephragmin J
**31**	16.4	741.3122	741.3121	$C_{39}H_{48}O_{14}$	0.13	723.3035 (100), 657.2541 (0.45), 639.2446 (1.55), 627.2798 (0.98), 621.2320 (0.66), 579.2173 (0.17), 539.1909 (0.42), 521.1719 (0.16), 479.1682 (0.60), 461.1587 (0.43)	2-dehydroxyl-6-acetoxyl-12-hydroxyswietephragmin E
**32**	16.7	713.2809	713.2810	$C_{37}H_{44}O_{14}$	0.14	695.2714 (31.48), 653.2609 (15.53), 639.2447 (3.94), 635.2509 (17.50), 603.2239 (8.24), 597.2304 (3.47), 579.2234 (43.48), 561.2118 (40.83), 553.2045 (6.64), 543.2022 (9.78), 539.2282 (17.89), 535.1922 (4.99), 521.1820 (5.61), 497.1783 (7.55), 483.2017 (6.66), 479.1695 (39.02), 465.1891 (14.50), 461.1593 (100)	6-acetoxyswietephragmin J
**33**	22.6	739.2966	739.2977	$C_{39}H_{46}O_{14}$	1.48	721.2882 (100), 639.2453 (8.42), 625.2659 (11.83), 621.2325 (3.23), 539.1913 (18.34), 525.2119 (3.32), 521.1795 (7.31), 497.1816 (2.48), 479.1689 (21.23), 461.1583 (33.70)	2-dehydroxyl-6-hydroxyl-12-acetoxyl-8,9,30-*ortho*-tigloylate-swietemacrophine
**34**	22.6	761.2809	761.2812	$C_{41}H_{44}O_{14}$	0.39	743.2717 (100), 661.2278 (21.88), 647.2474 (33.42), 643.2195 (11.95), 625.2043 (2.42), 565.2054 (7.59), 547.1970 (8.30), 539.1901 (12.06), 529.1814 (2.54),	3-detigloyl-3-benzoyl-12-acetoxyl-6-deacetoxyl-8,9,30-*ortho*-tigloylate-swietemacrophine

						521.1765 (8.95), 497.1825 (4.68), 479.1682 (42.73), 461.1581 (45.54), 451.1700 (4.84)	
**35**	24.5	705.2911	705.2918	$C_{39}H_{44}O_{12}$	0.98	687.2826 (47.29), 669.2675 (0.34), 621.2338 (0.78), 603.2215 (3.42), 591.2638 (2.28), 585.2136 (0.96), 525.2098 (0.62), 507.1990 (0.22), 481.1842 (0.78), 463.1718 (1.05)	2-hydroxy-3-detigloyl-3-benzoyl-swietephragmin C
**36**	26.0	683.3068	683.3069	$C_{37}H_{46}O_{12}$	0.14	665.2974 (72.74), 647.2832 (0.12), 599.2455 (0.22), 581.2372 (2.40), 569.2735 (1.54), 563.2245 (0.86), 521.2119 (0.13), 481.1845 (0.90), 463.1740 (1.06)	2-dehydroxyl-12-hydroxyswietephragmin C

* The positions of the acetate group and the double bond can be interchanged.

Table S2. *In-house* database containing 270 combinations of putative phragmalin-type limonoids.

Base structural for biosynthetic proposition of phragmalin-type limonoids

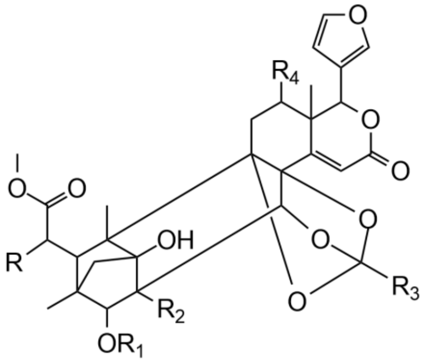

1-270

ID	Molecular Formula	Mass		Functional group[1]	Smiles	References
		Neutral	$[M + H]^+$ theorical			
**1**	$C_{34}H_{40}O_{12}$	640.2520	641.2598	R = H. R_1 = Tg. R_2 = OH. R_3 = CH_3. R_4 = H	OC12C3(O)C(OC(/C(C)=C/C)=O)C(C2)(C)C(C([H])C(OC)=O)C1(C)C45C(C(C6(C)C([H])C5)=CC(OC6C7=COC=C7)=O)(O8)C3OC8(C)O4	[1]
**2**	$C_{36}H_{38}O_{12}$	662.2363	663.2442	R = H. R_1 = Bz. R_2 = OH. R_3 = CH_3. R_4 = H	OC12C3(O)C(OC(C4=CC=CC=C4)=O)C(C2)(C)C(C([H])C(OC)=O)C1(C)C56C(C(C7(C)C([H])C6)=CC(OC7C8=COC=C8)=O)(O9)C3OC9(C)O5	

**3**	$C_{36}H_{42}O_{14}$	698.2575	699.2653	R = OAc. R_1 = Tg. R_2 = OH. R_3 = CH_3. R_4 = H	OC12C3(O)C(OC(/C(C)=C/C)=O)C(C2)(C)C(C(OC(C)=O)C(OC)=O)C1(C)C45C(C(C6(C)C([H])C5)=CC(OC6C7=COC=C7)=O)(O8)C3OC8(C)O4	[2]
**4**	$C_{38}H_{40}O_{14}$	720.2418	721.2496	R = OAc. R_1 = Bz. R_2 = OH. R_3 = CH_3. R_4 = H	OC12C3(O)C(OC(C4=CC=CC=C4)=O)C(C2)(C)C(C(OC(C)=O)C(OC)=O)C1(C)C56C(C(C7(C)C([H])C6)=CC(OC7C8=COC=C8)=O)(O9)C3OC9(C)O5	
**5**	$C_{36}H_{42}O_{13}$	682.2625	683.2704	R = H. R_1 = Tg. R_2 = OAc. R_3 = CH_3. R_4 = H	OC12C3(OC(C)=O)C(OC(/C(C)=C/C)=O)C(C2)(C)C(C([H])C(OC)=O)C1(C)C45C(C(C6(C)C([H])C5)=CC(OC6C7=COC=C7)=O)(O8)C3OC8(C)O4	[3,4]
**6**	$C_{38}H_{40}O_{13}$	704.2469	705.2547	R = H. R_1 = Bz. R_2 = OAc. R_3 = CH_3. R_4 = H	OC12C3(OC(C)=O)C(OC(C4=CC=CC=C4)=O)C(C2)(C)C(C([H])C(OC)=O)C1(C)C56C(C(C7(C)C([H])C6)=CC(OC7C8=COC=C8)=O)(O9)C3OC9(C)O5	
**7**	$C_{36}H_{42}O_{14}$	698.2575	699.2653	R = H. R_1 = Tg. R_2 = OH. R_3 = CH_3. R_4 = OAc	OC12C3(O)C(OC(/C(C)=C/C)=O)C(C2)(C)C(C([H])C(OC)=O)C1(C)C45C(C(C6(C)C(OC(C)=O)C5)=CC(OC6C7=COC=C7)=O)(O8)C3OC8(C)O4	[2]
**8**	$C_{38}H_{40}O_{14}$	720.2418	721.2496	R = H. R_1 = Bz. R_2 = OH. R_3 = CH_3. R_4 = OAc	OC12C3(O)C(OC(C4=CC=CC=C4)=O)C(C2)(C)C(C([H])C(OC)=O)C1(C)C56C(C(C7(	

					C)C(OC(C)=O)C6)=CC(OC7C8=COC=C8)=O)(O9)C3OC9(C)O5	
**9**	$C_{38}H_{44}O_{15}$	740.2680	741.2758	R = OAc. R_1 = Tg. R_2 = OAc. R_3 = CH_3. R_4 = H	OC12C3(OC(C)=O)C(OC(/C(C)=C/C)=O)C(C2)(C)C(C(OC(C)=O)C(OC)=O)C1(C)C45C(C(C6(C)C([H])C5)=CC(OC6C7=COC=C7)=O)(O8)C3OC8(C)O4	
**10**	$C_{40}H_{42}O_{15}$	762.2524	763.2602	R = OAc. R_1 = Bz. R_2 = OAc. R_3 = CH_3. R_4 = H	OC12C3(OC(C)=O)C(OC(C4=CC=CC=C4)=O)C(C2)(C)C(C(OC(C)=O)C(OC)=O)C1(C)C56C(C(C7(C)C([H])C6)=CC(OC7C8=COC=C8)=O)(O9)C3OC9(C)O5	
**11**	$C_{38}H_{44}O_{16}$	756.2629	757.2708	R = OAc. R_1 = Tg. R_2 = OH. R_3 = CH_3. R_4 = OAc	OC12C3(O)C(OC(/C(C)=C/C)=O)C(C2)(C)C(C(OC(C)=O)C(OC)=O)C1(C)C45C(C(C6(C)C(OC(C)=O)C5)=CC(OC6C7=COC=C7)=O)(O8)C3OC8(C)O4	
**12**	$C_{40}H_{42}O_{16}$	778.2473	779.2551	R = OAc. R_1 = Bz. R_2 = OH. R_3 = CH_3. R_4 = OAc	OC12C3(O)C(OC(C4=CC=CC=C4)=O)C(C2)(C)C(C(OC(C)=O)C(OC)=O)C1(C)C56C(C(C7(C)C(OC(C)=O)C6)=CC(OC7C8=COC=C8)=O)(O9)C3OC9(C)O5	
**13**	$C_{38}H_{44}O_{15}$	740.2680	741.2758	R = H. R_1 = Tg. R_2 = OAc. R_3 = CH_3. R_4 = OAc	OC12C3(OC(C)=O)C(OC(/C(C)=C/C)=O)C(C2)(C)C(C([H])C(OC)=O)C1(C)C45C(C(C6(C)C(OC(C)=O)C5)=CC(OC6C7=COC=C7)=O)(O8)C3OC8(C)O4	[2]

**14**	$C_{40}H_{42}O_{15}$	762.2524	763.2602	R = H. R_1 = Bz. R_2 = OAc. R_3 = CH_3. R_4 = OAc	OC12C3(OC(C)=O)C(OC(C4=CC=CC=C4)=O)C(C2)(C)C(C([H])C(OC)=O)C1(C)C56C(C(C7(C)C(OC(C)=O)C6)=CC(OC7C8=COC=C8)=O)(O9)C3OC9(C)O5	[2]
**15**	$C_{40}H_{46}O_{17}$	798.2735	799.2813	R = OAc. R_1 = Tg. R_2 = OAc. R_3 = CH_3. R_4 = OAc	OC12C3(OC(C)=O)C(OC(/C(C)=C/C)=O)C(C2)(C)C(C(OC(C)=O)C(OC)=O)C1(C)C45C(C(C6(C)C(OC(C)=O)C5)=CC(OC6C7=COC=C7)=O)(O8)C3OC8(C)O4	
**16**	$C_{42}H_{44}O_{17}$	820.2578	821.2657	R = OAc. R_1 = Bz. R_2 = OAc. R_3 = CH_3. R_4 = OAc	OC12C3(OC(C)=O)C(OC(C4=CC=CC=C4)=O)C(C2)(C)C(C(OC(C)=O)C(OC)=O)C1(C)C56C(C(C7(C)C(OC(C)=O)C6)=CC(OC7C8=COC=C8)=O)(O9)C3OC9(C)O5	
**17**	$C_{35}H_{42}O_{12}$	654.2676	655.2755	R = H. R_1 = Tg. R_2 = OH. R_3 = CH_2CH_3. R_4 = H	OC12C3(O)C(OC(/C(C)=C/C)=O)C(C2)(C)C(C([H])C(OC)=O)C1(C)C45C(C(C6(C)C([H])C5)=CC(OC6C7=COC=C7)=O)(O8)C3OC8(CC)O4	[1]
**18**	$C_{37}H_{40}O_{12}$	676.2520	677.2598	R = H. R_1 = Bz. R_2 = OH. R_3 = CH_2CH_3. R_4 = H	OC12C3(O)C(OC(C4=CC=CC=C4)=O)C(C2)(C)C(C([H])C(OC)=O)C1(C)C56C(C(C7(C)C([H])C6)=CC(OC7C8=COC=C8)=O)(O9)C3OC9(CC)O5	
**19**	$C_{37}H_{44}O_{14}$	712.2731	713.2809	R = OAc. R_1 = Tg. R_2 = OH. R_3 = CH_2CH_3. R_4 = H	OC12C3(O)C(OC(/C(C)=C/C)=O)C(C2)(C)C(C(OC(C)=O)C(OC)=O)C1(C)C45C(C(C6	

					(C)C([H])C5)=CC(OC6C7=COC=C7)=O)(O8)C3OC8(CC)O4	
**20**	$C_{39}H_{42}O_{14}$	734.2575	735.2653	R = OAc. R_1 = Bz. R_2 = OH. R_3 = CH_2CH_3. R_4 = H	OC12C3(O)C(OC(C4=CC=CC=C4)=O)C(C2)(C)C(C(OC(C)=O)C(OC)=O)C1(C)C56C(C(C7(C)C([H])C6)=CC(OC7C8=COC=C8)=O)(O9)C3OC9(CC)O5	
**21**	$C_{37}H_{44}O_{13}$	696.2782	697.2860	R = H. R_1 = Tg. R_2 = OAc. R_3 = CH_2CH_3. R_4 = H	OC12C3(OC(C)=O)C(OC(/C(C)=C/C)=O)C(C2)(C)C(C([H])C(OC)=O)C1(C)C45C(C(C6(C)C([H])C5)=CC(OC6C7=COC=C7)=O)(O8)C3OC8(CC)O4	[3,5]
**22**	$C_{39}H_{42}O_{13}$	718.2625	719.2704	R = H. R_1 = Bz. R_2 = OAc. R_3 = CH_2CH_3. R_4 = H	OC12C3(OC(C)=O)C(OC(C4=CC=CC=C4)=O)C(C2)(C)C(C([H])C(OC)=O)C1(C)C56C(C(C7(C)C([H])C6)=CC(OC7C8=COC=C8)=O)(O9)C3OC9(CC)O5	
**23**	$C_{37}H_{44}O_{14}$	712.2731	713.2809	R = H. R_1 = Tg. R_2 = OH. R_3 = CH_2CH_3. R_4 = OAc	OC12C3(O)C(OC(/C(C)=C/C)=O)C(C2)(C)C(C([H])C(OC)=O)C1(C)C45C(C(C6(C)C(OC(C)=O)C5)=CC(OC6C7=COC=C7)=O)(O8)C3OC8(CC)O4	
**24**	$C_{39}H_{42}O_{14}$	734.2575	735.2653	R = H. R_1 = Bz. R_2 = OH. R_3 = CH_2CH_3. R_4 = OAc	OC12C3(O)C(OC(C4=CC=CC=C4)=O)C(C2)(C)C(C([H])C(OC)=O)C1(C)C56C(C(C7(C)C(OC(C)=O)C6)=CC(OC7C8=COC=C8)=O)(O9)C3OC9(CC)O5	

**25**	$C_{39}H_{46}O_{15}$	754.2837	755.2915	R = OAc. R_1 = Tg. R_2 = OAc. R_3 = CH_2CH_3. R_4 = H	OC12C3(OC(C)=O)C(OC(/C(C)=C/C)=O)C(C2)(C)C(C(OC(C)=O)C(OC)=O)C1(C)C45C(C(C6(C)C([H])C5)=CC(OC6C7=COC=C7)=O)(O8)C3OC8(CC)O4
**26**	$C_{41}H_{44}O_{15}$	776.2680	777.2758	R = OAc. R_1 = Bz. R_2 = OAc. R_3 = CH_2CH_3. R_4 = H	OC12C3(OC(C)=O)C(OC(C4=CC=CC=C4)=O)C(C2)(C)C(C(OC(C)=O)C(OC)=O)C1(C)C56C(C(C7(C)C([H])C6)=CC(OC7C8=COC=C8)=O)(O9)C3OC9(CC)O5
**27**	$C_{39}H_{46}O_{16}$	770.2786	771.2864	R = OAc. R_1 = Tg. R_2 = OH. R_3 = CH_2CH_3. R_4 = OAc	OC12C3(O)C(OC(/C(C)=C/C)=O)C(C2)(C)C(C(OC(C)=O)C(OC)=O)C1(C)C45C(C(C6(C)C(OC(C)=O)C5)=CC(OC6C7=COC=C7)=O)(O8)C3OC8(CC)O4
**28**	$C_{41}H_{44}O_{16}$	792.2629	793.2708	R = OAc. R_1 = Bz. R_2 = OH. R_3 = CH_2CH_3. R_4 = OAc	OC12C3(O)C(OC(C4=CC=CC=C4)=O)C(C2)(C)C(C(OC(C)=O)C(OC)=O)C1(C)C56C(C(C7(C)C(OC(C)=O)C6)=CC(OC7C8=COC=C8)=O)(O9)C3OC9(CC)O5
**29**	$C_{39}H_{46}O_{15}$	754.2837	755.2915	R = H. R_1 = Tg. R_2 = OAc. R_3 = CH_2CH_3. R_4 = OAc	OC12C3(OC(C)=O)C(OC(/C(C)=C/C)=O)C(C2)(C)C(C([H])C(OC)=O)C1(C)C45C(C(C6(C)C(OC(C)=O)C5)=CC(OC6C7=COC=C7)=O)(O8)C3OC8(CC)O4
**30**	$C_{41}H_{44}O_{15}$	776.2680	777.2758	R = H. R_1 = Bz. R_2 = OAc. R_3 = CH_2CH_3. R_4 = OAc	OC12C3(OC(C)=O)C(OC(C4=CC=CC=C4)=O)C(C2)(C)C(C([H])C(OC)=O)C1(C)C56

					C(C(C7(C)C(OC(C)=O)C6)=CC(OC7C8=C OC=C8)=O)(O9)C3OC9(CC)O5	
**31**	$C_{41}H_{48}O_{17}$	812.2892	813.2970	R = OAc. R_1 = Tg. R_2 = OAc. R_3 = CH_2CH_3. R_4 = OAc	OC12C3(OC(C)=O)C(OC(/C(C)=C/C)=O) C(C2)(C)C(C(OC(C)=O)C(OC)=O)C1(C)C 45C(C(C6(C)C(OC(C)=O)C5)=CC(OC6C7 =COC=C7)=O)(O8)C3OC8(CC)O4	
**32**	$C_{43}H_{46}O_{17}$	834.2735	835.2813	R = OAc. R_1 = Bz. R_2 = OAc. R_3 = CH_2CH_3. R_4 = OAc	OC12C3(OC(C)=O)C(OC(C4=CC=CC=C4) =O)C(C2)(C)C(C(OC(C)=O)C(OC)=O)C1(C)C56C(C(C7(C)C(OC(C)=O)C6)=CC(OC 7C8=COC=C8)=O)(O9)C3OC9(CC)O5	
**33**	$C_{36}H_{44}O_{12}$	668.2833	669.2911	R = H. R_1 = Tg. R_2 = OH. R_3 = $CH(CH_3)_2$. R_4 = H	OC12C3(O)C(OC(/C(C)=C/C)=O)C(C2)(C) C(C([H])C(OC)=O)C1(C)C45C(C(C6(C)C([H])C5)=CC(OC6C7=COC=C7)=O)(O8)C3 OC8(C(C)C)O4	[1]
**34**	$C_{38}H_{42}O_{12}$	690.2676	691.2755	R = H. R_1 = Bz. R_2 = OH. R_3 = $CH(CH_3)_2$. R_4 = H	OC12C3(O)C(OC(C4=CC=CC=C4)=O)C(C 2)(C)C(C([H])C(OC)=O)C1(C)C56C(C(C7(C)C([H])C6)=CC(OC7C8=COC=C8)=O)(O 9)C3OC9(C(C)C)O5	
**35**	$C_{38}H_{46}O_{14}$	726.2888	727.2966	R = OAc. R_1 = Tg. R_2 = OH. R_3 = $CH(CH_3)_2$. R_4 = H	OC12C3(O)C(OC(/C(C)=C/C)=O)C(C2)(C) C(C(OC(C)=O)C(OC)=O)C1(C)C45C(C(C6 (C)C([H])C5)=CC(OC6C7=COC=C7)=O)(O8)C3OC8(C(C)C)O4	[2,6]

**36**	$C_{40}H_{44}O_{14}$	748.2731	749.2809	R = OAc. R_1 = Bz. R_2 = OH. R_3 = $CH(CH_3)_2$. R_4 = H	OC12C3(O)C(OC(C4=CC=CC=C4)=O)C(C2)(C)C(C(OC(C)=O)C(OC)=O)C1(C)C56C(C(C7(C)C([H])C6)=CC(OC7C8=COC=C8)=O)(O9)C3OC9(C(C)C)O5	[2]
**37**	$C_{38}H_{46}O_{13}$	710.2938	711.3017	R = H. R_1 = Tg. R_2 = OAc. R_3 = $CH(CH_3)_2$. R_4 = H	OC12C3(OC(C)=O)C(OC(/C(C)=C/C)=O)C(C2)(C)C(C([H])C(OC)=O)C1(C)C45C(C(C6(C)C([H])C5)=CC(OC6C7=COC=C7)=O)(O8)C3OC8(C(C)C)O4	[1]
**38**	$C_{40}H_{44}O_{13}$	732.2782	733.2860	R = H. R_1 = Bz. R_2 = OAc. R_3 = $CH(CH_3)_2$. R_4 = H	OC12C3(OC(C)=O)C(OC(C4=CC=CC=C4)=O)C(C2)(C)C(C([H])C(OC)=O)C1(C)C56C(C(C7(C)C([H])C6)=CC(OC7C8=COC=C8)=O)(O9)C3OC9(C(C)C)O5	
**39**	$C_{38}H_{46}O_{14}$	726.2888	727.2966	R = H. R_1 = Tg. R_2 = OH. R_3 = $CH(CH_3)_2$. R_4 = OAc	OC12C3(O)C(OC(/C(C)=C/C)=O)C(C2)(C)C(C([H])C(OC)=O)C1(C)C45C(C(C6(C)C(OC(C)=O)C5)=CC(OC6C7=COC=C7)=O)(O8)C3OC8(C(C)C)O4	[2,7]
**40**	$C_{40}H_{44}O_{14}$	748.2731	749.2809	R = H. R_1 = Bz. R_2 = OH. R_3 = $CH(CH_3)_2$. R_4 = OAc	OC12C3(O)C(OC(C4=CC=CC=C4)=O)C(C2)(C)C(C([H])C(OC)=O)C1(C)C56C(C(C7(C)C(OC(C)=O)C6)=CC(OC7C8=COC=C8)=O)(O9)C3OC9(C(C)C)O5	[2,7]
**41**	$C_{40}H_{48}O_{15}$	768.2998	769.3071	R = OAc. R_1 = Tg. R_2 = OAc. R_3 = $CH(CH_3)_2$. R_4 = H	OC12C3(OC(C)=O)C(OC(/C(C)=C/C)=O)C(C2)(C)C(C(OC(C)=O)C(OC)=O)C1(C)C	

					45C(C(C6(C)C([H])C5)=CC(OC6C7=COC=C7)=O)(O8)C3OC8(C(C)C)O4
**42**	$C_{42}H_{46}O_{15}$	790.2837	791.2915	R = OAc. R_1 = Bz. R_2 = OAc. R_3 = $CH(CH_3)_2$. R_4 = H	OC12C3(OC(C)=O)C(OC(C4=CC=CC=C4)=O)C(C2)(C)C(C(OC(C)=O)C(OC)=O)C1(C)C56C(C(C7(C)C([H])C6)=CC(OC7C8=COC=C8)=O)(O9)C3OC9(C(C)C)O5
**43**	$C_{40}H_{48}O_{16}$	784.2942	785.3021	R = OAc. R_1 = Tg. R_2 = OH. R_3 = $CH(CH_3)_2$. R_4 = OAc	OC12C3(O)C(OC(/C(C)=C/C)=O)C(C2)(C)C(C(OC(C)=O)C(OC)=O)C1(C)C45C(C(C6(C)C(OC(C)=O)C5)=CC(OC6C7=COC=C7)=O)(O8)C3OC8(C(C)C)O4
**44**	$C_{42}H_{46}O_{16}$	806.2786	807.2864	R = OAc. R_1 = Bz. R_2 = OH. R_3 = $CH(CH_3)_2$. R_4 = OAc	OC12C3(O)C(OC(C4=CC=CC=C4)=O)C(C2)(C)C(C(OC(C)=O)C(OC)=O)C1(C)C56C(C(C7(C)C(OC(C)=O)C6)=CC(OC7C8=COC=C8)=O)(O9)C3OC9(C(C)C)O5
**45**	$C_{40}H_{48}O_{15}$	768.2993	769.3071	R = H. R_1 = Tg. R_2 = OAc. R_3 = $CH(CH_3)_2$. R_4 = OAc	OC12C3(OC(C)=O)C(OC(/C(C)=C/C)=O)C(C2)(C)C(C([H])C(OC)=O)C1(C)C45C(C(C6(C)C(OC(C)=O)C5)=CC(OC6C7=COC=C7)=O)(O8)C3OC8(C(C)C)O4
**46**	$C_{42}H_{46}O_{15}$	790.2837	791.2915	R = H. R_1 = Bz. R_2 = OAc. R_3 = $CH(CH_3)_2$. R_4 = OAc	OC12C3(OC(C)=O)C(OC(C4=CC=CC=C4)=O)C(C2)(C)C(C([H])C(OC)=O)C1(C)C56C(C(C7(C)C(OC(C)=O)C6)=CC(OC7C8=COC=C8)=O)(O9)C3OC9(C(C)C)O5

**47**	$C_{42}H_{50}O_{17}$	826.3048	827.3126	R = OAc. R_1 = Tg. R_2 = OAc. R_3 = $CH(CH_3)_2$. R_4 = OAc	OC12C3(OC(C)=O)C(OC(/C(C)=C/C)=O)C(C2)(C)C(C(OC(C)=O)C(OC)=O)C1(C)C45C(C(C6(C)C(OC(C)=O)C5)=CC(OC6C7=COC=C7)=O)(O8)C3OC8(C(C)C)O4	
**48**	$C_{44}H_{48}O_{17}$	848.2892	849.2970	R = OAc. R_1 = Bz. R_2 = OAc. R_3 = $CH(CH_3)_2$. R_4 = OAc	OC12C3(OC(C)=O)C(OC(C4=CC=CC=C4)=O)C(C2)(C)C(C(OC(C)=O)C(OC)=O)C1(C)C56C(C(C7(C)C(OC(C)=O)C6)=CC(OC7C8=COC=C8)=O)(O9)C3OC9(C(C)C)O5	
**49**	$C_{37}H_{46}O_{12}$	682.2989	683.3068	R = H. R_1 = Tg. R_2 = OH. R_3 = $CH_3CHCH_2CH_3$. R_4 = H	OC12C3(O)C(OC(/C(C)=C/C)=O)C(C2)(C)C(C([H])C(OC)=O)C1(C)C45C(C(C6(C)C([H])C5)=CC(OC6C7=COC=C7)=O)(O8)C3OC8(C(C)CC)O4	[8]
**50**	$C_{39}H_{44}O_{12}$	704.2833	705.2911	R = H. R_1 = Bz. R_2 = OH. R_3 = $CH_3CHCH_2CH_3$. R_4 = H	OC12C3(O)C(OC(C4=CC=CC=C4)=O)C(C2)(C)C(C([H])C(OC)=O)C1(C)C56C(C(C7(C)C([H])C6)=CC(OC7C8=COC=C8)=O)(O9)C3OC9(C(C)CC)O5	
**51**	$C_{39}H_{48}O_{14}$	740.3044	741.3122	R = OAc. R_1 = Tg. R_2 = OH. R_3 = $CH_3CHCH_2CH_3$. R_4 = H	OC12C3(O)C(OC(/C(C)=C/C)=O)C(C2)(C)C(C(OC(C)=O)C(OC)=O)C1(C)C45C(C(C6(C)C([H])C5)=CC(OC6C7=COC=C7)=O)(O8)C3OC8(C(C)CC)O4	[2,7]
**52**	$C_{41}H_{46}O_{14}$	762.2888	763.2966	R = OAc. R_1 = Bz. R_2 = OH. R_3 = $CH_3CHCH_2CH_3$. R_4 = H	OC12C3(O)C(OC(C4=CC=CC=C4)=O)C(C2)(C)C(C(OC(C)=O)C(OC)=O)C1(C)C56C(	[2,7]

					C(C7(C)C([H])C6)=CC(OC7C8=COC=C8)=O)(O9)C3OC9(C(C)CC)O5	
**53**	$C_{39}H_{48}O_{13}$	724.3095	725.3173	R = H. R_1 = Tg. R_2 = OAc. R_3 = $CH_3CHCH_2CH_3$. R_4 = H	OC12C3(OC(C)=O)C(OC(/C(C)=C/C)=O)C(C2)(C)C(C([H])C(OC)=O)C1(C)C45C(C(C6(C)C([H])C5)=CC(OC6C7=COC=C7)=O)(O8)C3OC8(C(C)CC)O4	[1]
**54**	$C_{41}H_{46}O_{13}$	746.2938	747.3017	R = H. R_1 = Bz. R_2 = OAc. R_3 = $CH_3CHCH_2CH_3$. R_4 = H	OC12C3(OC(C)=O)C(OC(C4=CC=CC=C4)=O)C(C2)(C)C(C([H])C(OC)=O)C1(C)C56C(C(C7(C)C([H])C6)=CC(OC7C8=COC=C8)=O)(O9)C3OC9(C(C)CC)O5	
**55**	$C_{39}H_{48}O_{14}$	740.3044	741.3122	R = H. R_1 = Tg. R_2 = OH. R_3 = $CH_3CHCH_2CH_3$. R_4 = OAc	OC12C3(O)C(OC(/C(C)=C/C)=O)C(C2)(C)C(C([H])C(OC)=O)C1(C)C45C(C(C6(C)C(OC(C)=O)C5)=CC(OC6C7=COC=C7)=O)(O8)C3OC8(C(C)CC)O4	[2,7,8]
**56**	$C_{41}H_{46}O_{14}$	762.2888	763.2966	R = H. R_1 = Bz. R_2 = OH. R_3 = $CH_3CHCH_2CH_3$. R_4 = OAc	OC12C3(O)C(OC(C4=CC=CC=C4)=O)C(C2)(C)C(C([H])C(OC)=O)C1(C)C56C(C(C7(C)C(OC(C)=O)C6)=CC(OC7C8=COC=C8)=O)(O9)C3OC9(C(C)CC)O5	[2,7]
**57**	$C_{41}H_{50}O_{15}$	782.3150	783.3228	R = OAc. R_1 = Tg. R_2 = OAc. R_3 = $CH_3CHCH_2CH_3$. R_4 = H	OC12C3(OC(C)=O)C(OC(/C(C)=C/C)=O)C(C2)(C)C(C(OC(C)=O)C(OC)=O)C1(C)C45C(C(C6(C)C([H])C5)=CC(OC6C7=COC=C7)=O)(O8)C3OC8(C(C)CC)O4	

**58**	$C_{43}H_{48}O_{15}$	804.2993	805.3071	R = OAc. R1 = Bz. R2 = OAc. R3 = $CH_3CHCH_2CH_3$. R4 = H	OC12C3(OC(C)=O)C(OC(C4=CC=CC=C4)=O)C(C2)(C)C(C(OC(C)=O)C(OC)=O)C1(C)C56C(C(C7(C)C([H])C6)=CC(OC7C8=COC=C8)=O)(O9)C3OC9(C(C)CC)O5
**59**	$C_{41}H_{50}O_{16}$	798.3099	799.3177	R = OAc. R1 = Tg. R2 = OH. R3 = $CH_3CHCH_2CH_3$. R4 = OAc	OC12C3(O)C(OC(/C(C)=C/C)=O)C(C2)(C)C(C(OC(C)=O)C(OC)=O)C1(C)C45C(C(C6(C)C(OC(C)=O)C5)=CC(OC6C7=COC=C7)=O)(O8)C3OC8(C(C)CC)O4
**60**	$C_{43}H_{48}O_{16}$	820.2942	821.3021	R = OAc. R1 = Bz. R2 = OH. R3 = $CH_3CHCH_2CH_3$. R4 = OAc	OC12C3(O)C(OC(C4=CC=CC=C4)=O)C(C2)(C)C(C(OC(C)=O)C(OC)=O)C1(C)C56C(C(C7(C)C(OC(C)=O)C6)=CC(OC7C8=COC=C8)=O)(O9)C3OC9(C(C)CC)O5
**61**	$C_{41}H_{50}O_{15}$	782.3150	783.3228	R = H. R1 = Tg. R2 = OAc. R3 = $CH_3CHCH_2CH_3$. R4 = OAc	OC12C3(OC(C)=O)C(OC(/C(C)=C/C)=O)C(C2)(C)C(C([H])C(OC)=O)C1(C)C45C(C(C6(C)C(OC(C)=O)C5)=CC(OC6C7=COC=C7)=O)(O8)C3OC8(C(C)CC)O4
**62**	$C_{43}H_{48}O_{15}$	804.2993	805.3071	R = H. R1 = Bz. R2 = OAc. R3 = $CH_3CHCH_2CH_3$. R4 = OAc	OC12C3(OC(C)=O)C(OC(C4=CC=CC=C4)=O)C(C2)(C)C(C([H])C(OC)=O)C1(C)C56C(C(C7(C)C(OC(C)=O)C6)=CC(OC7C8=COC=C8)=O)(O9)C3OC9(C(C)CC)O5
**63**	$C_{43}H_{52}O_{17}$	840.3205	841.3283	R = OAc. R1 = Tg. R2 = OAc. R3 = $CH_3CHCH_2CH_3$. R4 = OAc	OC12C3(OC(C)=O)C(OC(/C(C)=C/C)=O)C(C2)(C)C(C(OC(C)=O)C(OC)=O)C1(C)C

					45C(C(C6(C)C(OC(C)=O)C5)=CC(OC6C7=COC=C7)=O)(O8)C3OC8(C(C)CC)O4	
**64**	$C_{45}H_{50}O_{17}$	862.3048	863.3126	R = OAc. R_1 = Bz. R_2 = OAc. R_3 = $CH_3CHCH_2CH_3$. R_4 = OAc	OC12C3(OC(C)=O)C(OC(C4=CC=CC=C4)=O)C(C2)(C)C(C(OC(C)=O)C(OC)=O)C1(C)C56C(C(C7(C)C(OC(C)=O)C6)=CC(OC7C8=COC=C8)=O)(O9)C3OC9(C(C)CC)O5	
**65**	$C_{37}H_{44}O_{12}$	680.2833	681.2911	R = H. R_1 = Tg. R_2 = OH. R_3 = (*E*)-$CH_3C{=}CHCH_3$. R_4 = H	OC12C3(O)C(OC(/C(C)=C/C)=O)C(C2)(C)C(C([H])C(OC)=O)C1(C)C45C(C(C6(C)C([H])C5)=CC(OC6C7=COC=C7)=O)(O8)C3OC8(/C(C)=C/C)O4	
**66**	$C_{39}H_{42}O_{12}$	702.2676	703.2755	R = H. R_1 = Bz. R_2 = OH. R_3 = (*E*)-$CH_3C{=}CHCH_3$. R_4 = H	OC12C3(O)C(OC(C4=CC=CC=C4)=O)C(C2)(C)C(C([H])C(OC)=O)C1(C)C56C(C(C7(C)C([H])C6)=CC(OC7C8=COC=C8)=O)(O9)C3OC9(/C(C)=C/C)O5	
**67**	$C_{39}H_{46}O_{14}$	738.2888	739.2966	R = OAc. R_1 = Tg. R_2 = OH. R_3 = (*E*)-$CH_3C{=}CHCH_3$. R_4 = H	OC12C3(O)C(OC(/C(C)=C/C)=O)C(C2)(C)C(C(OC(C)=O)C(OC)=O)C1(C)C45C(C(C6(C)C([H])C5)=CC(OC6C7=COC=C7)=O)(O8)C3OC8(/C(C)=C/C)O4	[2]
**68**	$C_{41}H_{44}O_{14}$	760.2731	761.2809	R = OAc. R_1 = Bz. R_2 = OH. R_3 = (*E*)-$CH_3C{=}CHCH_3$. R_4 = H	OC12C3(O)C(OC(C4=CC=CC=C4)=O)C(C2)(C)C(C(OC(C)=O)C(OC)=O)C1(C)C56C(C(C7(C)C([H])C6)=CC(OC7C8=COC=C8)=O)(O9)C3OC9(/C(C)=C/C)O5	

**69**	$C_{39}H_{46}O_{13}$	722.2938	723.3017	R = H. R_1 = Tg. R_2 = OAc. R_3 = (*E*)-CH_3C=$CHCH_3$. R_4 = H	OC12C3(OC(C)=O)C(OC(/C(C)=C/C)=O)C(C2)(C)C(C([H])C(OC)=O)C1(C)C45C(C(C6(C)C([H])C5)=CC(OC6C7=COC=C7)=O)(O8)C3OC8(/C(C)=C/C)O4	
**70**	$C_{41}H_{44}O_{13}$	744.2782	745.2860	R = H. R_1 = Bz. R_2 = OAc. R_3 = (*E*)-CH_3C=$CHCH_3$. R_4 = H	OC12C3(OC(C)=O)C(OC(C4=CC=CC=C4)=O)C(C2)(C)C(C([H])C(OC)=O)C1(C)C56C(C(C7(C)C([H])C6)=CC(OC7C8=COC=C8)=O)(O9)C3OC9(/C(C)=C/C)O5	
**71**	$C_{39}H_{46}O_{14}$	738.2888	739.2966	R = H. R_1 = Tg. R_2 = OH. R_3 = (*E*)-CH_3C=$CHCH_3$. R_4 = OAc	OC12C3(O)C(OC(/C(C)=C/C)=O)C(C2)(C)C(C([H])C(OC)=O)C1(C)C45C(C(C6(C)C(OC(C)=O)C5)=CC(OC6C7=COC=C7)=O)(O8)C3OC8(/C(C)=C/C)O4	[2]
**72**	$C_{41}H_{44}O_{14}$	760.2731	761.2809	R = H. R_1 = Bz. R_2 = OH. R_3 = (*E*)-CH_3C=$CHCH_3$. R_4 = OAc	OC12C3(O)C(OC(C4=CC=CC=C4)=O)C(C2)(C)C(C([H])C(OC)=O)C1(C)C56C(C(C7(C)C(OC(C)=O)C6)=CC(OC7C8=COC=C8)=O)(O9)C3OC9(/C(C)=C/C)O5	
**73**	$C_{41}H_{48}O_{15}$	780.2993	781.3071	R = OAc. R_1 = Tg. R_2 = OAc. R_3 = (*E*)-CH_3C=$CHCH_3$. R_4 = H	OC12C3(OC(C)=O)C(OC(/C(C)=C/C)=O)C(C2)(C)C(C(OC(C)=O)C(OC)=O)C1(C)C45C(C(C6(C)C([H])C5)=CC(OC6C7=COC=C7)=O)(O8)C3OC8(/C(C)=C/C)O4	
**74**	$C_{43}H_{46}O_{15}$	802.2837	803.2915	R = OAc. R_1 = Bz. R_2 = OAc. R_3 = (*E*)-CH_3C=$CHCH_3$. R_4 = H	OC12C3(OC(C)=O)C(OC(C4=CC=CC=C4)=O)C(C2)(C)C(C(OC(C)=O)C(OC)=O)C1(	

					C)C56C(C(C7(C)C([H])C6)=CC(OC7C8=COC=C8)=O)(O9)C3OC9(/C(C)=C/C)O5
**75**	$C_{41}H_{48}O_{16}$	796.2942	797.3021	R = OAc. R_1 = Tg. R_2 = OH. R_3 = (*E*)-CH_3C=$CHCH_3$. R_4 = OAc	OC12C3(O)C(OC(/C(C)=C/C)=O)C(C2)(C)C(C(OC(C)=O)C(OC)=O)C1(C)C45C(C(C6(C)C(OC(C)=O)C5)=CC(OC6C7=COC=C7)=O)(O8)C3OC8(/C(C)=C/C)O4
**76**	$C_{43}H_{46}O_{16}$	818.2786	819.2864	R = OAc. R_1 = Bz. R_2 = OH. R_3 = (*E*)-CH_3C=$CHCH_3$. R_4 = OAc	OC12C3(O)C(OC(C4=CC=CC=C4)=O)C(C2)(C)C(C(OC(C)=O)C(OC)=O)C1(C)C56C(C(C7(C)C(OC(C)=O)C6)=CC(OC7C8=COC=C8)=O)(O9)C3OC9(/C(C)=C/C)O5
**77**	$C_{41}H_{48}O_{15}$	780.2993	781.3071	R = H. R_1 = Tg. R_2 = OAc. R_3 = (*E*)-CH_3C=$CHCH_3$. R_4 = OAc	OC12C3(OC(C)=O)C(OC(/C(C)=C/C)=O)C(C2)(C)C(C([H])C(OC)=O)C1(C)C45C(C(C6(C)C(OC(C)=O)C5)=CC(OC6C7=COC=C7)=O)(O8)C3OC8(/C(C)=C/C)O4
**78**	$C_{43}H_{46}O_{15}$	802.2837	803.2915	R = H. R_1 = Bz. R_2 = OAc. R_3 = (*E*)-CH_3C=$CHCH_3$. R_4 = OAc	OC12C3(OC(C)=O)C(OC(C4=CC=CC=C4)=O)C(C2)(C)C(C([H])C(OC)=O)C1(C)C56C(C(C7(C)C(OC(C)=O)C6)=CC(OC7C8=COC=C8)=O)(O9)C3OC9(/C(C)=C/C)O5
**79**	$C_{43}H_{50}O_{17}$	838.3048	839.3126	R = OAc. R_1 = Tg. R_2 = OAc. R_3 = (*E*)-CH_3C=$CHCH_3$. R_4 = OAc	OC12C3(OC(C)=O)C(OC(/C(C)=C/C)=O)C(C2)(C)C(C(OC(C)=O)C(OC)=O)C1(C)C45C(C(C6(C)C(OC(C)=O)C5)=CC(OC6C7=COC=C7)=O)(O8)C3OC8(/C(C)=C/C)O4

**80**	$C_{45}H_{48}O_{17}$	860.2892	861.2970	R = OAc. R1 = Bz. R2 = OAc. R3 = (*E*)-CH3C=CHCH3. R4 = OAc	OC12C3(OC(C)=O)C(OC(C4=CC=CC=C4)=O)C(C2)(C)C(C(OC(C)=O)C(OC)=O)C1(C)C56C(C(C7(C)C(OC(C)=O)C6)=CC(OC7C8=COC=C8)=O)(O9)C3OC9(/C(C)=C/C)O5	
**81**	$C_{34}H_{40}O_{13}$	656.2469	657.2547	R = H. R1 = Tg. R2 = OH. R3 = CH3. R4 = OH	OC12C3(O)C(OC(/C(C)=C/C)=O)C(C2)(C)C(C([H])C(OC)=O)C1(C)C45C(C(C6(C)C(O)C5)=CC(OC6C7=COC=C7)=O)(O8)C3OC8(C)O4	[9,1]
**82**	$C_{36}H_{38}O_{13}$	678.2312	679.2391	R = H. R1 = Bz. R2 = OH. R3 = CH3. R4 = OH	OC12C3(O)C(OC(C4=CC=CC=C4)=O)C(C2)(C)C(C([H])C(OC)=O)C1(C)C56C(C(C7(C)C(O)C6)=CC(OC7C8=COC=C8)=O)(O9)C3OC9(C)O5	
**83**	$C_{36}H_{42}O_{15}$	714.2524	715.2602	R = OAc. R1 = Tg. R2 = OH. R3 = CH3. R4 = OH	OC12C3(O)C(OC(/C(C)=C/C)=O)C(C2)(C)C(C(OC(C)=O)C(OC)=O)C1(C)C45C(C(C6(C)C(O)C5)=CC(OC6C7=COC=C7)=O)(O8)C3OC8(C)O4	
**84**	$C_{38}H_{40}O_{15}$	736.2367	737.2445	R = OAc. R1 = Bz. R2 = OH. R3 = CH3. R4 = OH	OC12C3(O)C(OC(C4=CC=CC=C4)=O)C(C2)(C)C(C(OC(C)=O)C(OC)=O)C1(C)C56C(C(C7(C)C(O)C6)=CC(OC7C8=COC=C8)=O)(O9)C3OC9(C)O5	
**85**	$C_{36}H_{42}O_{14}$	698.2575	699.2653	R = H. R1 = Tg. R2 = OAc. R3 = CH3. R4 = OH	OC12C3(OC(C)=O)C(OC(/C(C)=C/C)=O)C(C2)(C)C(C([H])C(OC)=O)C1(C)C45C(C	

					(C6(C)C(O)C5)=CC(OC6C7=COC=C7)=O)(O8)C3OC8(C)O4
**86**	$C_{38}H_{40}O_{14}$	720.2418	721.2496	R = H. R1 = Bz. R2 = OAc. R3 = CH3. R4 = OH	OC12C3(OC(C)=O)C(OC(C4=CC=CC=C4)=O)C(C2)(C)C(C([H])C(OC)=O)C1(C)C56C(C(C7(C)C(O)C6)=CC(OC7C8=COC=C8)=O)(O9)C3OC9(C)O5
**87**	$C_{38}H_{44}O_{16}$	756.2629	757.2708	R = OAc. R1 = Tg. R2 = OAc. R3 = CH3. R4 = OH	OC12C3(OC(C)=O)C(OC(/C(C)=C/C)=O)C(C2)(C)C(C(OC(C)=O)C(OC)=O)C1(C)C45C(C(C6(C)C(O)C5)=CC(OC6C7=COC=C7)=O)(O8)C3OC8(C)O4
**88**	$C_{40}H_{42}O_{16}$	778.2473	779.2551	R = OAc. R1 = Bz. R2 = OAc. R3 = CH3. R4 = OH	OC12C3(OC(C)=O)C(OC(C4=CC=CC=C4)=O)C(C2)(C)C(C(OC(C)=O)C(OC)=O)C1(C)C56C(C(C7(C)C(O)C6)=CC(OC7C8=COC=C8)=O)(O9)C3OC9(C)O5
**89**	$C_{35}H_{42}O_{13}$	670.2625	671.2704	R = H. R1 = Tg. R2 = OH. R3 = CH2CH3. R4 = OH	OC12C3(O)C(OC(/C(C)=C/C)=O)C(C2)(C)C(C([H])C(OC)=O)C1(C)C45C(C(C6(C)C(O)C5)=CC(OC6C7=COC=C7)=O)(O8)C3OC8(CC)O4
**90**	$C_{37}H_{40}O_{13}$	692.2469	693.2547	R = H. R1 = Bz. R2 = OH. R3 = CH2CH3. R4 = OH	OC12C3(O)C(OC(C4=CC=CC=C4)=O)C(C2)(C)C(C([H])C(OC)=O)C1(C)C56C(C(C7(C)C(O)C6)=CC(OC7C8=COC=C8)=O)(O9)C3OC9(CC)O5

**91**	$C_{37}H_{44}O_{15}$	728.2680	729.2758	R = OAc. R_1 = Tg. R_2 = OH. R_3 = CH_2CH_3. R_4 = OH	OC12C3(O)C(OC(/C(C)=C/C)=O)C(C2)(C)C(C(OC(C)=O)C(OC)=O)C1(C)C45C(C(C6(C)C(O)C5)=CC(OC6C7=COC=C7)=O)(O8)C3OC8(CC)O4	
**92**	$C_{39}H_{42}O_{15}$	750.2524	751.2602	R = OAc. R_1 = Bz. R_2 = OH. R_3 = CH_2CH_3. R_4 = OH	OC12C3(O)C(OC(C4=CC=CC=C4)=O)C(C2)(C)C(C(OC(C)=O)C(OC)=O)C1(C)C56C(C(C7(C)C(O)C6)=CC(OC7C8=COC=C8)=O)(O9)C3OC9(CC)O5	
**93**	$C_{37}H_{44}O_{14}$	712.2731	713.2809	R = H. R_1 = Tg. R_2 = OAc. R_3 = CH_2CH_3. R_4 = OH	OC12C3(OC(C)=O)C(OC(/C(C)=C/C)=O)C(C2)(C)C(C([H])C(OC)=O)C1(C)C45C(C(C6(C)C(O)C5)=CC(OC6C7=COC=C7)=O)(O8)C3OC8(CC)O4	[5]
**94**	$C_{39}H_{42}O_{14}$	734.2575	735.2653	R = H. R_1 = Bz. R_2 = OAc. R_3 = CH_2CH_3. R_4 = OH	OC12C3(OC(C)=O)C(OC(C4=CC=CC=C4)=O)C(C2)(C)C(C([H])C(OC)=O)C1(C)C56C(C(C7(C)C(O)C6)=CC(OC7C8=COC=C8)=O)(O9)C3OC9(CC)O5	
**95**	$C_{39}H_{46}O_{16}$	770.2786	771.2864	R = OAc. R_1 = Tg. R_2 = OAc. R_3 = CH_2CH_3. R_4 = OH	OC12C3(OC(C)=O)C(OC(/C(C)=C/C)=O)C(C2)(C)C(C(OC(C)=O)C(OC)=O)C1(C)C45C(C(C6(C)C(O)C5)=CC(OC6C7=COC=C7)=O)(O8)C3OC8(CC)O4	
**96**	$C_{41}H_{44}O_{16}$	792.2629	793.2708	R = OAc. R_1 = Bz. R_2 = OAc. R_3 = CH_2CH_3. R_4 = OH	OC12C3(OC(C)=O)C(OC(C4=CC=CC=C4)=O)C(C2)(C)C(C(OC(C)=O)C(OC)=O)C1(	

					C)C56C(C(C7(C)C(O)C6)=CC(OC7C8=COC=C8)=O)(O9)C3OC9(CC)O5
**97**	$C_{36}H_{44}O_{13}$	684.2782	685.2860	R = H. R_1 = Tg. R_2 = OH. R_3 = $CH(CH_3)_2$. R_4 = OH	OC12C3(O)C(OC(/C(C)=C/C)=O)C(C2)(C)C(C([H])C(OC)=O)C1(C)C45C(C(C6(C)C(O)C5)=CC(OC6C7=COC=C7)=O)(O8)C3OC8(C(C)C)O4
**98**	$C_{38}H_{42}O_{13}$	706.2625	707.2704	R = H. R_1 = Bz. R_2 = OH. R_3 = $CH(CH_3)_2$. R_4 = OH	OC12C3(O)C(OC(C4=CC=CC=C4)=O)C(C2)(C)C(C([H])C(OC)=O)C1(C)C56C(C(C7(C)C(O)C6)=CC(OC7C8=COC=C8)=O)(O9)C3OC9(C(C)C)O5
**99**	$C_{38}H_{46}O_{15}$	742.2837	743.2915	R = OAc. R_1 = Tg. R_2 = OH. R_3 = $CH(CH_3)_2$. R_4 = OH	OC12C3(O)C(OC(/C(C)=C/C)=O)C(C2)(C)C(C(OC(C)=O)C(OC)=O)C1(C)C45C(C(C6(C)C(O)C5)=CC(OC6C7=COC=C7)=O)(O8)C3OC8(C(C)C)O4
**100**	$C_{40}H_{44}O_{15}$	764.2680	765.2758	R = OAc. R_1 = Bz. R_2 = OH. R_3 = $CH(CH_3)_2$. R_4 = OH	OC12C3(O)C(OC(C4=CC=CC=C4)=O)C(C2)(C)C(C(OC(C)=O)C(OC)=O)C1(C)C56C(C(C7(C)C(O)C6)=CC(OC7C8=COC=C8)=O)(O9)C3OC9(C(C)C)O5
**101**	$C_{38}H_{46}O_{14}$	726.2888	727.2966	R = H. R_1 = Tg. R_2 = OAc. R_3 = $CH(CH_3)_2$. R_4 = OH	OC12C3(OC(C)=O)C(OC(/C(C)=C/C)=O)C(C2)(C)C(C([H])C(OC)=O)C1(C)C45C(C(C6(C)C(O)C5)=CC(OC6C7=COC=C7)=O)(O8)C3OC8(C(C)C)O4

**102**	$C_{40}H_{44}O_{14}$	748.2731	749.2809	R = H. R_1 = Bz. R_2 = OAc. R_3 = $CH(CH_3)_2$. R_4 = OH	OC12C3(OC(C)=O)C(OC(C4=CC=CC=C4)=O)C(C2)(C)C(C([H])C(OC)=O)C1(C)C56C(C(C7(C)C(O)C6)=CC(OC7C8=COC=C8)=O)(O9)C3OC9(C(C)C)O5
**103**	$C_{40}H_{48}O_{16}$	784.2942	785.3021	R = OAc. R_1 = Tg. R_2 = OAc. R_3 = $CH(CH_3)_2$. R_4 = OH	OC12C3(OC(C)=O)C(OC(/C(C)=C/C)=O)C(C2)(C)C(C(OC(C)=O)C(OC)=O)C1(C)C45C(C(C6(C)C(O)C5)=CC(OC6C7=COC=C7)=O)(O8)C3OC8(C(C)C)O4
**104**	$C_{42}H_{46}O_{16}$	806.2786	807.2864	R = OAc. R_1 = Bz. R_2 = OAc. R_3 = $CH(CH_3)_2$. R_4 = OH	OC12C3(OC(C)=O)C(OC(C4=CC=CC=C4)=O)C(C2)(C)C(C(OC(C)=O)C(OC)=O)C1(C)C56C(C(C7(C)C(O)C6)=CC(OC7C8=COC=C8)=O)(O9)C3OC9(C(C)C)O5
**105**	$C_{37}H_{46}O_{13}$	698.2938	699.3017	R = H. R_1 = Tg. R_2 = OH. R_3 = $CH_3CHCH_2CH_3$. R_4 = OH	OC12C3(O)C(OC(/C(C)=C/C)=O)C(C2)(C)C(C([H])C(OC)=O)C1(C)C45C(C(C6(C)C(O)C5)=CC(OC6C7=COC=C7)=O)(O8)C3OC8(C(C)CC)O4
**106**	$C_{39}H_{44}O_{13}$	720.2782	721.2860	R = H. R_1 = Bz. R_2 = OH. R_3 = $CH_3CHCH_2CH_3$. R_4 = OH	OC12C3(O)C(OC(C4=CC=CC=C4)=O)C(C2)(C)C(C([H])C(OC)=O)C1(C)C56C(C(C7(C)C(O)C6)=CC(OC7C8=COC=C8)=O)(O9)C3OC9(C(C)CC)O5
**107**	$C_{39}H_{48}O_{15}$	756.2993	757.3071	R = OAc. R_1 = Tg. R_2 = OH. R_3 = $CH_3CHCH_2CH_3$. R_4 = OH	OC12C3(O)C(OC(/C(C)=C/C)=O)C(C2)(C)C(C(OC(C)=O)C(OC)=O)C1(C)C45C(C(C6

					(C)C(O)C5)=CC(OC6C7=COC=C7)=O)(O8)C3OC8(C(C)CC)O4
**108**	$C_{41}H_{46}O_{15}$	778.2837	779.2915	R = OAc. R_1 = Bz. R_2 = OH. R_3 = $CH_3CHCH_2CH_3$. R_4 = OH	OC12C3(O)C(OC(C4=CC=CC=C4)=O)C(C2)(C)C(C(OC(C)=O)C(OC)=O)C1(C)C56C(C(C7(C)C(O)C6)=CC(OC7C8=COC=C8)=O)(O9)C3OC9(C(C)CC)O5
**109**	$C_{39}H_{48}O_{14}$	740.3044	741.3122	R = H. R_1 = Tg. R_2 = OAc. R_3 = $CH_3CHCH_2CH_3$. R_4 = OH	OC12C3(OC(C)=O)C(OC(/C(C)=C/C)=O)C(C2)(C)C(C([H])C(OC)=O)C1(C)C45C(C(C6(C)C(O)C5)=CC(OC6C7=COC=C7)=O)(O8)C3OC8(C(C)CC)O4
**110**	$C_{41}H_{46}O_{14}$	762.2888	763.2966	R = H. R_1 = Bz. R_2 = OAc. R_3 = $CH_3CHCH_2CH_3$. R_4 = OH	OC12C3(OC(C)=O)C(OC(C4=CC=CC=C4)=O)C(C2)(C)C(C([H])C(OC)=O)C1(C)C56C(C(C7(C)C(O)C6)=CC(OC7C8=COC=C8)=O)(O9)C3OC9(C(C)CC)O5
**111**	$C_{41}H_{50}O_{16}$	798.3099	799.3177	R = OAc. R_1 = Tg. R_2 = OAc. R_3 = $CH_3CHCH_2CH_3$. R_4 = OH	OC12C3(OC(C)=O)C(OC(/C(C)=C/C)=O)C(C2)(C)C(C(OC(C)=O)C(OC)=O)C1(C)C45C(C(C6(C)C(O)C5)=CC(OC6C7=COC=C7)=O)(O8)C3OC8(C(C)CC)O4
**112**	$C_{43}H_{48}O_{16}$	820.2942	821.3021	R = OAc. R_1 = Bz. R_2 = OAc. R_3 = $CH_3CHCH_2CH_3$. R_4 = OH	OC12C3(OC(C)=O)C(OC(C4=CC=CC=C4)=O)C(C2)(C)C(C(OC(C)=O)C(OC)=O)C1(C)C56C(C(C7(C)C(O)C6)=CC(OC7C8=COC=C8)=O)(O9)C3OC9(C(C)CC)O5

**113**	$C_{37}H_{44}O_{13}$	696.2782	697.2860	R = H. R_1 = Tg. R_2 = OH. R_3 = (*E*)-$CH_3C{=}CHCH_3$. R_4 = OH	OC12C3(O)C(OC(/C(C)=C/C)=O)C(C2)(C)C(C([H])C(OC)=O)C1(C)C45C(C(C6(C)C(O)C5)=CC(OC6C7=COC=C7)=O)(O8)C3OC8(/C(C)=C/C)O4
**114**	$C_{39}H_{42}O_{13}$	718.2625	719.2704	R = H. R_1 = Bz. R_2 = OH. R_3 = (*E*)-$CH_3C{=}CHCH_3$. R_4 = OH	OC12C3(O)C(OC(C4=CC=CC=C4)=O)C(C2)(C)C(C([H])C(OC)=O)C1(C)C56C(C(C7(C)C(O)C6)=CC(OC7C8=COC=C8)=O)(O9)C3OC9(/C(C)=C/C)O5
**115**	$C_{39}H_{46}O_{15}$	754.2837	755.2915	R = OAc. R_1 = Tg. R_2 = OH. R_3 = (*E*)-$CH_3C{=}CHCH_3$. R_4 = OH	OC12C3(O)C(OC(/C(C)=C/C)=O)C(C2)(C)C(C(OC(C)=O)C(OC)=O)C1(C)C45C(C(C6(C)C(O)C5)=CC(OC6C7=COC=C7)=O)(O8)C3OC8(/C(C)=C/C)O4
**116**	$C_{41}H_{44}O_{15}$	776.2680	777.2758	R = OAc. R_1 = Bz. R_2 = OH. R_3 = (*E*)-$CH_3C{=}CHCH_3$. R_4 = OH	OC12C3(O)C(OC(C4=CC=CC=C4)=O)C(C2)(C)C(C(OC(C)=O)C(OC)=O)C1(C)C56C(C(C7(C)C(O)C6)=CC(OC7C8=COC=C8)=O)(O9)C3OC9(/C(C)=C/C)O5
**117**	$C_{39}H_{46}O_{14}$	738.2888	739.2966	R = H. R_1 = Tg. R_2 = OAc. R_3 = (*E*)-$CH_3C{=}CHCH_3$. R_4 = OH	OC12C3(OC(C)=O)C(OC(/C(C)=C/C)=O)C(C2)(C)C(C([H])C(OC)=O)C1(C)C45C(C(C6(C)C(O)C5)=CC(OC6C7=COC=C7)=O)(O8)C3OC8(/C(C)=C/C)O4
**118**	$C_{41}H_{44}O_{14}$	760.2731	761.2809	R = H. R_1 = Bz. R_2 = OAc. R_3 = (*E*)-$CH_3C{=}CHCH_3$. R_4 = OH	OC12C3(OC(C)=O)C(OC(C4=CC=CC=C4)=O)C(C2)(C)C(C([H])C(OC)=O)C1(C)C56

					C(C(C7(C)C(O)C6)=CC(OC7C8=COC=C8)=O)(O9)C3OC9(/C(C)=C/C)O5
**119**	$C_{41}H_{48}O_{16}$	796.2942	797.3021	R = OAc. R_1 = Tg. R_2 = OAc. R_3 = (*E*)-CH_3C=$CHCH_3$. R_4 = OH	OC12C3(OC(C)=O)C(OC(/C(C)=C/C)=O)C(C2)(C)C(C(OC(C)=O)C(OC)=O)C1(C)C45C(C(C6(C)C(O)C5)=CC(OC6C7=COC=C7)=O)(O8)C3OC8(/C(C)=C/C)O4
**120**	$C_{43}H_{46}O_{16}$	818.2786	819.2864	R = OAc. R_1 = Bz. R_2 = OAc. R_3 = (*E*)-CH_3C=$CHCH_3$. R_4 = OH	OC12C3(OC(C)=O)C(OC(C4=CC=CC=C4)=O)C(C2)(C)C(C(OC(C)=O)C(OC)=O)C1(C)C56C(C(C7(C)C(O)C6)=CC(OC7C8=COC=C8)=O)(O9)C3OC9(/C(C)=C/C)O5
**121**	$C_{34}H_{40}O_{11}$	624.2571	625.2649	R = H. R_1 = Tg. R_2 = H. R_3 = CH_3. R_4 = H	OC12C3([H])C(OC(/C(C)=C/C)=O)C(C2)(C)C(C([H])C(OC)=O)C1(C)C45C(C(C6(C)C([H])C5)=CC(OC6C7=COC=C7)=O)(O8)C3OC8(C)O4
**122**	$C_{36}H_{38}O_{11}$	646.2424	647.2492	R = H. R_1 = Bz. R_2 = H. R_3 = CH_3. R_4 = H	OC12C3([H])C(OC(C4=CC=CC=C4)=O)C(C2)(C)C(C([H])C(OC)=O)C1(C)C56C(C(C7(C)C([H])C6)=CC(OC7C8=COC=C8)=O)(O9)C3OC9(C)O5
**123**	$C_{36}H_{42}O_{13}$	682.2625	683.2704	R = OAc. R_1 = Tg. R_2 = H. R_3 = CH_3. R_4 = H	OC12C3([H])C(OC(/C(C)=C/C)=O)C(C2)(C)C(C(OC(C)=O)C(OC)=O)C1(C)C45C(C(C6(C)C([H])C5)=CC(OC6C7=COC=C7)=O)(O8)C3OC8(C)O4

**124**	$C_{38}H_{40}O_{13}$	704.2469	705.2547	R = OAc. R_1 = Bz. R_2 = H. R_3 = CH_3. R_4 = H	OC12C3([H])C(OC(C4=CC=CC=C4)=O)C(C2)(C)C(C(OC(C)=O)C(OC)=O)C1(C)C56C(C(C7(C)C([H])C6)=CC(OC7C8=COC=C8)=O)(O9)C3OC9(C)O5
**125**	$C_{36}H_{42}O_{13}$	682.2625	683.2704	R = H. R_1 = Tg. R_2 = H. R_3 = CH_3. R_4 = OAc	OC12C3([H])C(OC(/C(C)=C/C)=O)C(C2)(C)C(C([H])C(OC)=O)C1(C)C45C(C(C6(C)C(OC(C)=O)C5)=CC(OC6C7=COC=C7)=O)(O8)C3OC8(C)O4
**126**	$C_{38}H_{40}O_{13}$	704.2469	705.2547	R = H. R_1 = Bz. R_2 = H. R_3 = CH_3. R_4 = OAc	OC12C3([H])C(OC(C4=CC=CC=C4)=O)C(C2)(C)C(C([H])C(OC)=O)C1(C)C56C(C(C7(C)C(OC(C)=O)C6)=CC(OC7C8=COC=C8)=O)(O9)C3OC9(C)O5
**127**	$C_{38}H_{44}O_{15}$	740.2680	741.2758	R = OAc. R_1 = Tg. R_2 = H. R_3 = CH_3. R_4 = OAc	OC12C3([H])C(OC(/C(C)=C/C)=O)C(C2)(C)C(C(OC(C)=O)C(OC)=O)C1(C)C45C(C(C6(C)C(OC(C)=O)C5)=CC(OC6C7=COC=C7)=O)(O8)C3OC8(C)O4
**128**	$C_{40}H_{42}O_{15}$	762.2524	763.2602	R = OAc. R_1 = Bz. R_2 = H. R_3 = CH_3. R_4 = OAc	OC12C3([H])C(OC(C4=CC=CC=C4)=O)C(C2)(C)C(C(OC(C)=O)C(OC)=O)C1(C)C56C(C(C7(C)C(OC(C)=O)C6)=CC(OC7C8=COC=C8)=O)(O9)C3OC9(C)O5
**129**	$C_{34}H_{40}O_{12}$	640.2520	641.2598	R = H. R_1 = Tg. R_2 = H. R_3 = CH_3. R_4 = OH	OC12C3([H])C(OC(/C(C)=C/C)=O)C(C2)(C)C(C([H])C(OC)=O)C1(C)C45C(C(C6(C)

					C(O)C5)=CC(OC6C7=COC=C7)=O)(O8)C3OC8(C)O4
**130**	$C_{36}H_{38}O_{12}$	662.2363	663.2442	R = H. R_1 = Bz. R_2 = H. R_3 = CH_3. R_4 = OH	OC12C3([H])C(OC(C4=CC=CC=C4)=O)C(C2)(C)C(C([H])C(OC)=O)C1(C)C56C(C(C7(C)C(O)C6)=CC(OC7C8=COC=C8)=O)(O9)C3OC9(C)O5
**131**	$C_{36}H_{42}O_{14}$	698.2575	699.2653	R = OAc. R_1 = Tg. R_2 = H. R_3 = CH_3. R_4 = OH	OC12C3([H])C(OC(/C(C)=C/C)=O)C(C2)(C)C(C(OC(C)=O)C(OC)=O)C1(C)C45C(C(C6(C)C(O)C5)=CC(OC6C7=COC=C7)=O)(O8)C3OC8(C)O4
**132**	$C_{38}H_{40}O_{14}$	720.2418	721.2496	R = OAc. R_1 = Bz. R_2 = H. R_3 = CH_3. R_4 = OH	OC12C3([H])C(OC(C4=CC=CC=C4)=O)C(C2)(C)C(C(OC(C)=O)C(OC)=O)C1(C)C56C(C(C7(C)C(O)C6)=CC(OC7C8=COC=C8)=O)(O9)C3OC9(C)O5
**133**	$C_{35}H_{42}O_{11}$	638.2727	639.2809	R = H. R_1 = Tg. R_2 = H. R_3 = CH_2CH_3. R_4 = H	OC12C3([H])C(OC(/C(C)=C/C)=O)C(C2)(C)C(C([H])C(OC)=O)C1(C)C45C(C(C6(C)C([H])C5)=CC(OC6C7=COC=C7)=O)(O8)C3OC8(CC)O4
**134**	$C_{37}H_{40}O_{11}$	660.2571	661.2649	R = H. R_1 = Bz. R_2 = H. R_3 = CH_2CH_3. R_4 = H	OC12C3([H])C(OC(C4=CC=CC=C4)=O)C(C2)(C)C(C([H])C(OC)=O)C1(C)C56C(C(C7(C)C([H])C6)=CC(OC7C8=COC=C8)=O)(O9)C3OC9(CC)O5

**135**	$C_{37}H_{44}O_{13}$	696.2782	697.2860	R = OAc. R_1 = Tg. R_2 = H. R_3 = CH_2CH_3. R_4 = H	OC12C3([H])C(OC(/C(C)=C/C)=O)C(C2)(C)C(C(OC(C)=O)C(OC)=O)C1(C)C45C(C(C6(C)C([H])C5)=CC(OC6C7=COC=C7)=O)(O8)C3OC8(CC)O4
**136**	$C_{39}H_{42}O_{13}$	718.2625	719.2704	R = OAc. R_1 = Bz. R_2 = H. R_3 = CH_2CH_3. R_4 = H	OC12C3([H])C(OC(C4=CC=CC=C4)=O)C(C2)(C)C(C(OC(C)=O)C(OC)=O)C1(C)C56C(C(C7(C)C([H])C6)=CC(OC7C8=COC=C8)=O)(O9)C3OC9(CC)O5
**137**	$C_{37}H_{44}O_{13}$	696.2782	697.2860	R = H. R_1 = Tg. R_2 = H. R_3 = CH_2CH_3. R_4 = OAc	OC12C3([H])C(OC(/C(C)=C/C)=O)C(C2)(C)C(C([H])C(OC)=O)C1(C)C45C(C(C6(C)C(OC(C)=O)C5)=CC(OC6C7=COC=C7)=O)(O8)C3OC8(CC)O4
**138**	$C_{39}H_{42}O_{13}$	718.2625	719.2704	R = H. R_1 = Bz. R_2 = H. R_3 = CH_2CH_3. R_4 = OAc	OC12C3([H])C(OC(C4=CC=CC=C4)=O)C(C2)(C)C(C([H])C(OC)=O)C1(C)C56C(C(C7(C)C(OC(C)=O)C6)=CC(OC7C8=COC=C8)=O)(O9)C3OC9(CC)O5
**139**	$C_{39}H_{46}O_{15}$	754.2837	755.2915	R = OAc. R_1 = Tg. R_2 = H. R_3 = CH_2CH_3. R_4 = OAc	OC12C3([H])C(OC(/C(C)=C/C)=O)C(C2)(C)C(C(OC(C)=O)C(OC)=O)C1(C)C45C(C(C6(C)C(OC(C)=O)C5)=CC(OC6C7=COC=C7)=O)(O8)C3OC8(CC)O4
**140**	$C_{41}H_{44}O_{15}$	776.2680	777.2758	R = OAc. R_1 = Bz. R_2 = H. R_3 = CH_2CH_3. R_4 = OAc	OC12C3([H])C(OC(C4=CC=CC=C4)=O)C(C2)(C)C(C(OC(C)=O)C(OC)=O)C1(C)C56

					C(C(C7(C)C(OC(C)=O)C6)=CC(OC7C8=COC=C8)=O)(O9)C3OC9(CC)O5
**141**	$C_{35}H_{42}O_{12}$	654.2676	655.2755	R = H. R_1 = Tg. R_2 = H. R_3 = CH_2CH_3. R_4 = OH	OC12C3([H])C(OC(/C(C)=C/C)=O)C(C2)(C)C(C([H])C(OC)=O)C1(C)C45C(C(C6(C)C(O)C5)=CC(OC6C7=COC=C7)=O)(O8)C3OC8(CC)O4
**142**	$C_{37}H_{40}O_{12}$	676.2520	677.2598	R = H. R_1 = Bz. R_2 = H. R_3 = CH_2CH_3. R_4 = OH	OC12C3([H])C(OC(C4=CC=CC=C4)=O)C(C2)(C)C(C([H])C(OC)=O)C1(C)C56C(C(C7(C)C(O)C6)=CC(OC7C8=COC=C8)=O)(O9)C3OC9(CC)O5
**143**	$C_{37}H_{44}O_{14}$	712.2731	713.2809	R = OAc. R_1 = Tg. R_2 = H. R_3 = CH_2CH_3. R_4 = OH	OC12C3([H])C(OC(/C(C)=C/C)=O)C(C2)(C)C(C(OC(C)=O)C(OC)=O)C1(C)C45C(C(C6(C)C(O)C5)=CC(OC6C7=COC=C7)=O)(O8)C3OC8(CC)O4
**144**	$C_{39}H_{42}O_{14}$	734.2575	735.2653	R = OAc. R_1 = Bz. R_2 = H. R_3 = CH_2CH_3. R_4 = OH	OC12C3([H])C(OC(C4=CC=CC=C4)=O)C(C2)(C)C(C(OC(C)=O)C(OC)=O)C1(C)C56C(C(C7(C)C(O)C6)=CC(OC7C8=COC=C8)=O)(O9)C3OC9(CC)O5
**145**	$C_{36}H_{44}O_{11}$	652.2884	653.2962	R = H. R_1 = Tg. R_2 = H. R_3 = $CH(CH_3)_2$. R_4 = H	OC12C3([H])C(OC(/C(C)=C/C)=O)C(C2)(C)C(C([H])C(OC)=O)C1(C)C45C(C(C6(C)C([H])C5)=CC(OC6C7=COC=C7)=O)(O8)C3OC8(C(C)C)O4

**146**	$C_{38}H_{42}O_{11}$	674.2727	675.2805	R = H. R_1 = Bz. R_2 = H. R_3 = $CH(CH_3)_2$. R_4 = H	OC12C3([H])C(OC(C4=CC=CC=C4)=O)C(C2)(C)C(C([H])C(OC)=O)C1(C)C56C(C(C7(C)C([H])C6)=CC(OC7C8=COC=C8)=O)(O9)C3OC9(C(C)C)O5
**147**	$C_{38}H_{46}O_{13}$	710.2938	711.3017	R = OAc. R_1 = Tg. R_2 = H. R_3 = $CH(CH_3)_2$. R_4 = H	OC12C3([H])C(OC(/C(C)=C/C)=O)C(C2)(C)C(C(OC(C)=O)C(OC)=O)C1(C)C45C(C(C6(C)C([H])C5)=CC(OC6C7=COC=C7)=O)(O8)C3OC8(C(C)C)O4
**148**	$C_{40}H_{44}O_{13}$	732.2782	733.2860	R = OAc. R_1 = Bz. R_2 = H. R_3 = $CH(CH_3)_2$. R_4 = H	OC12C3([H])C(OC(C4=CC=CC=C4)=O)C(C2)(C)C(C(OC(C)=O)C(OC)=O)C1(C)C56C(C(C7(C)C([H])C6)=CC(OC7C8=COC=C8)=O)(O9)C3OC9(C(C)C)O5
**149**	$C_{38}H_{46}O_{13}$	710.2938	711.3017	R = H. R_1 = Tg. R_2 = H. R_3 = $CH(CH_3)_2$. R_4 = OAc	OC12C3([H])C(OC(/C(C)=C/C)=O)C(C2)(C)C(C([H])C(OC)=O)C1(C)C45C(C(C6(C)C(OC(C)=O)C5)=CC(OC6C7=COC=C7)=O)(O8)C3OC8(C(C)C)O4
**150**	$C_{40}H_{44}O_{13}$	732.2782	733.2860	R = H. R_1 = Bz. R_2 = H. R_3 = $CH(CH_3)_2$. R_4 = OAc	OC12C3([H])C(OC(C4=CC=CC=C4)=O)C(C2)(C)C(C([H])C(OC)=O)C1(C)C56C(C(C7(C)C(OC(C)=O)C6)=CC(OC7C8=COC=C8)=O)(O9)C3OC9(C(C)C)O5
**151**	$C_{40}H_{48}O_{15}$	768.2998	769.3071	R = OAc. R_1 = Tg. R_2 = H. R_3 = $CH(CH_3)_2$. R_4 = OAc	OC12C3([H])C(OC(/C(C)=C/C)=O)C(C2)(C)C(C(OC(C)=O)C(OC)=O)C1(C)C45C(C(

					C6(C)C(OC(C)=O)C5)=CC(OC6C7=COC=C7)=O)(O8)C3OC8(C(C)C)O4
**152**	$C_{42}H_{46}O_{15}$	790.2837	791.2915	R = OAc. R_1 = Bz. R_2 = H. R_3 = $CH(CH_3)_2$. R_4 = OAc	OC12C3([H])C(OC(C4=CC=CC=C4)=O)C(C2)(C)C(C(OC(C)=O)C(OC)=O)C1(C)C56C(C(C7(C)C(OC(C)=O)C6)=CC(OC7C8=COC=C8)=O)(O9)C3OC9(C(C)C)O5
**153**	$C_{36}H_{44}O_{12}$	668.2833	669.2911	R = H. R_1 = Tg. R_2 = H. R_3 = $CH(CH_3)_2$. R_4 = OH	OC12C3([H])C(OC(/C(C)=C/C)=O)C(C2)(C)C(C([H])C(OC)=O)C1(C)C45C(C(C6(C)C(O)C5)=CC(OC6C7=COC=C7)=O)(O8)C3OC8(C(C)C)O4
**154**	$C_{38}H_{42}O_{12}$	690.2676	691.2755	R = H. R_1 = Bz. R_2 = H. R_3 = $CH(CH_3)_2$. R_4 = OH	OC12C3([H])C(OC(C4=CC=CC=C4)=O)C(C2)(C)C(C([H])C(OC)=O)C1(C)C56C(C(C7(C)C(O)C6)=CC(OC7C8=COC=C8)=O)(O9)C3OC9(C(C)C)O5
**155**	$C_{38}H_{46}O_{14}$	726.2888	727.2966	R = OAc. R_1 = Tg. R_2 = H. R_3 = $CH(CH_3)_2$. R_4 = OH	OC12C3([H])C(OC(/C(C)=C/C)=O)C(C2)(C)C(C(OC(C)=O)C(OC)=O)C1(C)C45C(C(C6(C)C(O)C5)=CC(OC6C7=COC=C7)=O)(O8)C3OC8(C(C)C)O4
**156**	$C_{40}H_{44}O_{14}$	748.2731	749.2809	R = OAc. R_1 = Bz. R_2 = H. R_3 = $CH(CH_3)_2$. R_4 = OH	OC12C3([H])C(OC(C4=CC=CC=C4)=O)C(C2)(C)C(C(OC(C)=O)C(OC)=O)C1(C)C56C(C(C7(C)C(O)C6)=CC(OC7C8=COC=C8)=O)(O9)C3OC9(C(C)C)O5

**157**	$C_{37}H_{46}O_{11}$	666.3040	667.3118	R = H. R_1 = Tg. R_2 = H. R_3 = $CH_3CHCH_2CH_3$. R_4 = H	OC12C3([H])C(OC(/C(C)=C/C)=O)C(C2)(C)C(C([H])C(OC)=O)C1(C)C45C(C(C6(C)C([H])C5)=CC(OC6C7=COC=C7)=O)(O8)C3OC8(C(C)CC)O4	[1,4,8]
**158**	$C_{39}H_{44}O_{11}$	688.2884	689.2962	R = H. R_1 = Bz. R_2 = H. R_3 = $CH_3CHCH_2CH_3$. R_4 = H	OC12C3([H])C(OC(C4=CC=CC=C4)=O)C(C2)(C)C(C([H])C(OC)=O)C1(C)C56C(C(C7(C)C([H])C6)=CC(OC7C8=COC=C8)=O)(O9)C3OC9(C(C)CC)O5	
**159**	$C_{39}H_{48}O_{13}$	724.3095	725.3173	R = OAc. R_1 = Tg. R_2 = H. R_3 = $CH_3CHCH_2CH_3$. R_4 = H	OC12C3([H])C(OC(/C(C)=C/C)=O)C(C2)(C)C(C(OC(C)=O)C(OC)=O)C1(C)C45C(C(C6(C)C([H])C5)=CC(OC6C7=COC=C7)=O)(O8)C3OC8(C(C)CC)O4	
**160**	$C_{41}H_{46}O_{13}$	746.2938	747.3017	R = OAc. R_1 = Bz. R_2 = H. R_3 = $CH_3CHCH_2CH_3$. R_4 = H	OC12C3([H])C(OC(C4=CC=CC=C4)=O)C(C2)(C)C(C(OC(C)=O)C(OC)=O)C1(C)C56C(C(C7(C)C([H])C6)=CC(OC7C8=COC=C8)=O)(O9)C3OC9(C(C)CC)O5	
**161**	$C_{39}H_{48}O_{13}$	724.3095	725.3173	R = H. R_1 = Tg. R_2 = H. R_3 = $CH_3CHCH_2CH_3$. R_4 = OAc	OC12C3([H])C(OC(/C(C)=C/C)=O)C(C2)(C)C(C([H])C(OC)=O)C1(C)C45C(C(C6(C)C(OC(C)=O)C5)=CC(OC6C7=COC=C7)=O)(O8)C3OC8(C(C)CC)O4	
**162**	$C_{41}H_{46}O_{13}$	746.2938	747.3017	R = H. R_1 = Bz. R_2 = H. R_3 = $CH_3CHCH_2CH_3$. R_4 = OAc	OC12C3([H])C(OC(C4=CC=CC=C4)=O)C(C2)(C)C(C([H])C(OC)=O)C1(C)C56C(C(C	

					7(C)C(OC(C)=O)C6)=CC(OC7C8=COC=C8)=O)(O9)C3OC9(C(C)CC)O5
**163**	$C_{41}H_{50}O_{15}$	782.3150	783.3228	R = OAc. R_1 = Tg. R_2 = H. R_3 = $CH_3CHCH_2CH_3$. R_4 = OAc	OC12C3([H])C(OC(/C(C)=C/C)=O)C(C2)(C)C(C(OC(C)=O)C(OC)=O)C1(C)C45C(C(C6(C)C(OC(C)=O)C5)=CC(OC6C7=COC=C7)=O)(O8)C3OC8(C(C)CC)O4
**164**	$C_{43}H_{48}O_{15}$	804.2993	805.3071	R = OAc. R_1 = Bz. R_2 = H. R_3 = $CH_3CHCH_2CH_3$. R_4 = OAc	OC12C3([H])C(OC(C4=CC=CC=C4)=O)C(C2)(C)C(C(OC(C)=O)C(OC)=O)C1(C)C56C(C(C7(C)C(OC(C)=O)C6)=CC(OC7C8=COC=C8)=O)(O9)C3OC9(C(C)CC)O5
**165**	$C_{37}H_{46}O_{12}$	682.2989	683.3068	R = H. R_1 = Tg. R_2 = H. R_3 = $CH_3CHCH_2CH_3$. R_4 = OH	OC12C3([H])C(OC(/C(C)=C/C)=O)C(C2)(C)C(C([H])C(OC)=O)C1(C)C45C(C(C6(C)C(O)C5)=CC(OC6C7=COC=C7)=O)(O8)C3OC8(C(C)CC)O4
**166**	$C_{39}H_{44}O_{12}$	704.2833	705.2911	R = H. R_1 = Bz. R_2 = H. R_3 = $CH_3CHCH_2CH_3$. R_4 = OH	OC12C3([H])C(OC(C4=CC=CC=C4)=O)C(C2)(C)C(C([H])C(OC)=O)C1(C)C56C(C(C7(C)C(O)C6)=CC(OC7C8=COC=C8)=O)(O9)C3OC9(C(C)CC)O5
**167**	$C_{39}H_{48}O_{14}$	740.3044	741.3122	R = OAc. R_1 = Tg. R_2 = H. R_3 = $CH_3CHCH_2CH_3$. R_4 = OH	OC12C3([H])C(OC(/C(C)=C/C)=O)C(C2)(C)C(C(OC(C)=O)C(OC)=O)C1(C)C45C(C(C6(C)C(O)C5)=CC(OC6C7=COC=C7)=O)(O8)C3OC8(C(C)CC)O4

**168**	$C_{41}H_{46}O_{14}$	762.2888	763.2966	R = OAc. R_1 = Bz. R_2 = H. R_3 = $CH_3CHCH_2CH_3$. R_4 = OH	OC12C3([H])C(OC(C4=CC=CC=C4)=O)C(C2)(C)C(C(OC(C)=O)C(OC)=O)C1(C)C56C(C(C7(C)C(O)C6)=CC(OC7C8=COC=C8)=O)(O9)C3OC9(C(C)CC)O5
**169**	$C_{37}H_{44}O_{11}$	664.2884	665.2962	R = H. R_1 = Tg. R_2 = H. R_3 = (*E*)-CH_3C=$CHCH_3$. R_4 = H	OC12C3([H])C(OC(/C(C)=C/C)=O)C(C2)(C)C(C([H])C(OC)=O)C1(C)C45C(C(C6(C)C([H])C5)=CC(OC6C7=COC=C7)=O)(O8)C3OC8(/C(C)=C/C)O4
**170**	$C_{39}H_{42}O_{11}$	686.2727	687.2805	R = H. R_1 = Bz. R_2 = H. R_3 = (*E*)-CH_3C=$CHCH_3$. R_4 = H	OC12C3([H])C(OC(C4=CC=CC=C4)=O)C(C2)(C)C(C([H])C(OC)=O)C1(C)C56C(C(C7(C)C([H])C6)=CC(OC7C8=COC=C8)=O)(O9)C3OC9(/C(C)=C/C)O5
**171**	$C_{39}H_{46}O_{13}$	722.2938	723.3017	R = OAc. R_1 = Tg. R_2 = H. R_3 = (*E*)-CH_3C=$CHCH_3$. R_4 = H	OC12C3([H])C(OC(/C(C)=C/C)=O)C(C2)(C)C(C(OC(C)=O)C(OC)=O)C1(C)C45C(C(C6(C)C([H])C5)=CC(OC6C7=COC=C7)=O)(O8)C3OC8(/C(C)=C/C)O4
**172**	$C_{41}H_{44}O_{13}$	744.2782	745.2860	R = OAc. R_1 = Bz. R_2 = H. R_3 = (*E*)-CH_3C=$CHCH_3$. R_4 = H	OC12C3([H])C(OC(C4=CC=CC=C4)=O)C(C2)(C)C(C(OC(C)=O)C(OC)=O)C1(C)C56C(C(C7(C)C([H])C6)=CC(OC7C8=COC=C8)=O)(O9)C3OC9(/C(C)=C/C)O5
**173**	$C_{39}H_{46}O_{13}$	722.2938	723.3017	R = H. R_1 = Tg. R_2 = H. R_3 = (*E*)-CH_3C=$CHCH_3$. R_4 = OAc	OC12C3([H])C(OC(/C(C)=C/C)=O)C(C2)(C)C(C([H])C(OC)=O)C1(C)C45C(C(C6(C)

					C(OC(C)=O)C5)=CC(OC6C7=COC=C7)=O)(O8)C3OC8(/C(C)=C/C)O4
**174**	$C_{41}H_{44}O_{13}$	744.2782	745.2860	R = H. R_1 = Bz. R_2 = H. R_3 = (*E*)-CH_3C=$CHCH_3$. R_4 = OAc	OC12C3([H])C(OC(C4=CC=CC=C4)=O)C(C2)(C)C(C([H])C(OC)=O)C1(C)C56C(C(C7(C)C(OC(C)=O)C6)=CC(OC7C8=COC=C8)=O)(O9)C3OC9(/C(C)=C/C)O5
**175**	$C_{41}H_{48}O_{15}$	780.2993	781.3071	R = OAc. R_1 = Tg. R_2 = H. R_3 = (*E*)-CH_3C=$CHCH_3$. R_4 = OAc	OC12C3([H])C(OC(/C(C)=C/C)=O)C(C2)(C)C(C(OC(C)=O)C(OC)=O)C1(C)C45C(C(C6(C)C(OC(C)=O)C5)=CC(OC6C7=COC=C7)=O)(O8)C3OC8(/C(C)=C/C)O4
**176**	$C_{43}H_{46}O_{15}$	802.2837	803.2915	R = OAc. R_1 = Bz. R_2 = H. R_3 = (*E*)-CH_3C=$CHCH_3$. R_4 = OAc	OC12C3([H])C(OC(C4=CC=CC=C4)=O)C(C2)(C)C(C(OC(C)=O)C(OC)=O)C1(C)C56C(C(C7(C)C(OC(C)=O)C6)=CC(OC7C8=COC=C8)=O)(O9)C3OC9(/C(C)=C/C)O5
**177**	$C_{37}H_{44}O_{12}$	680.2833	681.2911	R = H. R_1 = Tg. R_2 = H. R_3 = (*E*)-CH_3C=$CHCH_3$. R_4 = OH	OC12C3([H])C(OC(/C(C)=C/C)=O)C(C2)(C)C(C([H])C(OC)=O)C1(C)C45C(C(C6(C)C(O)C5)=CC(OC6C7=COC=C7)=O)(O8)C3OC8(/C(C)=C/C)O4
**178**	$C_{39}H_{42}O_{12}$	702.2676	703.2755	R = H. R_1 = Bz. R_2 = H. R_3 = (*E*)-CH_3C=$CHCH_3$. R_4 = OH	OC12C3([H])C(OC(C4=CC=CC=C4)=O)C(C2)(C)C(C([H])C(OC)=O)C1(C)C56C(C(C7(C)C(O)C6)=CC(OC7C8=COC=C8)=O)(O9)C3OC9(/C(C)=C/C)O5

**179**	$C_{39}H_{46}O_{14}$	738.2888	739.2966	R = OAc. R_1 = Tg. R_2 = H. R_3 = (*E*)-CH_3C=$CHCH_3$. R_4 = OH	OC12C3([H])C(OC(/C(C)=C/C)=O)C(C2)(C)C(C(OC(C)=O)C(OC)=O)C1(C)C45C(C(C6(C)C(O)C5)=CC(OC6C7=COC=C7)=O)(O8)C3OC8(/C(C)=C/C)O4
**180**	$C_{41}H_{44}O_{14}$	760.2731	761.2809	R = OAc. R_1 = Bz. R_2 = H. R_3 = (*E*)-CH_3C=$CHCH_3$. R_4 = OH	OC12C3([H])C(OC(C4=CC=CC=C4)=O)C(C2)(C)C(C(OC(C)=O)C(OC)=O)C1(C)C56C(C(C7(C)C(O)C6)=CC(OC7C8=COC=C8)=O)(O9)C3OC9(/C(C)=C/C)O5
**181**	$C_{34}H_{40}O_{13}$	656.2469	657.2547	R = OH. R_1 = Tg. R_2 = OH. R_3 = CH_3. R_4 = H	OC12C3(O)C(OC(/C(C)=C/C)=O)C(C2)(C)C(C(O)C(OC)=O)C1(C)C45C(C(C6(C)C([H])C5)=CC(OC6C7=COC=C7)=O)(O8)C3OC8(C)O4
**182**	$C_{36}H_{38}O_{13}$	678.2312	679.2391	R = OH. R_1 = Bz. R_2 = OH. R_3 = CH_3. R_4 = H	OC12C3(O)C(OC(C4=CC=CC=C4)=O)C(C2)(C)C(C(O)C(OC)=O)C1(C)C56C(C(C7(C)C([H])C6)=CC(OC7C8=COC=C8)=O)(O9)C3OC9(C)O5
**183**	$C_{36}H_{42}O_{14}$	698.2575	699.2653	R = OH. R_1 = Tg. R_2 = OAc. R_3 = CH_3. R_4 = H	OC12C3(OC(C)=O)C(OC(/C(C)=C/C)=O)C(C2)(C)C(C(O)C(OC)=O)C1(C)C45C(C(C6(C)C([H])C5)=CC(OC6C7=COC=C7)=O)(O8)C3OC8(C)O4
**184**	$C_{38}H_{40}O_{14}$	720.2418	721.2496	R = OH. R_1 = Bz. R_2 = OAc. R_3 = CH_3. R_4 = H	OC12C3(OC(C)=O)C(OC(C4=CC=CC=C4)=O)C(C2)(C)C(C(O)C(OC)=O)C1(C)C56C(

					C(C7(C)C([H])C6)=CC(OC7C8=COC=C8)=O)(O9)C3OC9(C)O5
**185**	$C_{36}H_{42}O_{15}$	714.2524	715.2602	R = OH. R_1 = Tg. R_2 = OH. R_3 = CH_3. R_4 = OAc	OC12C3(O)C(OC(/C(C)=C/C)=O)C(C2)(C)C(C(O)C(OC)=O)C1(C)C45C(C(C6(C)C(OC(C)=O)C5)=CC(OC6C7=COC=C7)=O)(O8)C3OC8(C)O4
**186**	$C_{38}H_{40}O_{15}$	736.2367	737.2445	R = OH. R_1 = Bz. R_2 = OH. R_3 = CH_3. R_4 = OAc	OC12C3(O)C(OC(C4=CC=CC=C4)=O)C(C2)(C)C(C(O)C(OC)=O)C1(C)C56C(C(C7(C)C(OC(C)=O)C6)=CC(OC7C8=COC=C8)=O)(O9)C3OC9(C)O5
**187**	$C_{38}H_{44}O_{16}$	756.2629	757.2708	R = OH. R_1 = Tg. R_2 = OAc. R_3 = CH_3. R_4 = OAc	OC12C3(OC(C)=O)C(OC(/C(C)=C/C)=O)C(C2)(C)C(C(O)C(OC)=O)C1(C)C45C(C(C6(C)C(OC(C)=O)C5)=CC(OC6C7=COC=C7)=O)(O8)C3OC8(C)O4
**188**	$C_{40}H_{42}O_{16}$	778.2473	779.2551	R = OH. R_1 = Bz. R_2 = OAc. R_3 = CH_3. R_4 = OAc	OC12C3(OC(C)=O)C(OC(C4=CC=CC=C4)=O)C(C2)(C)C(C(O)C(OC)=O)C1(C)C56C(C(C7(C)C(OC(C)=O)C6)=CC(OC7C8=COC=C8)=O)(O9)C3OC9(C)O5
**189**	$C_{35}H_{42}O_{13}$	670.2625	671.2704	R = OH. R_1 = Tg. R_2 = OH. R_3 = CH_2CH_3. R_4 = H	OC12C3(O)C(OC(/C(C)=C/C)=O)C(C2)(C)C(C(O)C(OC)=O)C1(C)C45C(C(C6(C)C([H])C5)=CC(OC6C7=COC=C7)=O)(O8)C3OC8(CC)O4

**190**	$C_{37}H_{40}O_{13}$	692.2469	693.2547	R = OH. R_1 = Bz. R_2 = OH. R_3 = CH_2CH_3. R_4 = H	OC12C3(O)C(OC(C4=CC=CC=C4)=O)C(C2)(C)C(C(O)C(OC)=O)C1(C)C56C(C(C7(C)C([H])C6)=CC(OC7C8=COC=C8)=O)(O9)C3OC9(CC)O5
**191**	$C_{37}H_{44}O_{14}$	712.2731	713.2809	R = OH. R_1 = Tg. R_2 = OAc. R_3 = CH_2CH_3. R_4 = H	OC12C3(OC(C)=O)C(OC(/C(C)=C/C)=O)C(C2)(C)C(C(O)C(OC)=O)C1(C)C45C(C(C6(C)C([H])C5)=CC(OC6C7=COC=C7)=O)(O8)C3OC8(CC)O4
**192**	$C_{39}H_{42}O_{14}$	734.2575	735.2653	R = OH. R_1 = Bz. R_2 = OAc. R_3 = CH_2CH_3. R_4 = H	OC12C3(OC(C)=O)C(OC(C4=CC=CC=C4)=O)C(C2)(C)C(C(O)C(OC)=O)C1(C)C56C(C(C7(C)C([H])C6)=CC(OC7C8=COC=C8)=O)(O9)C3OC9(CC)O5
**193**	$C_{37}H_{44}O_{15}$	728.2680	729.2758	R = OH. R_1 = Tg. R_2 = OH. R_3 = CH_2CH_3. R_4 = OAc	OC12C3(O)C(OC(/C(C)=C/C)=O)C(C2)(C)C(C(O)C(OC)=O)C1(C)C45C(C(C6(C)C(OC(C)=O)C5)=CC(OC6C7=COC=C7)=O)(O8)C3OC8(CC)O4
**194**	$C_{39}H_{42}O_{15}$	750.2524	751.2602	R = OH. R_1 = Bz. R_2 = OH. R_3 = CH_2CH_3. R_4 = OAc	OC12C3(O)C(OC(C4=CC=CC=C4)=O)C(C2)(C)C(C(O)C(OC)=O)C1(C)C56C(C(C7(C)C(OC(C)=O)C6)=CC(OC7C8=COC=C8)=O)(O9)C3OC9(CC)O5
**195**	$C_{39}H_{46}O_{16}$	770.2786	771.2864	R = OH. R_1 = Tg. R_2 = OAc. R_3 = CH_2CH_3. R_4 = OAc	OC12C3(OC(C)=O)C(OC(/C(C)=C/C)=O)C(C2)(C)C(C(O)C(OC)=O)C1(C)C45C(C(

					C6(C)C(OC(C)=O)C5)=CC(OC6C7=COC=C7)=O)(O8)C3OC8(CC)O4
**196**	$C_{41}H_{44}O_{16}$	792.2629	793.2708	R = OH. R_1 = Bz. R_2 = OAc. R_3 = CH_2CH_3. R_4 = OAc	OC12C3(OC(C)=O)C(OC(C4=CC=CC=C4)=O)C(C2)(C)C(C(O)C(OC)=O)C1(C)C56C(C(C7(C)C(OC(C)=O)C6)=CC(OC7C8=COC=C8)=O)(O9)C3OC9(CC)O5
**197**	$C_{36}H_{44}O_{13}$	684.2782	685.2860	R = OH. R_1 = Tg. R_2 = OH. R_3 = $CH(CH_3)_2$. R_4 = H	OC12C3(O)C(OC(/C(C)=C/C)=O)C(C2)(C)C(C(O)C(OC)=O)C1(C)C45C(C(C6(C)C([H])C5)=CC(OC6C7=COC=C7)=O)(O8)C3OC8(C(C)C)O4
**198**	$C_{38}H_{42}O_{13}$	706.2625	707.2704	R = OH. R_1 = Bz. R_2 = OH. R_3 = $CH(CH_3)_2$. R_4 = H	OC12C3(O)C(OC(C4=CC=CC=C4)=O)C(C2)(C)C(C(O)C(OC)=O)C1(C)C56C(C(C7(C)C([H])C6)=CC(OC7C8=COC=C8)=O)(O9)C3OC9(C(C)C)O5
**199**	$C_{38}H_{46}O_{14}$	726.2888	727.2966	R = OH. R_1 = Tg. R_2 = OAc. R_3 = $CH(CH_3)_2$. R_4 = H	OC12C3(OC(C)=O)C(OC(/C(C)=C/C)=O)C(C2)(C)C(C(O)C(OC)=O)C1(C)C45C(C(C6(C)C([H])C5)=CC(OC6C7=COC=C7)=O)(O8)C3OC8(C(C)C)O4
**200**	$C_{40}H_{44}O_{14}$	748.2731	749.2809	R = OH. R_1 = Bz. R_2 = OAc. R_3 = $CH(CH_3)_2$. R_4 = H	OC12C3(OC(C)=O)C(OC(C4=CC=CC=C4)=O)C(C2)(C)C(C(O)C(OC)=O)C1(C)C56C(C(C7(C)C([H])C6)=CC(OC7C8=COC=C8)=O)(O9)C3OC9(C(C)C)O5

**201**	$C_{38}H_{46}O_{15}$	742.2837	743.2915	R = OH. R_1 = Tg. R_2 = OH. R_3 = $CH(CH_3)_2$. R_4 = OAc	OC12C3(O)C(OC(/C(C)=C/C)=O)C(C2)(C)C(C(O)C(OC)=O)C1(C)C45C(C(C6(C)C(OC(C)=O)C5)=CC(OC6C7=COC=C7)=O)(O8)C3OC8(C(C)C)O4	
**202**	$C_{40}H_{44}O_{15}$	764.2680	765.2758	R = OH. R_1 = Bz. R_2 = OH. R_3 = $CH(CH_3)_2$. R_4 = OAc	OC12C3(O)C(OC(C4=CC=CC=C4)=O)C(C2)(C)C(C(O)C(OC)=O)C1(C)C56C(C(C7(C)C(OC(C)=O)C6)=CC(OC7C8=COC=C8)=O)(O9)C3OC9(C(C)C)O5	
**203**	$C_{40}H_{48}O_{16}$	784.2942	785.3021	R = OH. R_1 = Tg. R_2 = OAc. R_3 = $CH(CH_3)_2$. R_4 = OAc	OC12C3(OC(C)=O)C(OC(/C(C)=C/C)=O)C(C2)(C)C(C(O)C(OC)=O)C1(C)C45C(C(C6(C)C(OC(C)=O)C5)=CC(OC6C7=COC=C7)=O)(O8)C3OC8(C(C)C)O4	
**204**	$C_{42}H_{46}O_{16}$	806.2786	807.2864	R = OH. R_1 = Bz. R_2 = OAc. R_3 = $CH(CH_3)_2$. R_4 = OAc	OC12C3(OC(C)=O)C(OC(C4=CC=CC=C4)=O)C(C2)(C)C(C(O)C(OC)=O)C1(C)C56C(C(C7(C)C(OC(C)=O)C6)=CC(OC7C8=COC=C8)=O)(O9)C3OC9(C(C)C)O5	
**205**	$C_{37}H_{46}O_{13}$	698.2938	699.3017	R = OH. R_1 = Tg. R_2 = OH. R_3 = $CH_3CHCH_2CH_3$. R_4 = H	OC12C3(O)C(OC(/C(C)=C/C)=O)C(C2)(C)C(C(O)C(OC)=O)C1(C)C45C(C(C6(C)C([H])C5)=CC(OC6C7=COC=C7)=O)(O8)C3OC8(C(C)CC)O4	[1]
**206**	$C_{39}H_{44}O_{13}$	720.2782	721.2860	R = OH. R_1 = Bz. R_2 = OH. R_3 = $CH_3CHCH_2CH_3$. R_4 = H	OC12C3(O)C(OC(C4=CC=CC=C4)=O)C(C2)(C)C(C(O)C(OC)=O)C1(C)C56C(C(C7(C	

					)C([H])C6)=CC(OC7C8=COC=C8)=O)(O9)C3OC9(C(C)CC)O5
**207**	$C_{39}H_{48}O_{14}$	740.3044	741.3122	R = OH. R_1 = Tg. R_2 = OAc. R_3 = $CH_3CHCH_2CH_3$. R_4 = H	OC12C3(OC(C)=O)C(OC(/C(C)=C/C)=O)C(C2)(C)C(C(O)C(OC)=O)C1(C)C45C(C(C6(C)C([H])C5)=CC(OC6C7=COC=C7)=O)(O8)C3OC8(C(C)CC)O4
**208**	$C_{41}H_{46}O_{14}$	762.2888	763.2966	R = OH. R_1 = Bz. R_2 = OAc. R_3 = $CH_3CHCH_2CH_3$. R_4 = H	OC12C3(OC(C)=O)C(OC(C4=CC=CC=C4)=O)C(C2)(C)C(C(O)C(OC)=O)C1(C)C56C(C(C7(C)C([H])C6)=CC(OC7C8=COC=C8)=O)(O9)C3OC9(C(C)CC)O5
**209**	$C_{39}H_{48}O_{15}$	756.2993	757.3071	R = OH. R_1 = Tg. R_2 = OH. R_3 = $CH_3CHCH_2CH_3$. R_4 = OAc	OC12C3(O)C(OC(/C(C)=C/C)=O)C(C2)(C)C(C(O)C(OC)=O)C1(C)C45C(C(C6(C)C(OC(C)=O)C5)=CC(OC6C7=COC=C7)=O)(O8)C3OC8(C(C)CC)O4
**210**	$C_{41}H_{46}O_{15}$	778.2837	779.2915	R = OH. R_1 = Bz. R_2 = OH. R_3 = $CH_3CHCH_2CH_3$. R_4 = OAc	OC12C3(O)C(OC(C4=CC=CC=C4)=O)C(C2)(C)C(C(O)C(OC)=O)C1(C)C56C(C(C7(C)C(OC(C)=O)C6)=CC(OC7C8=COC=C8)=O)(O9)C3OC9(C(C)CC)O5
**211**	$C_{41}H_{50}O_{16}$	798.3099	799.3177	R = OH. R_1 = Tg. R_2 = OAc. R_3 = $CH_3CHCH_2CH_3$. R_4 = OAc	OC12C3(OC(C)=O)C(OC(/C(C)=C/C)=O)C(C2)(C)C(C(O)C(OC)=O)C1(C)C45C(C(C6(C)C(OC(C)=O)C5)=CC(OC6C7=COC=C7)=O)(O8)C3OC8(C(C)CC)O4

**212**	$C_{43}H_{48}O_{16}$	820.2942	821.3021	R = OH. R_1 = Bz. R_2 = OAc. R_3 = $CH_3CHCH_2CH_3$. R_4 = OAc	OC12C3(OC(C)=O)C(OC(C4=CC=CC=C4)=O)C(C2)(C)C(C(O)C(OC)=O)C1(C)C56C(C(C7(C)C(OC(C)=O)C6)=CC(OC7C8=COC=C8)=O)(O9)C3OC9(C(C)CC)O5
**213**	$C_{37}H_{44}O_{13}$	696.2782	697.2860	R = OH. R_1 = Tg. R_2 = OH. R_3 = (*E*)-CH_3C=$CHCH_3$. R_4 = H	OC12C3(O)C(OC(/C(C)=C/C)=O)C(C2)(C)C(C(O)C(OC)=O)C1(C)C45C(C(C6(C)C([H])C5)=CC(OC6C7=COC=C7)=O)(O8)C3OC8(/C(C)=C/C)O4
**214**	$C_{39}H_{42}O_{13}$	718.2625	719.2704	R = OH. R_1 = Bz. R_2 = OH. R_3 = (*E*)-CH_3C=$CHCH_3$. R_4 = H	OC12C3(O)C(OC(C4=CC=CC=C4)=O)C(C2)(C)C(C(O)C(OC)=O)C1(C)C56C(C(C7(C)C([H])C6)=CC(OC7C8=COC=C8)=O)(O9)C3OC9(/C(C)=C/C)O5
**215**	$C_{39}H_{46}O_{14}$	738.2888	739.2966	R = OH. R_1 = Tg. R_2 = OAc. R_3 = (*E*)-CH_3C=$CHCH_3$. R_4 = H	OC12C3(OC(C)=O)C(OC(/C(C)=C/C)=O)C(C2)(C)C(C(O)C(OC)=O)C1(C)C45C(C(C6(C)C([H])C5)=CC(OC6C7=COC=C7)=O)(O8)C3OC8(/C(C)=C/C)O4
**216**	$C_{41}H_{44}O_{14}$	760.2731	761.2809	R = OH. R_1 = Bz. R_2 = OAc. R_3 = (*E*)-CH_3C=$CHCH_3$. R_4 = H	OC12C3(OC(C)=O)C(OC(C4=CC=CC=C4)=O)C(C2)(C)C(C(O)C(OC)=O)C1(C)C56C(C(C7(C)C([H])C6)=CC(OC7C8=COC=C8)=O)(O9)C3OC9(/C(C)=C/C)O5
**217**	$C_{39}H_{46}O_{15}$	754.2837	755.2915	R = OH. R_1 = Tg. R_2 = OH. R_3 = (*E*)-CH_3C=$CHCH_3$. R_4 = OAc	OC12C3(O)C(OC(/C(C)=C/C)=O)C(C2)(C)C(C(O)C(OC)=O)C1(C)C45C(C(C6(C)C(O

					C(C)=O)C5)=CC(OC6C7=COC=C7)=O)(O8)C3OC8(/C(C)=C/C)O4
**218**	$C_{41}H_{44}O_{15}$	776.2680	777.2758	R = OH. R_1 = Bz. R_2 = OH. R_3 = (*E*)-CH_3C=$CHCH_3$. R_4 = OAc	OC12C3(O)C(OC(C4=CC=CC=C4)=O)C(C2)(C)C(C(O)C(OC)=O)C1(C)C56C(C(C7(C)C(OC(C)=O)C6)=CC(OC7C8=COC=C8)=O)(O9)C3OC9(/C(C)=C/C)O5
**219**	$C_{41}H_{48}O_{16}$	796.2942	797.3021	R = OH. R_1 = Tg. R_2 = OAc. R_3 = (*E*)-CH_3C=$CHCH_3$. R_4 = OAc	OC12C3(OC(C)=O)C(OC(/C(C)=C/C)=O)C(C2)(C)C(C(O)C(OC)=O)C1(C)C45C(C(C6(C)C(OC(C)=O)C5)=CC(OC6C7=COC=C7)=O)(O8)C3OC8(/C(C)=C/C)O4
**220**	$C_{43}H_{46}O_{16}$	818.2786	819.2864	R = OH. R_1 = Bz. R_2 = OAc. R_3 = (*E*)-CH_3C=$CHCH_3$. R_4 = OAc	OC12C3(OC(C)=O)C(OC(C4=CC=CC=C4)=O)C(C2)(C)C(C(O)C(OC)=O)C1(C)C56C(C(C7(C)C(OC(C)=O)C6)=CC(OC7C8=COC=C8)=O)(O9)C3OC9(/C(C)=C/C)O5
**221**	$C_{34}H_{40}O_{14}$	672.2418	673.2496	R = OH. R_1 = Tg. R_2 = OH. R_3 = CH_3. R_4 = OH	OC12C3(O)C(OC(/C(C)=C/C)=O)C(C2)(C)C(C(O)C(OC)=O)C1(C)C45C(C(C6(C)C(O)C5)=CC(OC6C7=COC=C7)=O)(O8)C3OC8(C)O4
**222**	$C_{36}H_{38}O_{14}$	694.2262	695.2340	R = OH. R_1 = Bz. R_2 = OH. R_3 = CH_3. R_4 = OH	OC12C3(O)C(OC(C4=CC=CC=C4)=O)C(C2)(C)C(C(O)C(OC)=O)C1(C)C56C(C(C7(C)C(O)C6)=CC(OC7C8=COC=C8)=O)(O9)C3OC9(C)O5

**223**	$C_{36}H_{42}O_{15}$	714.2524	715.2602	R = OH. R_1 = Tg. R_2 = OAc. R_3 = CH_3. R_4 = OH	OC12C3(OC(C)=O)C(OC(/C(C)=C/C)=O)C(C2)(C)C(C(O)C(OC)=O)C1(C)C45C(C(C6(C)C(O)C5)=CC(OC6C7=COC=C7)=O)(O8)C3OC8(C)O4
**224**	$C_{38}H_{40}O_{15}$	736.2367	737.2445	R = OH. R_1 = Bz. R_2 = OAc. R_3 = CH_3. R_4 = OH	OC12C3(OC(C)=O)C(OC(C4=CC=CC=C4)=O)C(C2)(C)C(C(O)C(OC)=O)C1(C)C56C(C(C7(C)C(O)C6)=CC(OC7C8=COC=C8)=O)(O9)C3OC9(C)O5
**225**	$C_{35}H_{42}O_{14}$	686.2575	687.2653	R = OH. R_1 = Tg. R_2 = OH. R_3 = CH_2CH_3. R_4 = OH	OC12C3(O)C(OC(/C(C)=C/C)=O)C(C2)(C)C(C(O)C(OC)=O)C1(C)C45C(C(C6(C)C(O)C5)=CC(OC6C7=COC=C7)=O)(O8)C3OC8(CC)O4
**226**	$C_{37}H_{40}O_{14}$	708.2418	709.2496	R = OH. R_1 = Bz. R_2 = OH. R_3 = CH_2CH_3. R_4 = OH	OC12C3(O)C(OC(C4=CC=CC=C4)=O)C(C2)(C)C(C(O)C(OC)=O)C1(C)C56C(C(C7(C)C(O)C6)=CC(OC7C8=COC=C8)=O)(O9)C3OC9(CC)O5
**227**	$C_{37}H_{44}O_{15}$	728.2680	729.2758	R = OH. R_1 = Tg. R_2 = OAc. R_3 = CH_2CH_3. R_4 = OH	OC12C3(OC(C)=O)C(OC(/C(C)=C/C)=O)C(C2)(C)C(C(O)C(OC)=O)C1(C)C45C(C(C6(C)C(O)C5)=CC(OC6C7=COC=C7)=O)(O8)C3OC8(CC)O4
**228**	$C_{39}H_{42}O_{15}$	750.2524	751.2602	R = OH. R_1 = Bz. R_2 = OAc. R_3 = CH_2CH_3. R_4 = OH	OC12C3(OC(C)=O)C(OC(C4=CC=CC=C4)=O)C(C2)(C)C(C(O)C(OC)=O)C1(C)C56C(

					C(C7(C)C(O)C6)=CC(OC7C8=COC=C8)=O)(O9)C3OC9(CC)O5
**229**	$C_{36}H_{44}O_{14}$	700.2731	701.2809	R = OH. R_1 = Tg. R_2 = OH. R_3 = $CH(CH_3)_2$. R_4 = OH	OC12C3(O)C(OC(/C(C)=C/C)=O)C(C2)(C)C(C(O)C(OC)=O)C1(C)C45C(C(C6(C)C(O)C5)=CC(OC6C7=COC=C7)=O)(O8)C3OC8(C(C)C)O4
**230**	$C_{38}H_{42}O_{14}$	722.2575	723.2653	R = OH. R_1 = Bz. R_2 = OH. R_3 = $CH(CH_3)_2$. R_4 = OH	OC12C3(O)C(OC(C4=CC=CC=C4)=O)C(C2)(C)C(C(O)C(OC)=O)C1(C)C56C(C(C7(C)C(O)C6)=CC(OC7C8=COC=C8)=O)(O9)C3OC9(C(C)C)O5
**231**	$C_{38}H_{46}O_{15}$	742.2837	743.2915	R = OH. R_1 = Tg. R_2 = OAc. R_3 = $CH(CH_3)_2$. R_4 = OH	OC12C3(OC(C)=O)C(OC(/C(C)=C/C)=O)C(C2)(C)C(C(O)C(OC)=O)C1(C)C45C(C(C6(C)C(O)C5)=CC(OC6C7=COC=C7)=O)(O8)C3OC8(C(C)C)O4
**232**	$C_{40}H_{44}O_{15}$	764.2680	765.2758	R = OH. R_1 = Bz. R_2 = OAc. R_3 = $CH(CH_3)_2$. R_4 = OH	OC12C3(OC(C)=O)C(OC(C4=CC=CC=C4)=O)C(C2)(C)C(C(O)C(OC)=O)C1(C)C56C(C(C7(C)C(O)C6)=CC(OC7C8=COC=C8)=O)(O9)C3OC9(C(C)C)O5
**233**	$C_{37}H_{46}O_{14}$	714.2888	715.2966	R = OH. R_1 = Tg. R_2 = OH. R_3 = $CH_3CHCH_2CH_3$. R_4 = OH	OC12C3(O)C(OC(/C(C)=C/C)=O)C(C2)(C)C(C(O)C(OC)=O)C1(C)C45C(C(C6(C)C(O)C5)=CC(OC6C7=COC=C7)=O)(O8)C3OC8(C(C)CC)O4

**234**	$C_{39}H_{44}O_{14}$	736.2731	737.2809	R = OH. R_1 = Bz. R_2 = OH. R_3 = $CH_3CHCH_2CH_3$. R_4 = OH	OC12C3(O)C(OC(C4=CC=CC=C4)=O)C(C2)(C)C(C(O)C(OC)=O)C1(C)C56C(C(C7(C)C(O)C6)=CC(OC7C8=COC=C8)=O)(O9)C3OC9(C(C)CC)O5
**235**	$C_{39}H_{48}O_{15}$	756.2993	757.3071	R = OH. R_1 = Tg. R_2 = OAc. R_3 = $CH_3CHCH_2CH_3$. R_4 = OH	OC12C3(OC(C)=O)C(OC(/C(C)=C/C)=O)C(C2)(C)C(C(O)C(OC)=O)C1(C)C45C(C(C6(C)C(O)C5)=CC(OC6C7=COC=C7)=O)(O8)C3OC8(C(C)CC)O4
**236**	$C_{41}H_{46}O_{15}$	778.2837	779.2915	R = OH. R_1 = Bz. R_2 = OAc. R_3 = $CH_3CHCH_2CH_3$. R_4 = OH	OC12C3(OC(C)=O)C(OC(C4=CC=CC=C4)=O)C(C2)(C)C(C(O)C(OC)=O)C1(C)C56C(C(C7(C)C(O)C6)=CC(OC7C8=COC=C8)=O)(O9)C3OC9(C(C)CC)O5
**237**	$C_{37}H_{44}O_{14}$	712.2731	713.2809	R = OH. R_1 = Tg. R_2 = OH. R_3 = (*E*)-$CH_3C{=}CHCH_3$. R_4 = OH	OC12C3(O)C(OC(/C(C)=C/C)=O)C(C2)(C)C(C(O)C(OC)=O)C1(C)C45C(C(C6(C)C(O)C5)=CC(OC6C7=COC=C7)=O)(O8)C3OC8(/C(C)=C/C)O4
**238**	$C_{39}H_{42}O_{14}$	734.2575	735.2653	R = OH. R_1 = Bz. R_2 = OH. R_3 = (*E*)-$CH_3C{=}CHCH_3$. R_4 = OH	OC12C3(O)C(OC(C4=CC=CC=C4)=O)C(C2)(C)C(C(O)C(OC)=O)C1(C)C56C(C(C7(C)C(O)C6)=CC(OC7C8=COC=C8)=O)(O9)C3OC9(/C(C)=C/C)O5
**239**	$C_{39}H_{46}O_{15}$	754.2837	755.2915	R = OH. R_1 = Tg. R_2 = OAc. R_3 = (*E*)-$CH_3C{=}CHCH_3$. R_4 = OH	OC12C3(OC(C)=O)C(OC(/C(C)=C/C)=O)C(C2)(C)C(C(O)C(OC)=O)C1(C)C45C(C(

					C6(C)C(O)C5)=CC(OC6C7=COC=C7)=O)(O8)C3OC8(/C(C)=C/C)O4
**240**	$C_{41}H_{44}O_{15}$	776.2680	777.2758	R = OH. R_1 = Bz. R_2 = OAc. R_3 = (*E*)-CH_3C=$CHCH_3$. R_4 = OH	OC12C3(OC(C)=O)C(OC(C4=CC=CC=C4)=O)C(C2)(C)C(C(O)C(OC)=O)C1(C)C56C(C(C7(C)C(O)C6)=CC(OC7C8=COC=C8)=O)(O9)C3OC9(/C(C)=C/C)O5
**241**	$C_{34}H_{40}O_{12}$	640.2520	641.2598	R = OH. R_1 = Tg. R_2 = H. R_3 = CH_3. R_4 = H	OC12C3([H])C(OC(/C(C)=C/C)=O)C(C2)(C)C(C(O)C(OC)=O)C1(C)C45C(C(C6(C)C([H])C5)=CC(OC6C7=COC=C7)=O)(O8)C3OC8(C)O4
**242**	$C_{36}H_{38}O_{12}$	662.2363	663.2442	R = OH. R_1 = Bz. R_2 = H. R_3 = CH_3. R_4 = H	OC12C3([H])C(OC(C4=CC=CC=C4)=O)C(C2)(C)C(C(O)C(OC)=O)C1(C)C56C(C(C7(C)C([H])C6)=CC(OC7C8=COC=C8)=O)(O9)C3OC9(C)O5
**243**	$C_{36}H_{42}O_{14}$	698.2575	699.2653	R = OH. R_1 = Tg. R_2 = H. R_3 = CH_3. R_4 = OAc	OC12C3([H])C(OC(/C(C)=C/C)=O)C(C2)(C)C(C(O)C(OC)=O)C1(C)C45C(C(C6(C)C(OC(C)=O)C5)=CC(OC6C7=COC=C7)=O)(O8)C3OC8(C)O4
**244**	$C_{38}H_{40}O_{14}$	720.2418	721.2496	R = OH. R_1 = Bz. R_2 = H. R_3 = CH_3. R_4 = OAc	OC12C3([H])C(OC(C4=CC=CC=C4)=O)C(C2)(C)C(C(O)C(OC)=O)C1(C)C56C(C(C7(C)C(OC(C)=O)C6)=CC(OC7C8=COC=C8)=O)(O9)C3OC9(C)O5

**245**	$C_{34}H_{40}O_{13}$	656.2469	657.2547	R = OH. R_1 = Tg. R_2 = H. R_3 = CH_3. R_4 = OH	OC12C3([H])C(OC(/C(C)=C/C)=O)C(C2)(C)C(C(O)C(OC)=O)C1(C)C45C(C(C6(C)C(O)C5)=CC(OC6C7=COC=C7)=O)(O8)C3OC8(C)O4
**246**	$C_{36}H_{38}O_{13}$	678.2312	679.2391	R = OH. R_1 = Bz. R_2 = H. R_3 = CH_3. R_4 = OH	OC12C3([H])C(OC(C4=CC=CC=C4)=O)C(C2)(C)C(C(O)C(OC)=O)C1(C)C56C(C(C7(C)C(O)C6)=CC(OC7C8=COC=C8)=O)(O9)C3OC9(C)O5
**247**	$C_{35}H_{42}O_{12}$	654.2676	655.2755	R = OH. R_1 = Tg. R_2 = H. R_3 = CH_2CH_3. R_4 = H	OC12C3([H])C(OC(/C(C)=C/C)=O)C(C2)(C)C(C(O)C(OC)=O)C1(C)C45C(C(C6(C)C([H])C5)=CC(OC6C7=COC=C7)=O)(O8)C3OC8(CC)O4
**248**	$C_{37}H_{40}O_{12}$	676.2520	677.2598	R = OH. R_1 = Bz. R_2 = H. R_3 = CH_2CH_3. R_4 = H	OC12C3([H])C(OC(C4=CC=CC=C4)=O)C(C2)(C)C(C(O)C(OC)=O)C1(C)C56C(C(C7(C)C([H])C6)=CC(OC7C8=COC=C8)=O)(O9)C3OC9(CC)O5
**249**	$C_{37}H_{44}O_{14}$	712.2731	713.2809	R = OH. R_1 = Tg. R_2 = H. R_3 = CH_2CH_3. R_4 = OAc	OC12C3([H])C(OC(/C(C)=C/C)=O)C(C2)(C)C(C(O)C(OC)=O)C1(C)C45C(C(C6(C)C(OC(C)=O)C5)=CC(OC6C7=COC=C7)=O)(O8)C3OC8(CC)O4
**250**	$C_{39}H_{42}O_{14}$	734.2575	735.2653	R = OH. R_1 = Bz. R_2 = H. R_3 = CH_2CH_3. R_4 = OAc	OC12C3([H])C(OC(C4=CC=CC=C4)=O)C(C2)(C)C(C(O)C(OC)=O)C1(C)C56C(C(C7(

					C)C(OC(C)=O)C6)=CC(OC7C8=COC=C8)=O)(O9)C3OC9(CC)O5
**251**	$C_{35}H_{42}O_{13}$	670.2625	671.2704	R = OH. R_1 = Tg. R_2 = H. R_3 = CH_2CH_3. R_4 = OH	OC12C3([H])C(OC(/C(C)=C/C)=O)C(C2)(C)C(C(O)C(OC)=O)C1(C)C45C(C(C6(C)C(O)C5)=CC(OC6C7=COC=C7)=O)(O8)C3OC8(CC)O4
**252**	$C_{37}H_{40}O_{13}$	692.2469	693.2547	R = OH. R_1 = Bz. R_2 = H. R_3 = CH_2CH_3. R_4 = OH	OC12C3([H])C(OC(C4=CC=CC=C4)=O)C(C2)(C)C(C(O)C(OC)=O)C1(C)C56C(C(C7(C)C(O)C6)=CC(OC7C8=COC=C8)=O)(O9)C3OC9(CC)O5
**253**	$C_{36}H_{44}O_{12}$	668.2833	669.2911	R = OH. R_1 = Tg. R_2 = H. R_3 = $CH(CH_3)_2$. R_4 = H	OC12C3([H])C(OC(/C(C)=C/C)=O)C(C2)(C)C(C(O)C(OC)=O)C1(C)C45C(C(C6(C)C([H])C5)=CC(OC6C7=COC=C7)=O)(O8)C3OC8(C(C)C)O4
**254**	$C_{38}H_{42}O_{12}$	690.2676	691.2755	R = OH. R_1 = Bz. R_2 = H. R_3 = $CH(CH_3)_2$. R_4 = H	OC12C3([H])C(OC(C4=CC=CC=C4)=O)C(C2)(C)C(C(O)C(OC)=O)C1(C)C56C(C(C7(C)C([H])C6)=CC(OC7C8=COC=C8)=O)(O9)C3OC9(C(C)C)O5
**255**	$C_{38}H_{46}O_{14}$	726.2888	727.2966	R = OH. R_1 = Tg. R_2 = H. R_3 = $CH(CH_3)_2$. R_4 = OAc	OC12C3([H])C(OC(/C(C)=C/C)=O)C(C2)(C)C(C(O)C(OC)=O)C1(C)C45C(C(C6(C)C(OC(C)=O)C5)=CC(OC6C7=COC=C7)=O)(O8)C3OC8(C(C)C)O4

**256**	$C_{40}H_{44}O_{14}$	748.2731	749.2809	R = OH. R_1 = Bz. R_2 = H. R_3 = $CH(CH_3)_2$. R_4 = OAc	OC12C3([H])C(OC(C4=CC=CC=C4)=O)C(C2)(C)C(C(O)C(OC)=O)C1(C)C56C(C(C7(C)C(OC(C)=O)C6)=CC(OC7C8=COC=C8)=O)(O9)C3OC9(C(C)C)O5
**257**	$C_{36}H_{44}O_{13}$	684.2782	685.2860	R = OH. R_1 = Tg. R_2 = H. R_3 = $CH(CH_3)_2$. R_4 = OH	OC12C3([H])C(OC(/C(C)=C/C)=O)C(C2)(C)C(C(O)C(OC)=O)C1(C)C45C(C(C6(C)C(O)C5)=CC(OC6C7=COC=C7)=O)(O8)C3OC8(C(C)C)O4
**258**	$C_{38}H_{42}O_{13}$	706.2625	707.2704	R = OH. R_1 = Bz. R_2 = H. R_3 = $CH(CH_3)_2$. R_4 = OH	OC12C3([H])C(OC(C4=CC=CC=C4)=O)C(C2)(C)C(C(O)C(OC)=O)C1(C)C56C(C(C7(C)C(O)C6)=CC(OC7C8=COC=C8)=O)(O9)C3OC9(C(C)C)O5
**259**	$C_{37}H_{46}O_{12}$	682.2989	683.3068	R = OH. R_1 = Tg. R_2 = H. R_3 = $CH_3CHCH_2CH_3$. R_4 = H	OC12C3([H])C(OC(/C(C)=C/C)=O)C(C2)(C)C(C(O)C(OC)=O)C1(C)C45C(C(C6(C)C([H])C5)=CC(OC6C7=COC=C7)=O)(O8)C3OC8(C(C)CC)O4
**260**	$C_{39}H_{44}O_{12}$	704.2833	705.2911	R = OH. R_1 = Bz. R_2 = H. R_3 = $CH_3CHCH_2CH_3$. R_4 = H	OC12C3([H])C(OC(C4=CC=CC=C4)=O)C(C2)(C)C(C(O)C(OC)=O)C1(C)C56C(C(C7(C)C([H])C6)=CC(OC7C8=COC=C8)=O)(O9)C3OC9(C(C)CC)O5
**261**	$C_{39}H_{48}O_{14}$	740.3044	741.3122	R = OH. R_1 = Tg. R_2 = H. R_3 = $CH_3CHCH_2CH_3$. R_4 = OAc	OC12C3([H])C(OC(/C(C)=C/C)=O)C(C2)(C)C(C(O)C(OC)=O)C1(C)C45C(C(C6(C)C

					(OC(C)=O)C5)=CC(OC6C7=COC=C7)=O)(O8)C3OC8(C(C)CC)O4
**262**	$C_{41}H_{46}O_{14}$	762.2888	763.2966	R = OH. R_1 = Bz. R_2 = H. R_3 = $CH_3CHCH_2CH_3$. R_4 = OAc	OC12C3([H])C(OC(C4=CC=CC=C4)=O)C(C2)(C)C(C(O)C(OC)=O)C1(C)C56C(C(C7(C)C(OC(C)=O)C6)=CC(OC7C8=COC=C8)=O)(O9)C3OC9(C(C)CC)O5
**263**	$C_{37}H_{46}O_{13}$	698.2938	699.3017	R = OH. R_1 = Tg. R_2 = H. R_3 = $CH_3CHCH_2CH_3$. R_4 = OH	OC12C3([H])C(OC(/C(C)=C/C)=O)C(C2)(C)C(C(O)C(OC)=O)C1(C)C45C(C(C6(C)C(O)C5)=CC(OC6C7=COC=C7)=O)(O8)C3OC8(C(C)CC)O4
**264**	$C_{39}H_{44}O_{13}$	720.2782	721.2860	R = OH. R_1 = Bz. R_2 = H. R_3 = $CH_3CHCH_2CH_3$. R_4 = OH	OC12C3([H])C(OC(C4=CC=CC=C4)=O)C(C2)(C)C(C(O)C(OC)=O)C1(C)C56C(C(C7(C)C(O)C6)=CC(OC7C8=COC=C8)=O)(O9)C3OC9(C(C)CC)O5
**265**	$C_{37}H_{44}O_{12}$	680.2833	681.2911	R = OH. R_1 = Tg. R_2 = H. R_3 = (*E*)-CH_3C=$CHCH_3$. R_4 = H	OC12C3([H])C(OC(/C(C)=C/C)=O)C(C2)(C)C(C(O)C(OC)=O)C1(C)C45C(C(C6(C)C([H])C5)=CC(OC6C7=COC=C7)=O)(O8)C3OC8(/C(C)=C/C)O4
**266**	$C_{39}H_{42}O_{12}$	702.2676	703.2755	R = OH. R_1 = Bz. R_2 = H. R_3 = (*E*)-CH_3C=$CHCH_3$. R_4 = H	OC12C3([H])C(OC(C4=CC=CC=C4)=O)C(C2)(C)C(C(O)C(OC)=O)C1(C)C56C(C(C7(C)C([H])C6)=CC(OC7C8=COC=C8)=O)(O9)C3OC9(/C(C)=C/C)O5

**267**	$C_{39}H_{46}O_{14}$	738.2888	739.2966	R = OH. R_1 = Tg. R_2 = H. R_3 = (*E*)-CH_3C=$CHCH_3$. R_4 = OAc	OC12C3([H])C(OC(/C(C)=C/C)=O)C(C2)(C)C(C(O)C(OC)=O)C1(C)C45C(C(C6(C)C(OC(C)=O)C5)=CC(OC6C7=COC=C7)=O)(O8)C3OC8(/C(C)=C/C)O4
**268**	$C_{41}H_{44}O_{14}$	760.2731	761.2809	R = OH. R_1 = Bz. R_2 = H. R_3 = (*E*)-CH_3C=$CHCH_3$. R_4 = OAc	OC12C3([H])C(OC(C4=CC=CC=C4)=O)C(C2)(C)C(C(O)C(OC)=O)C1(C)C56C(C(C7(C)C(OC(C)=O)C6)=CC(OC7C8=COC=C8)=O)(O9)C3OC9(/C(C)=C/C)O5
**269**	$C_{37}H_{44}O_{13}$	696.2782	697.2860	R = OH. R_1 = Tg. R_2 = H. R_3 = (*E*)-CH_3C=$CHCH_3$. R_4 = OH	OC12C3([H])C(OC(/C(C)=C/C)=O)C(C2)(C)C(C(O)C(OC)=O)C1(C)C45C(C(C6(C)C(O)C5)=CC(OC6C7=COC=C7)=O)(O8)C3OC8(/C(C)=C/C)O4
**270**	$C_{39}H_{42}O_{13}$	718.2625	719.2704	R = OH. R_1 = Bz. R_2 = H. R_3 = (*E*)-CH_3C=$CHCH_3$. R_4 = OH	OC12C3([H])C(OC(C4=CC=CC=C4)=O)C(C2)(C)C(C(O)C(OC)=O)C1(C)C56C(C(C7(C)C(O)C6)=CC(OC7C8=COC=C8)=O)(O9)C3OC9(/C(C)=C/C)O5

[1]Substitution sites based on the structure of phragmalin-type limonoids with an 8,9,30-*ortho*-ester unit reported on literature.

Table S3. General fragmentation reactions observed in phragmalin-limonoid type.

Characteristic groups	Neutral loss	ESI+ fragment mass
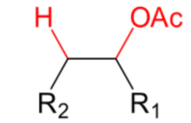	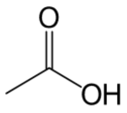 $C_2H_4O_2$ (60 Da)	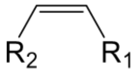 F^+=MW + 1 - $C_2H_4O_2$
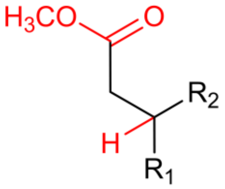	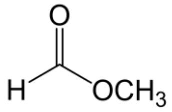 $C_2H_4O_2$ (60 Da)	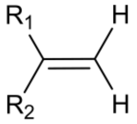 F^+=MW + 1 - $C_2H_4O_2$
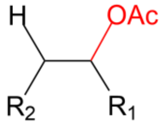	$H_2C{=}C{=}O$ C_2H_2O (42 Da)	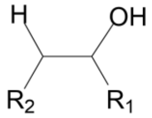 F^+=MW + 1 - C_2H_2O
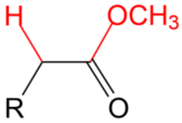	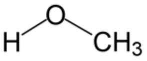 CH_4O (32 Da)	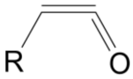 F^+=MW + 1 - CH_4O
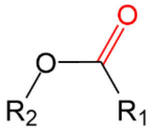	$C{\equiv}O$ CO (28 Da)	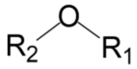 F^+=MW + 1 - CO
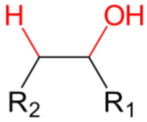	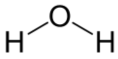 H_2O (18 Da)	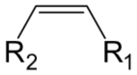 F^+=MW + 1 - H_2O
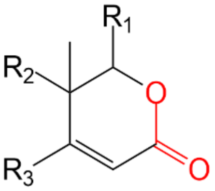	$O{=}C{=}O$ CO_2 (44 Da)	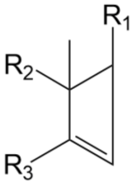 F^+=MW + 1 - CO_2
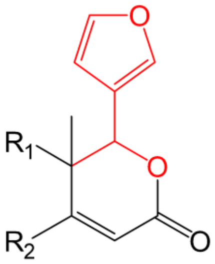	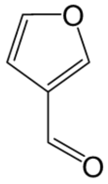 $C_5H_4O_2$ (96 Da) RDA	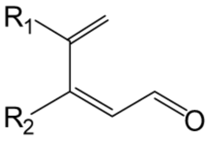 F^+=MW + 1 - $C_5H_4O_2$

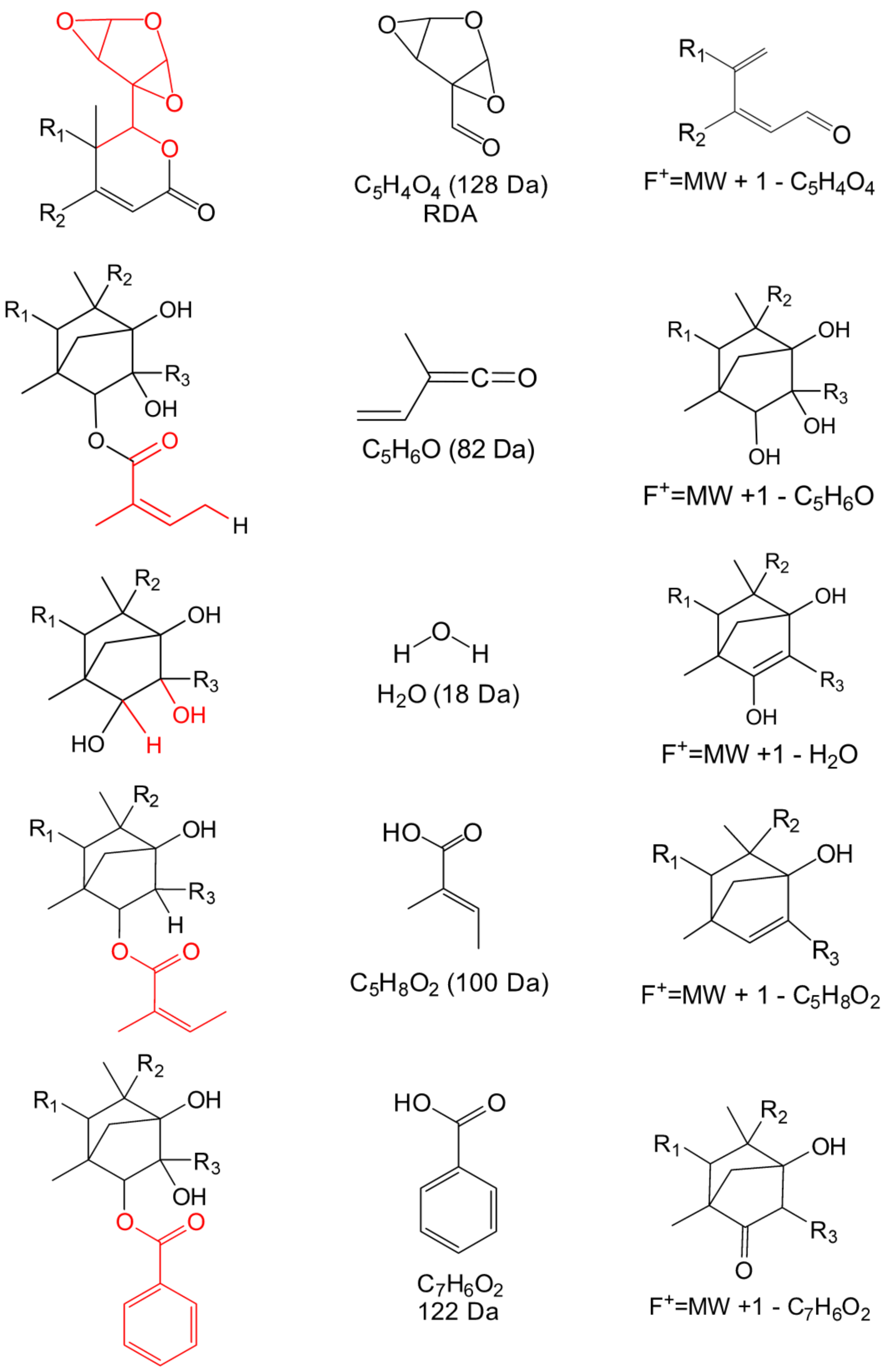
C5H4O4 (128 Da)
RDA
F+=MW + 1 - C5H4O4
C5H6O (82 Da)
F+=MW +1 - C5H6O
H2O (18 Da)
F+=MW +1 - H2O
C5H8O2 (100 Da)
F+=MW + 1 - C5H8O2
C7H6O2
122 Da
F+=MW +1 - C7H6O2

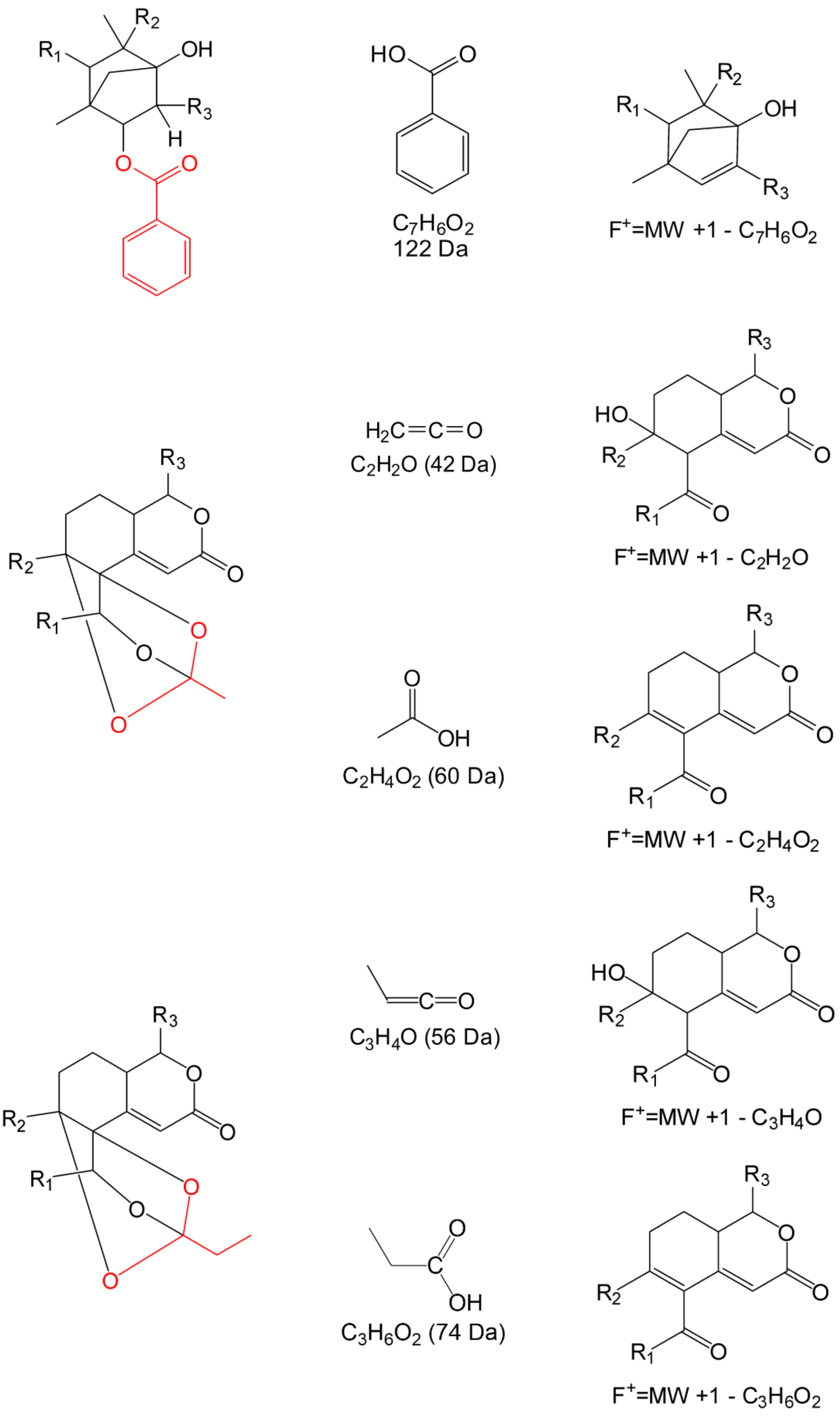
R1
R2
OH
R3
H
O
O
HO
O
C7H6O2
122 Da
F+=MW +1 - C7H6O2
H2C=C=O
C2H2O (42 Da)
F+=MW +1 - C2H2O
C2H4O2 (60 Da)
F+=MW +1 - C2H4O2
C=O
C3H4O (56 Da)
F+=MW +1 - C3H4O
C
OH
C3H6O2 (74 Da)
F+=MW +1 - C3H6O2

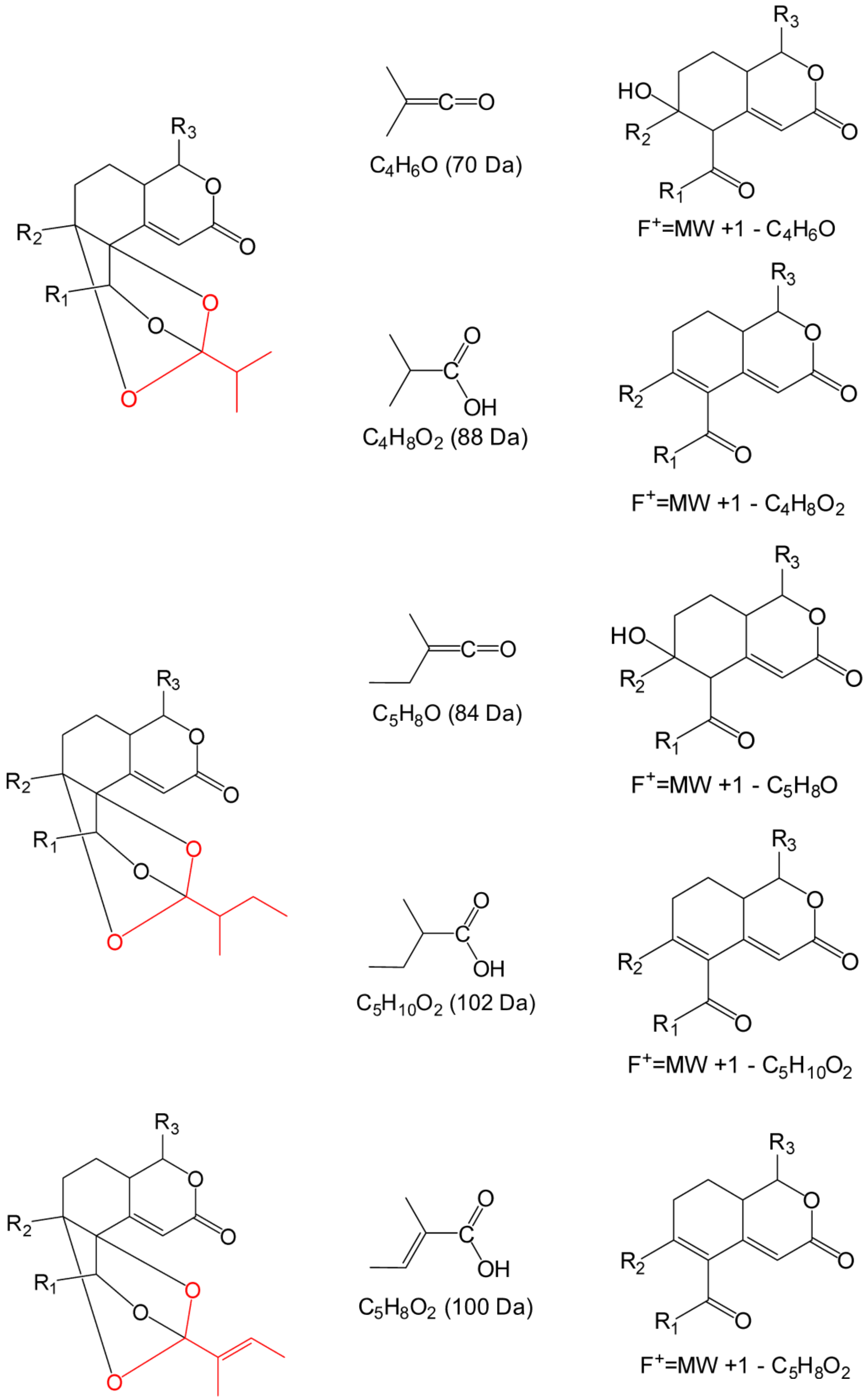

Legend: Red color corresponds to the main neutral losses that occur in the limonoids described in this work; F^+: Fragment ion; MW: Molecular weight; $F^+ = MW + 1 - MF$ (Molecular formula): Equation corresponds to

the fragmentation of the molecule with an additional proton (MW + 1), resulting in the formation of the fragment ion.

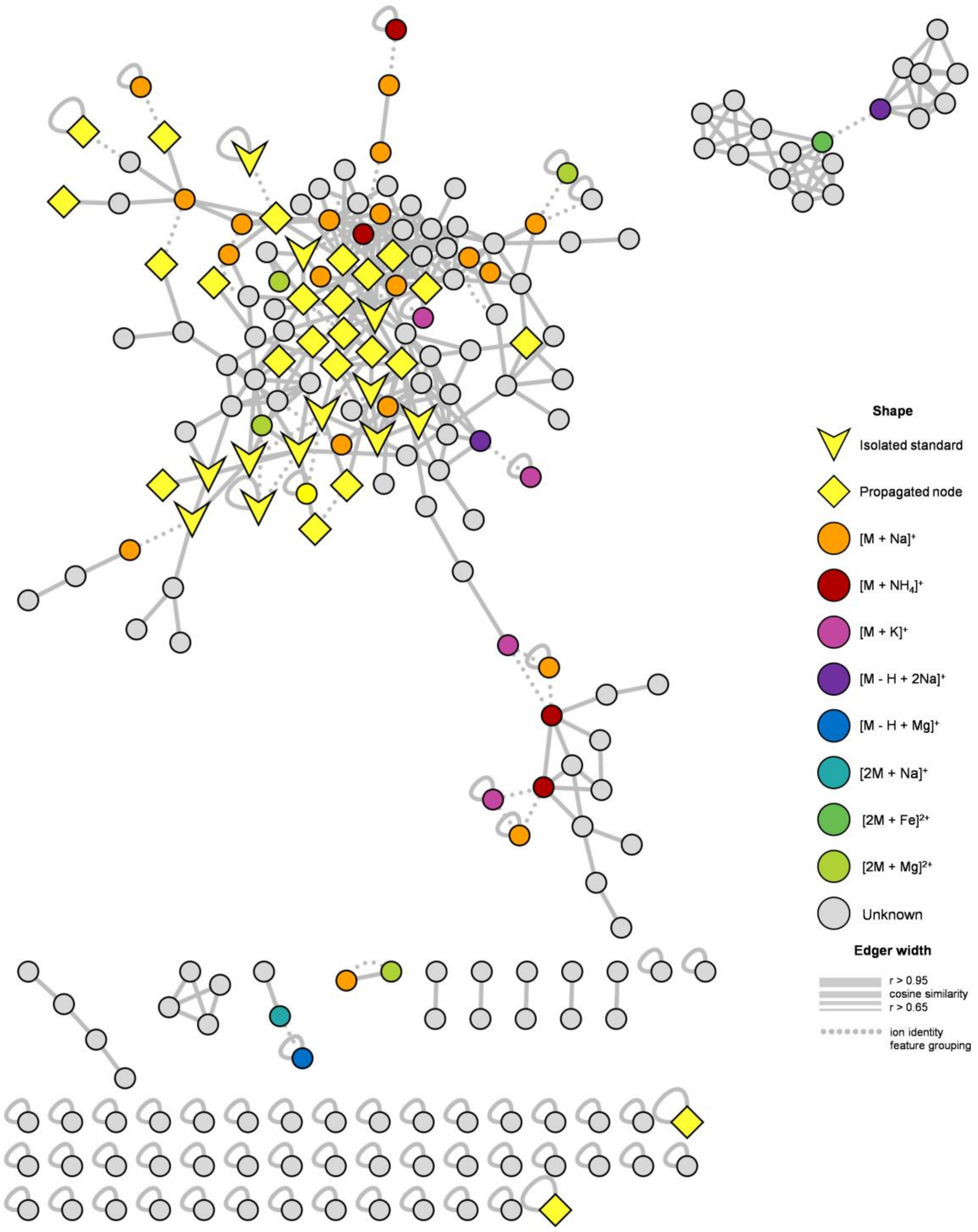

Figure S1. Molecular network from *Swietenia macrophylla* G. King dichloromethane extract (DCMEt) using UPLC-HRMS in positive ionization mode ESI+ (https://gnps.ucsd.edu/ProteoSAFe/status.jsp?task=8b7d836ad2694de0b932cf65db8c3fae). The network consist of 218 molecular features and the dataset be available in MassIVE CCMS MSV000087547 (https://massive.ucsd.edu/ProteoSAFe/dataset.jsp?task=f6307135be7c416584e8327d36c07146).

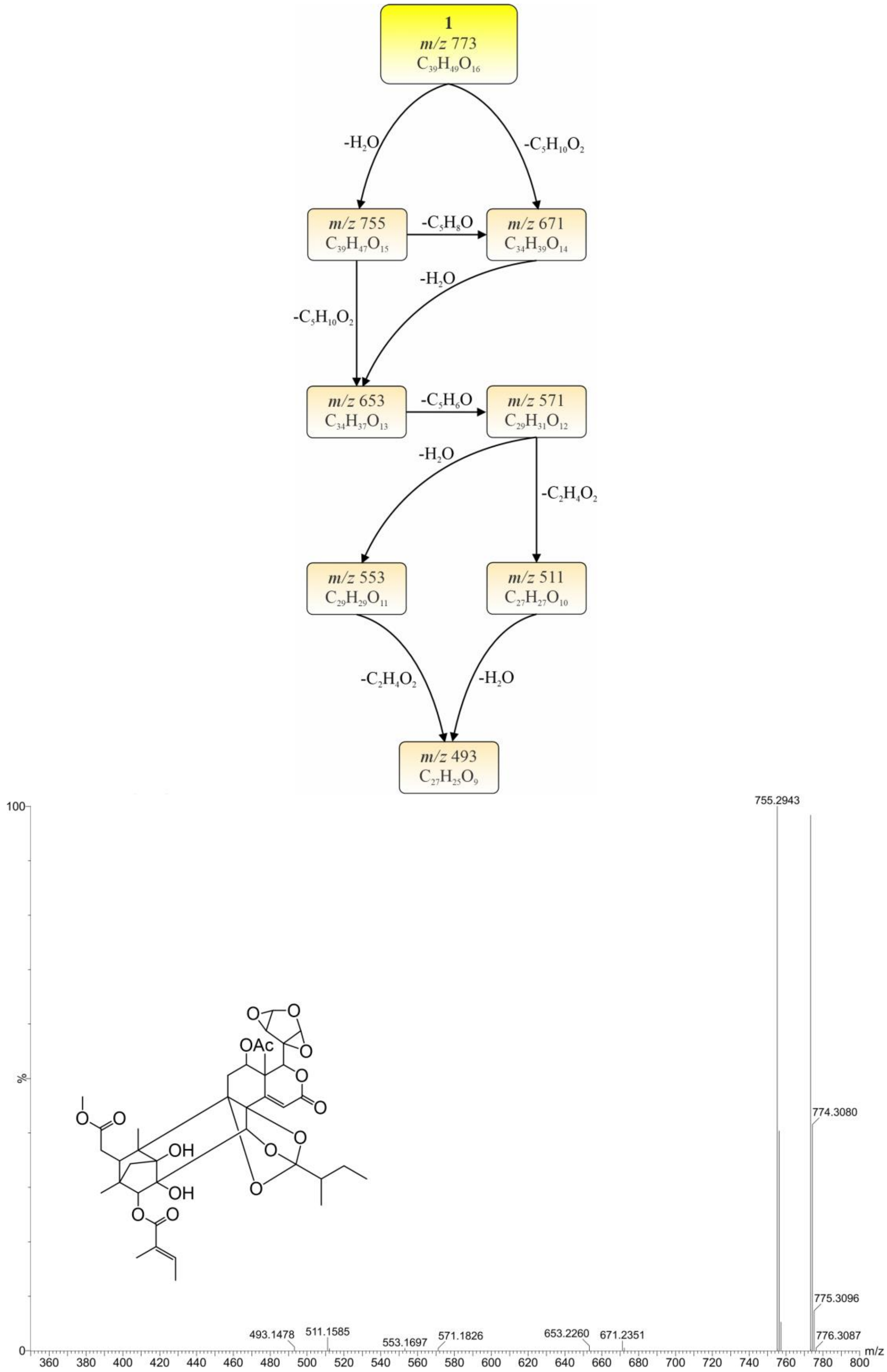

Figure S2. Fragmentation pattern of limonoid **1** computed from tandem MS data.

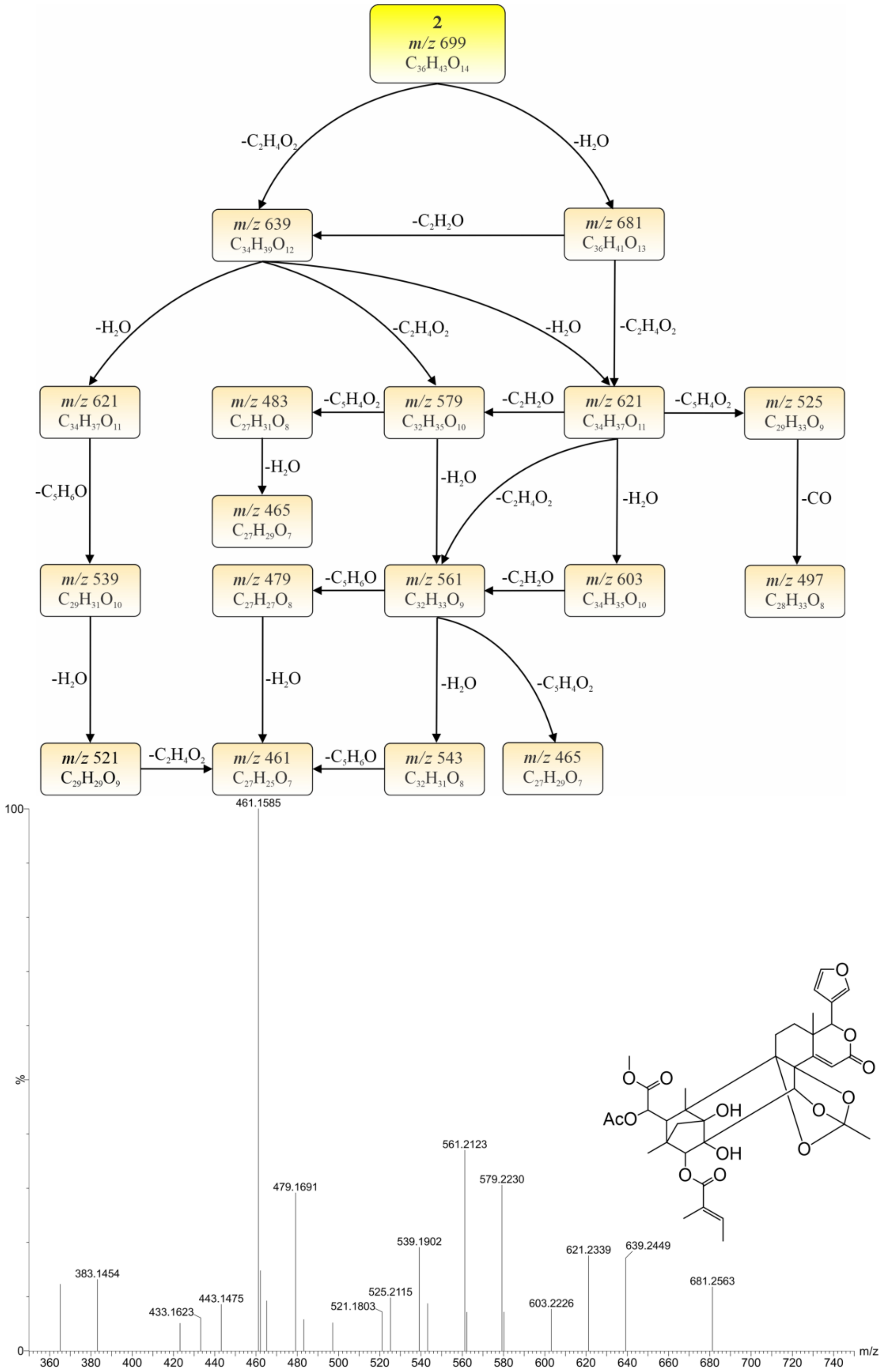

Figure S3. Fragmentation pattern of limonoid **2** computed from tandem MS data.

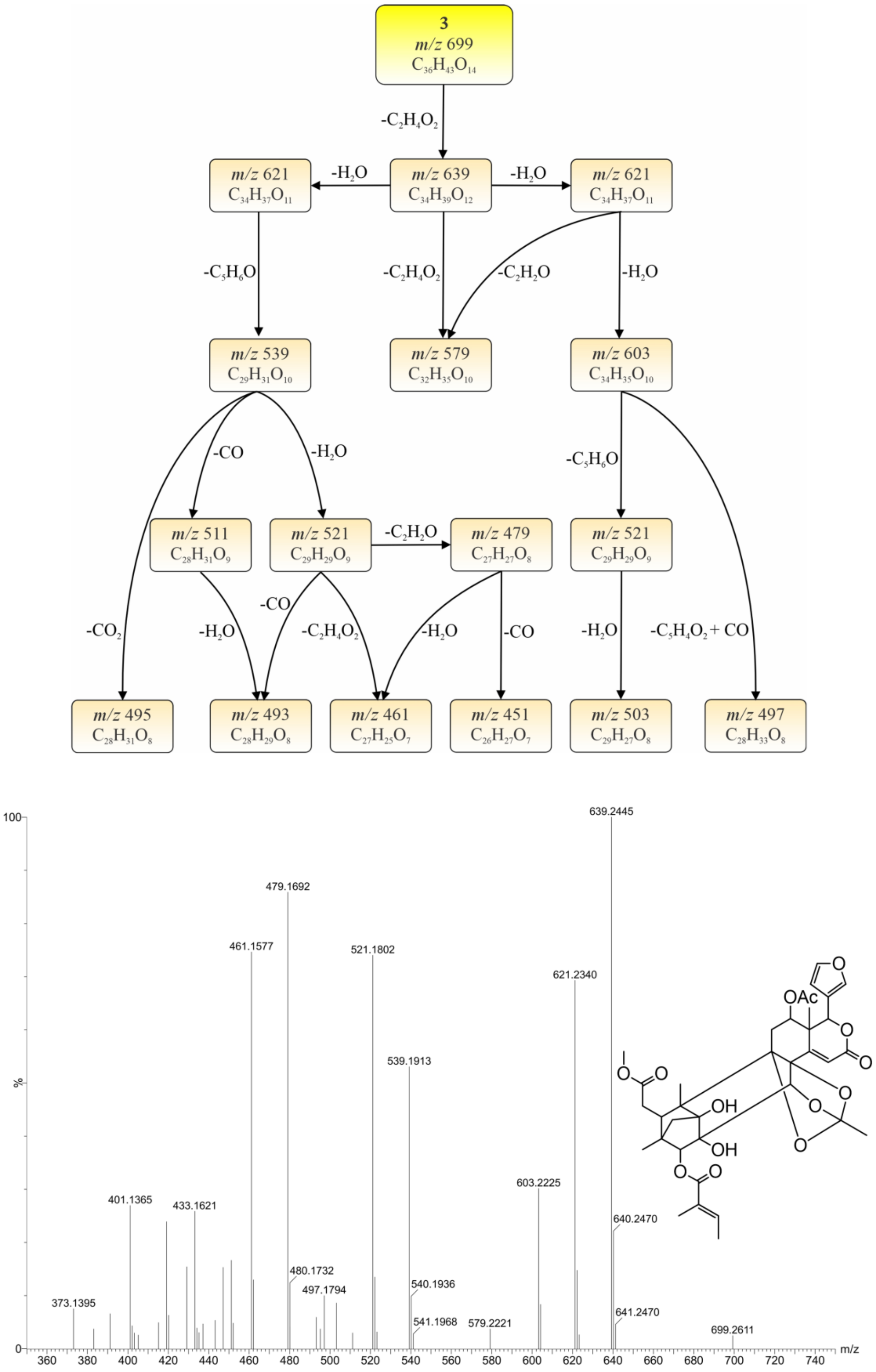

Figure S4. Fragmentation pattern of limonoid **3** computed from tandem MS data.

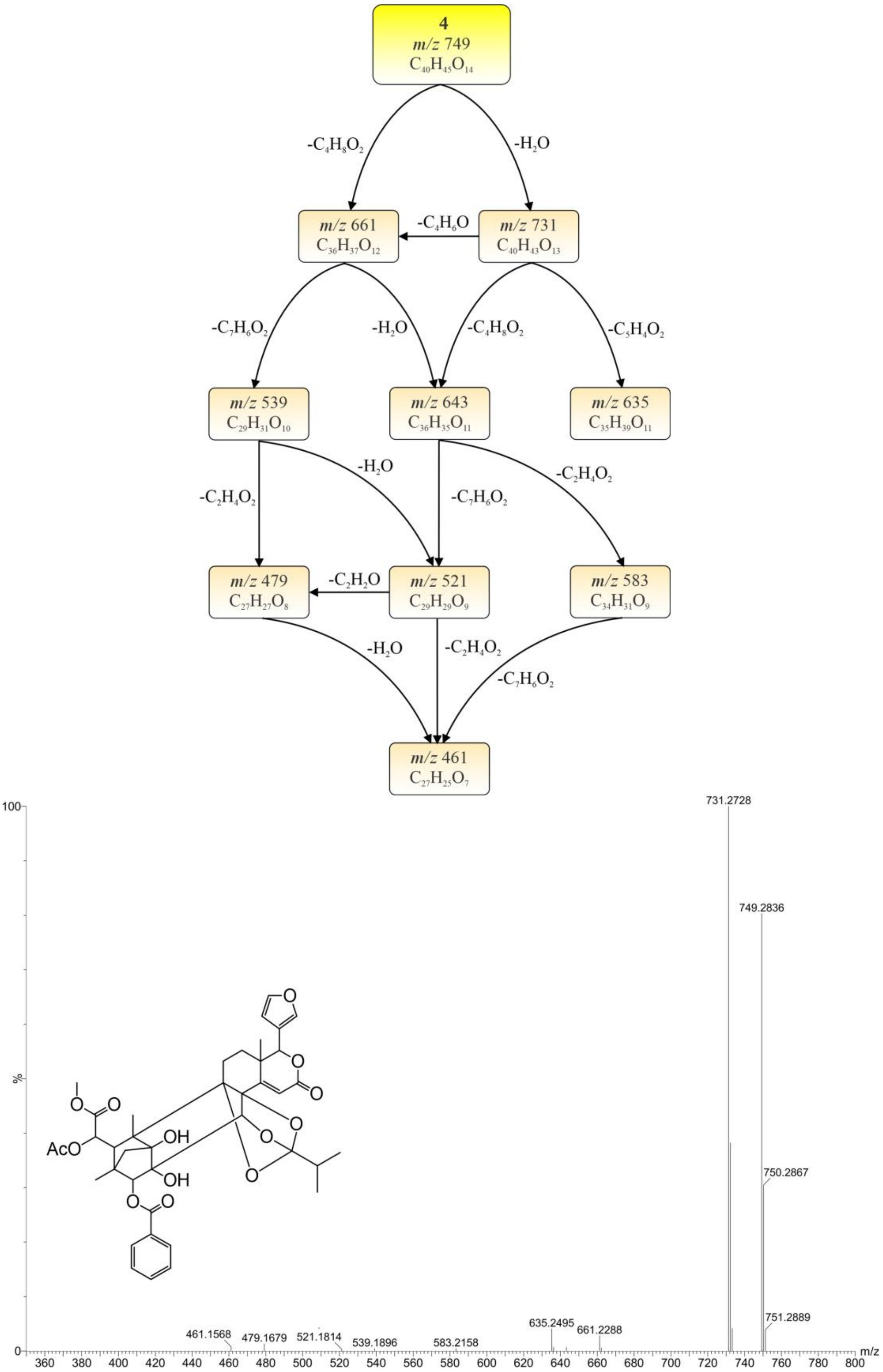

Figure S5. Fragmentation pattern of limonoid **4** computed from tandem MS data.

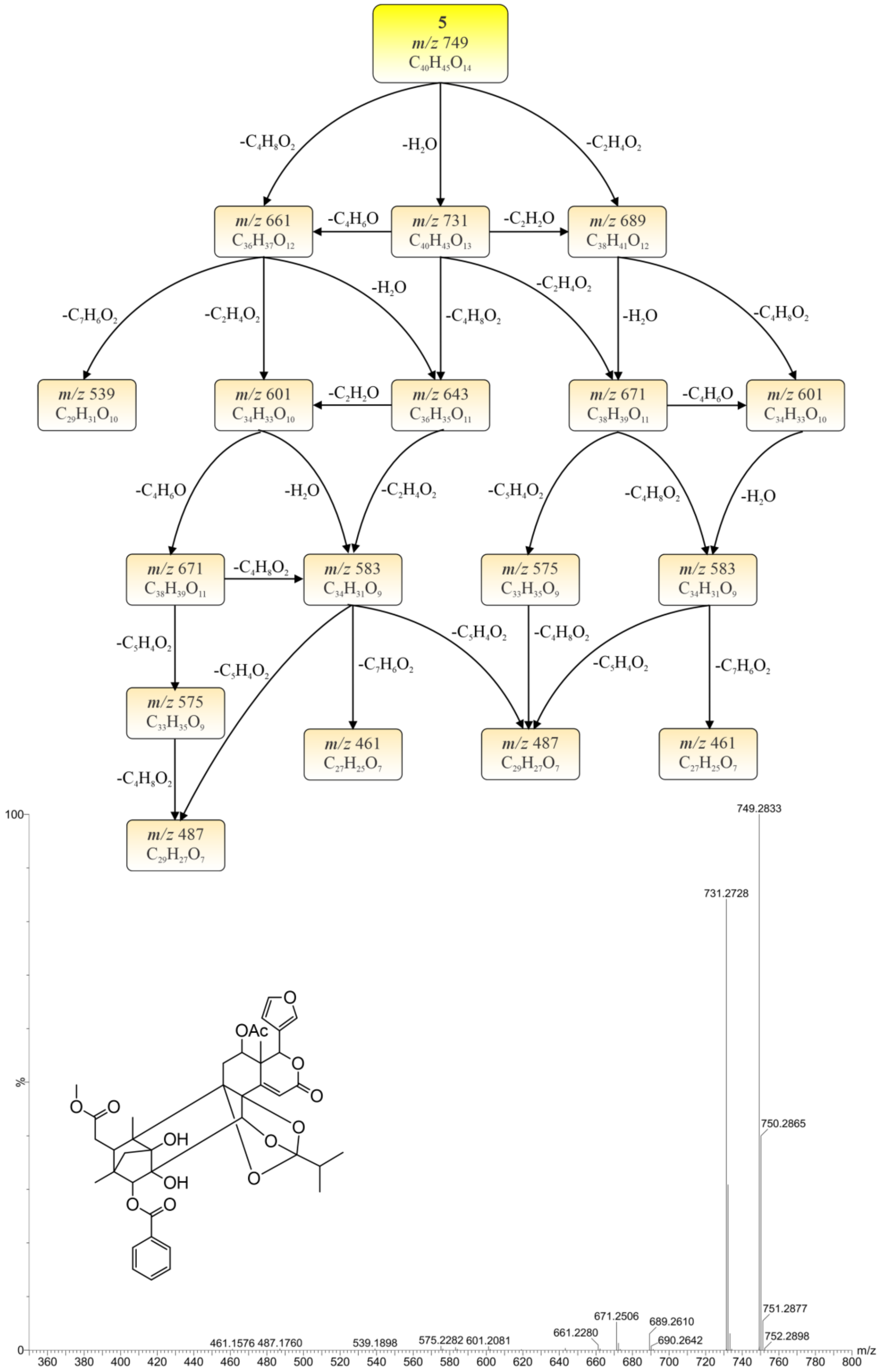

Figure S6. Fragmentation pattern of limonoid **5** computed from tandem MS data.

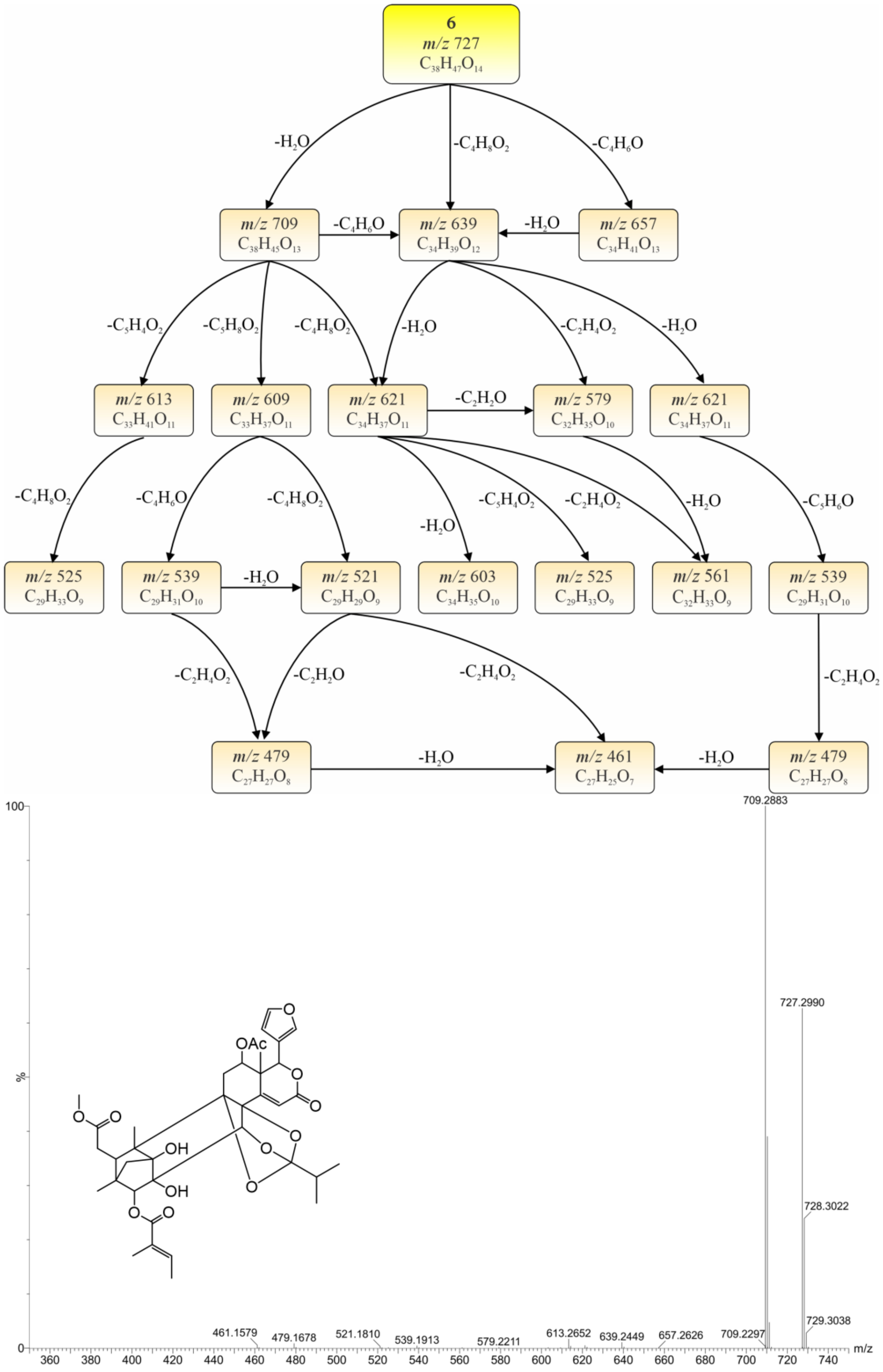

Figure S7. Fragmentation pattern of limonoid **6** computed from tandem MS data.

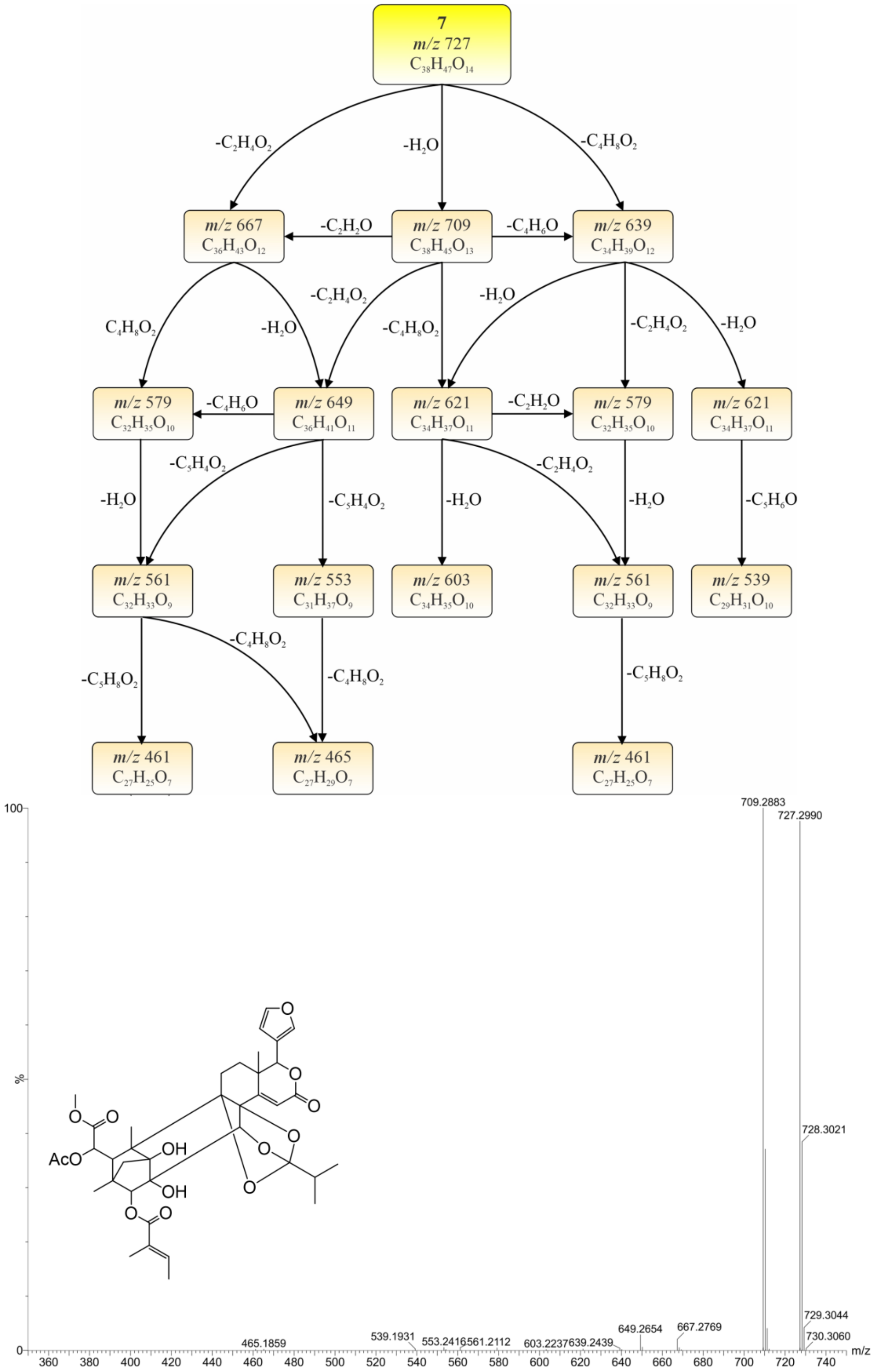

Figure S8. Fragmentation pattern of limonoid **7** computed from tandem MS data.

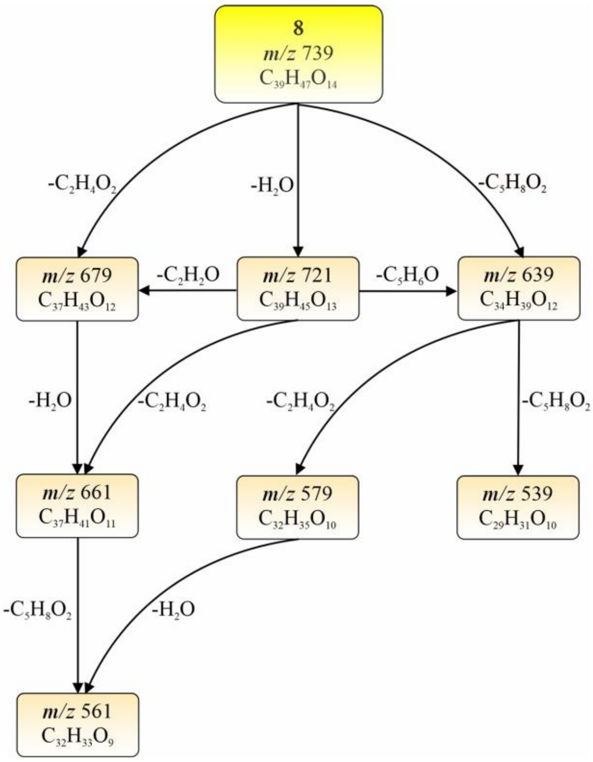

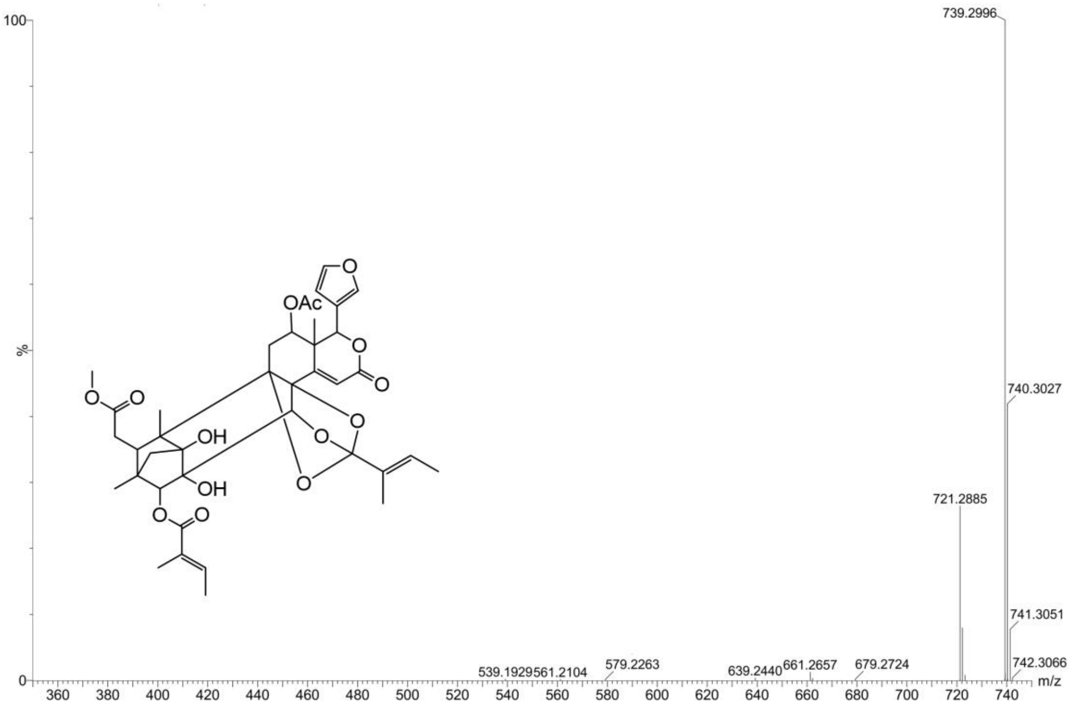

Figure S9. Fragmentation pattern of limonoid **8** computed from tandem MS data.

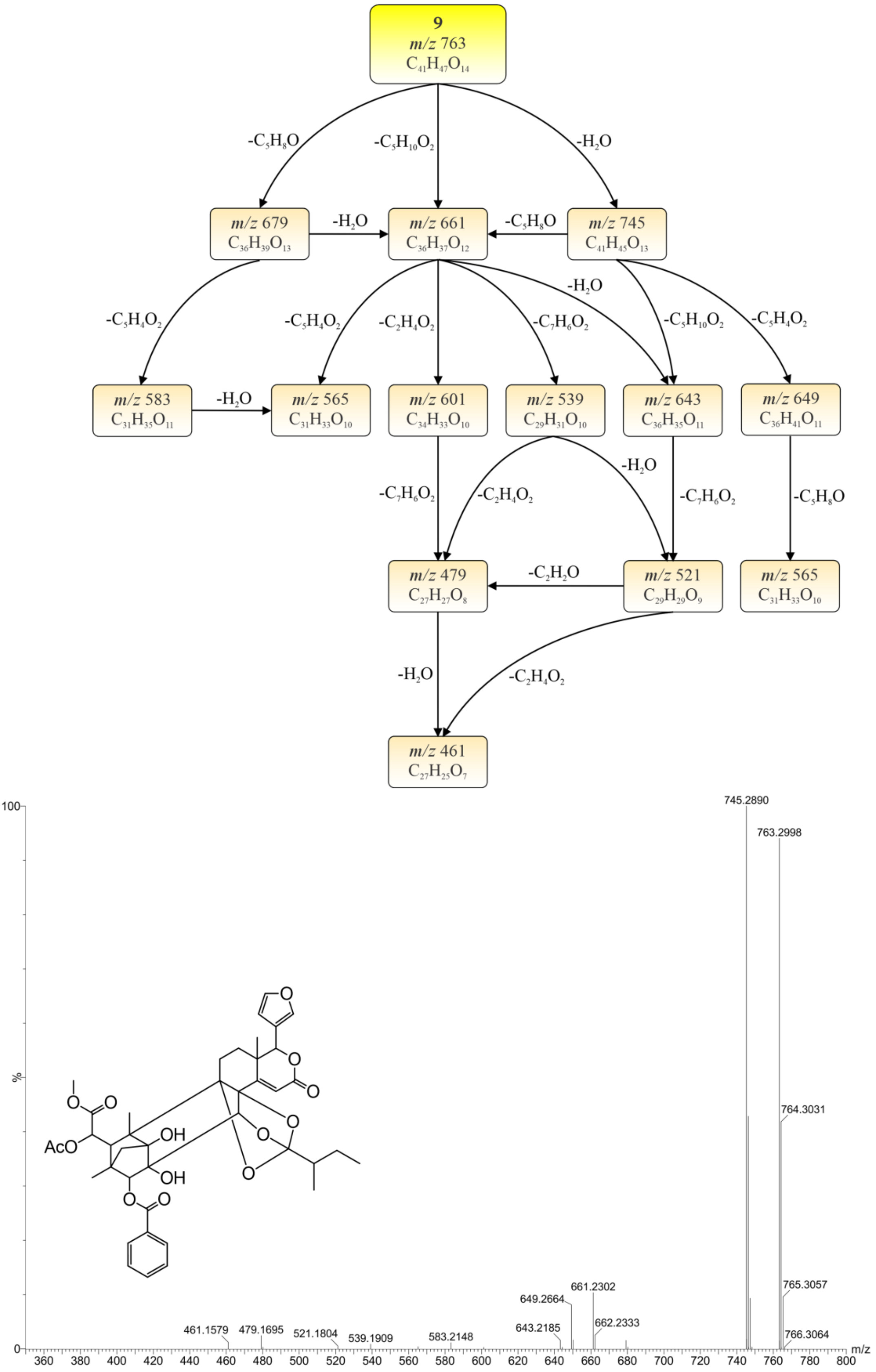

Figure S10. Fragmentation pattern of limonoid **9** computed from tandem MS data.

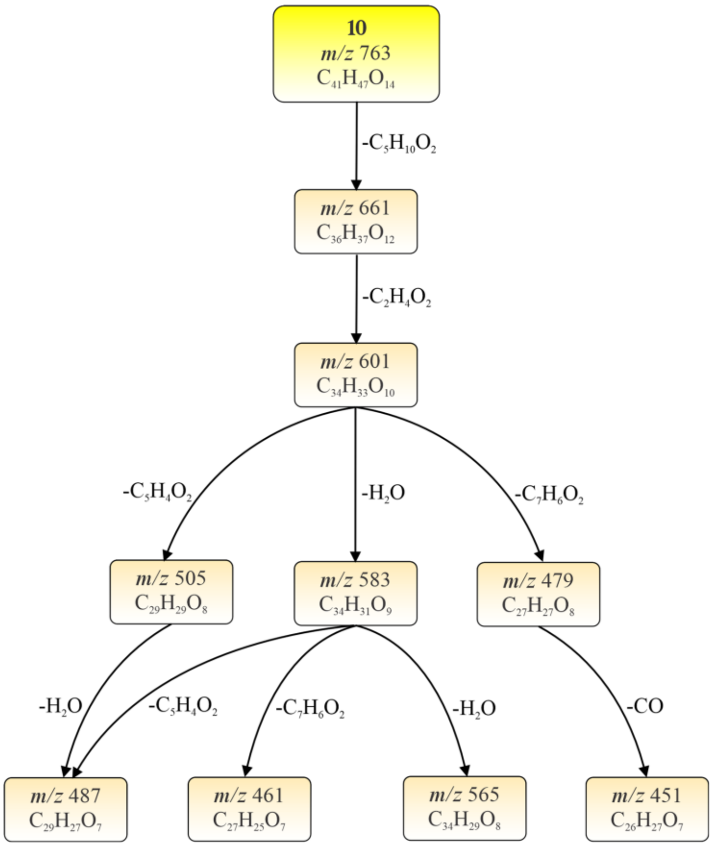

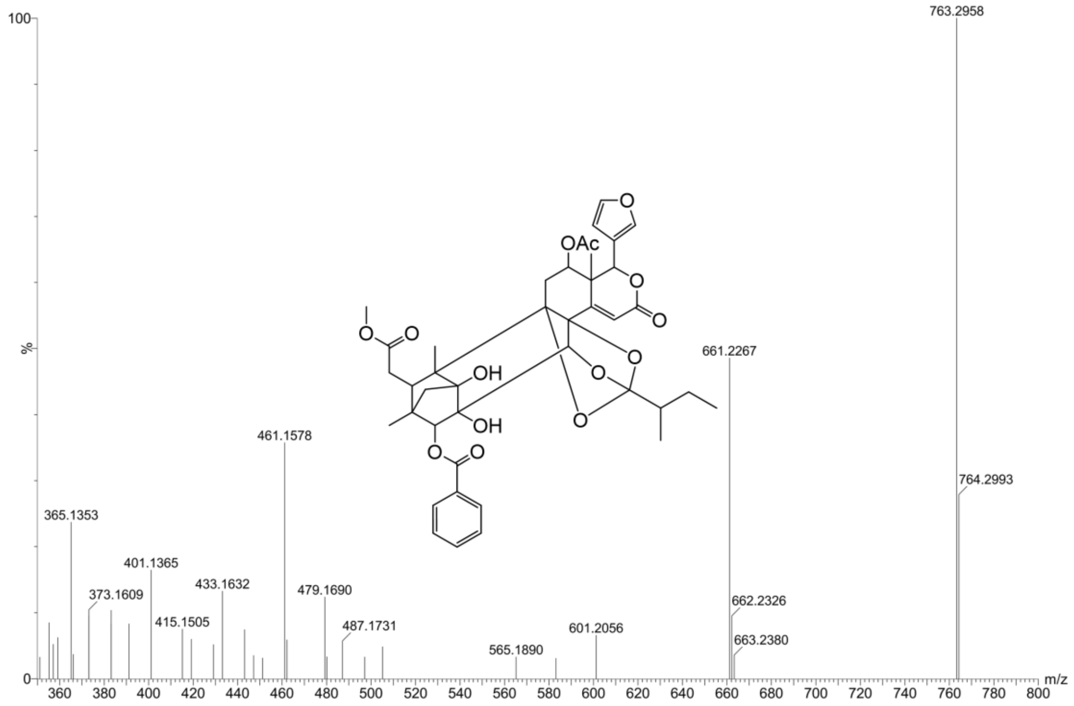

Figure S11. Fragmentation pattern of limonoid **10** computed from tandem MS data.

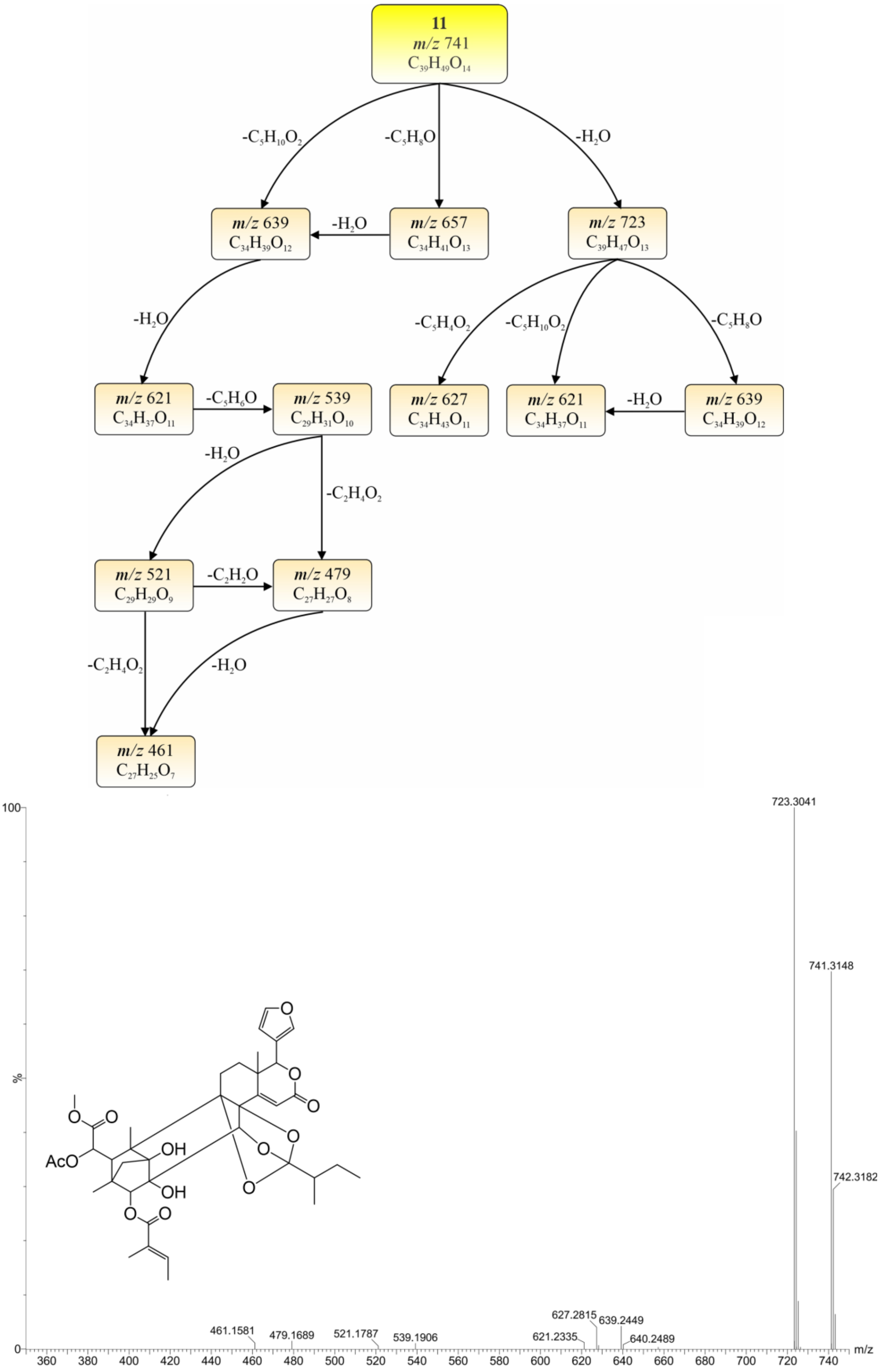

Figure S12. Fragmentation pattern of limonoid **11** computed from tandem MS data.

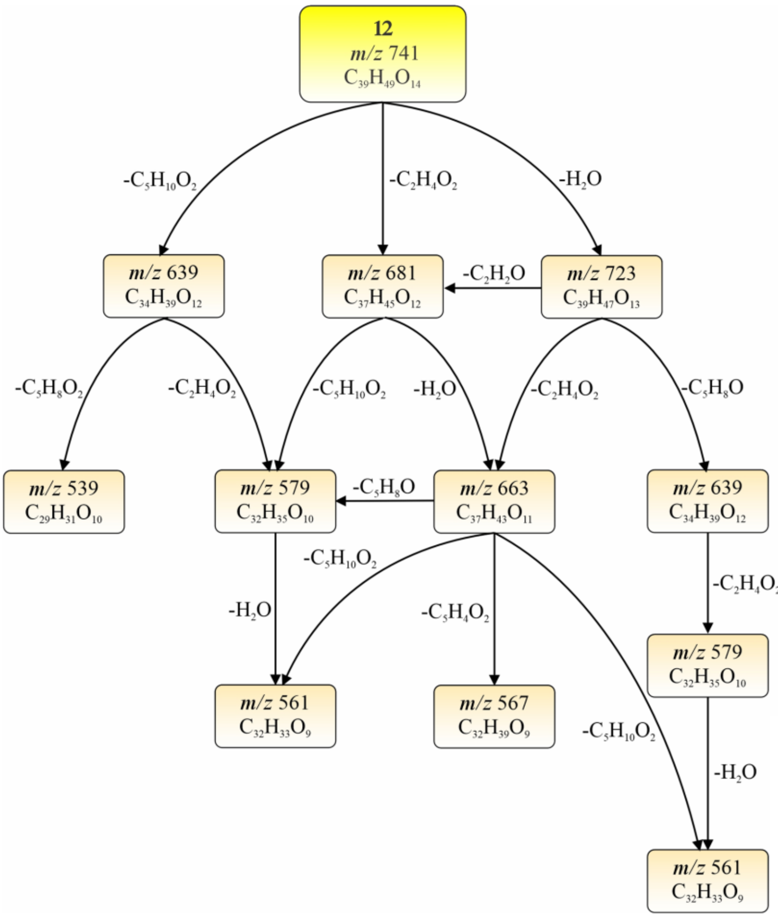

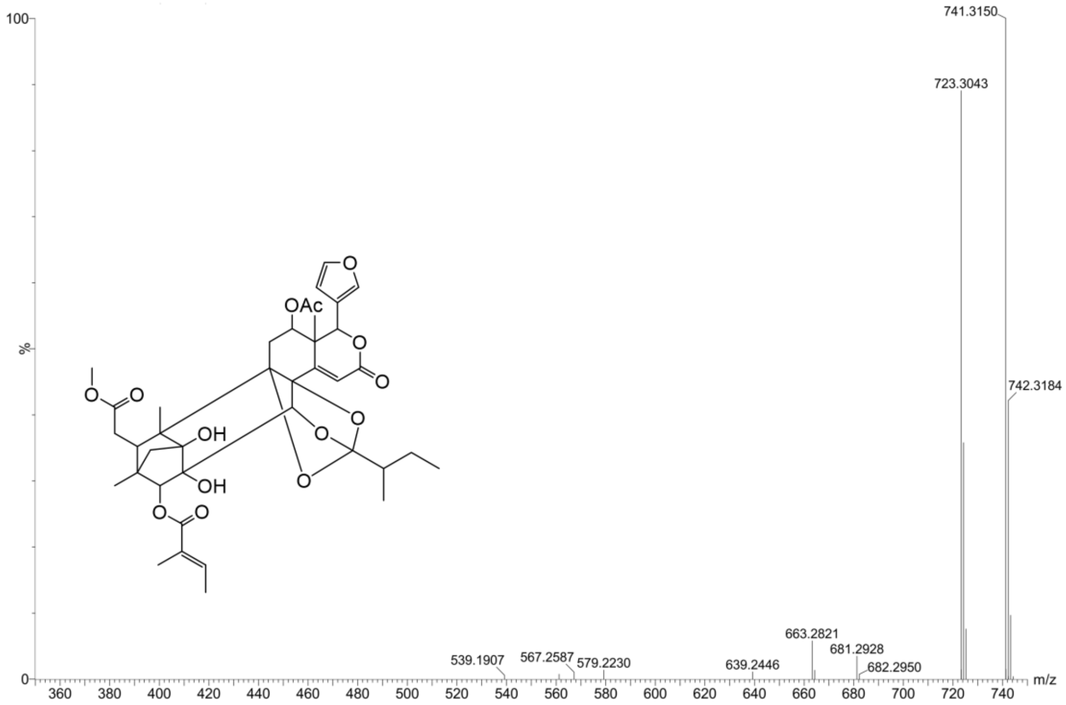

Figure S13. Fragmentation pattern of limonoid **12** computed from tandem MS data.

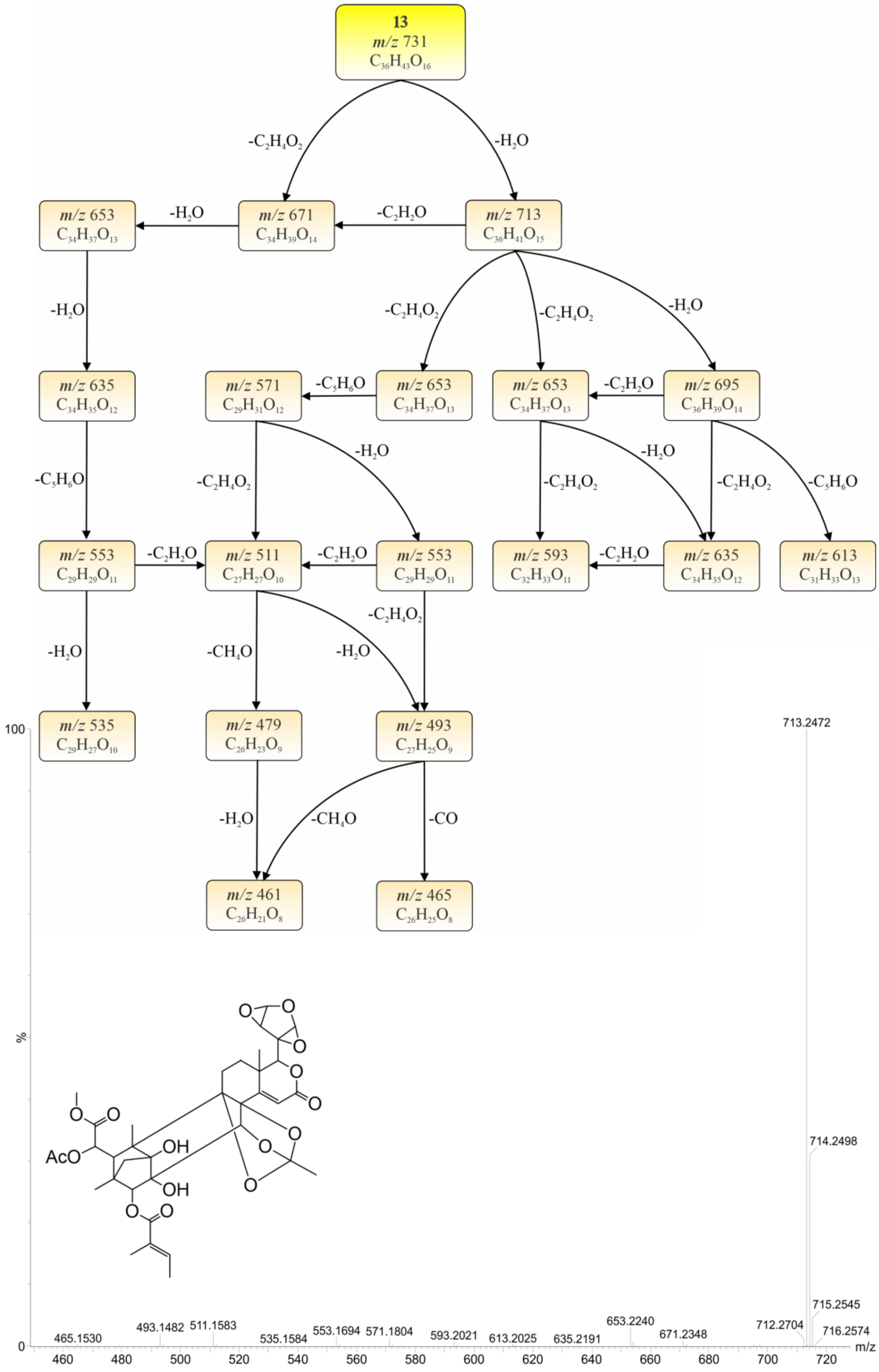

Figure S14. Fragmentation pattern of limonoid **13** computed from tandem MS data.

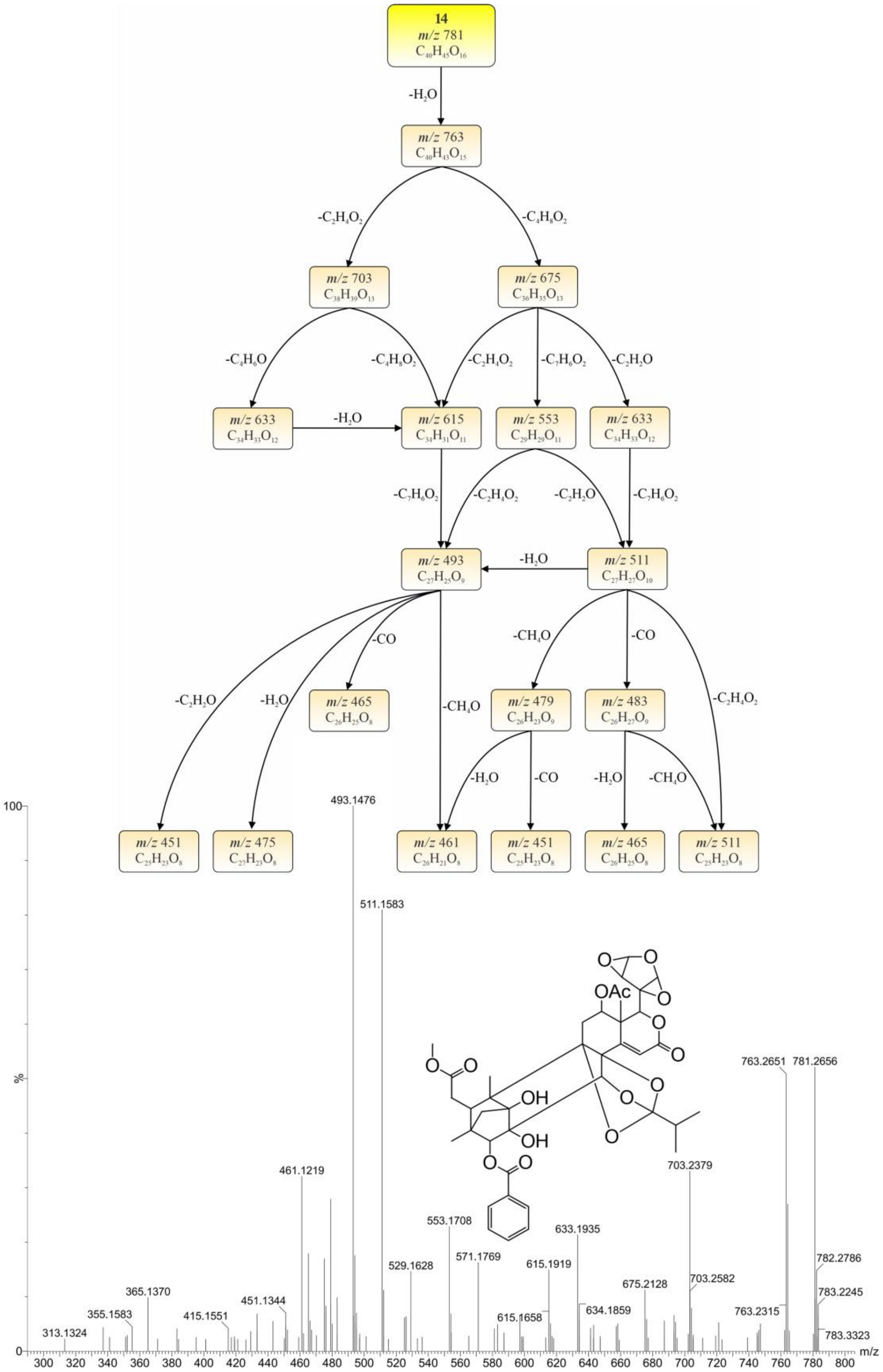

Figure S15. Fragmentation pattern of limonoid **14** computed from tandem MS data.

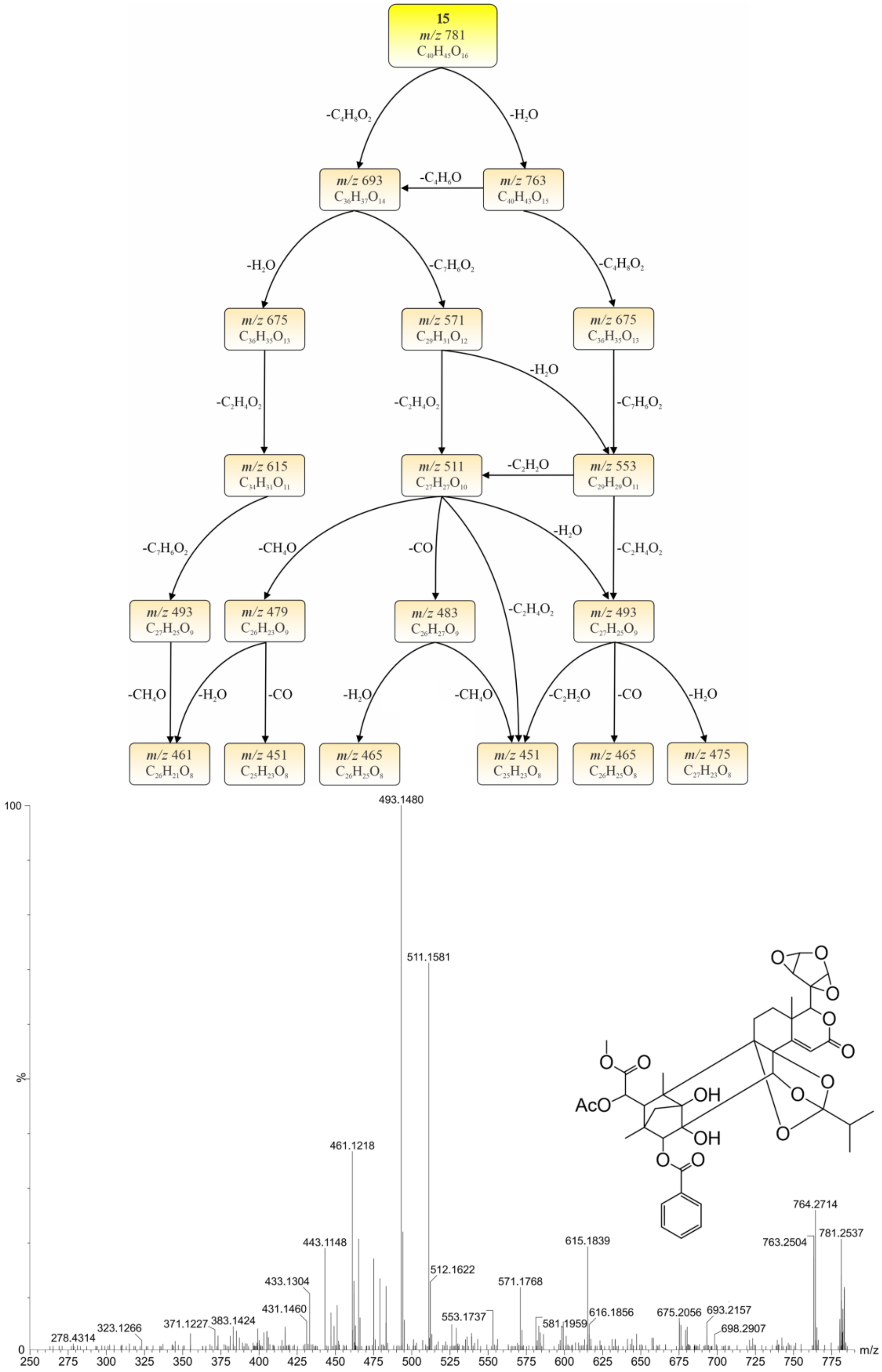

Figure S16. Fragmentation pattern of limonoid **15** computed from tandem MS data.

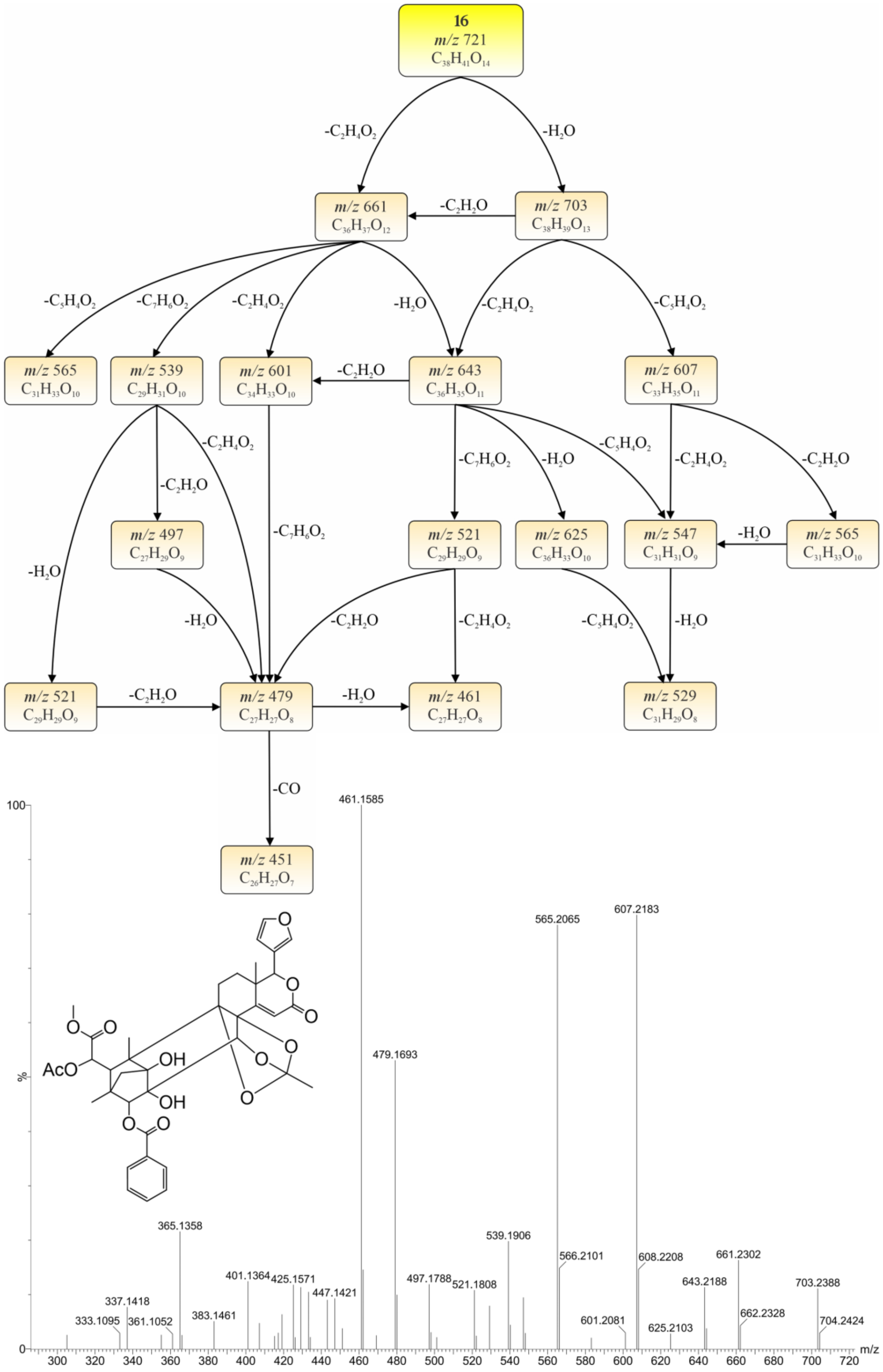

Figure S17. Fragmentation pattern of limonoid **16** computed from tandem MS data.

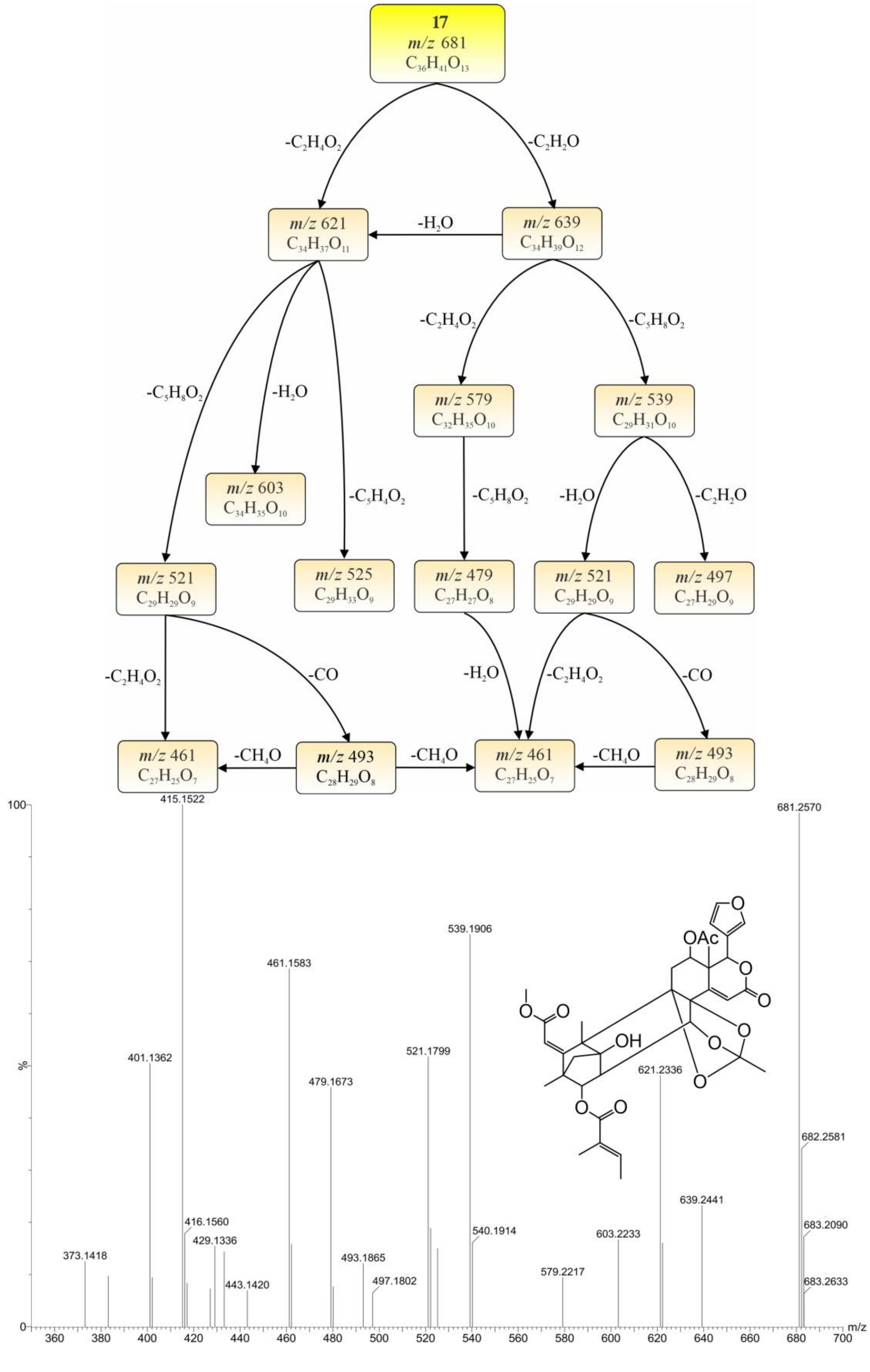

Figure S18. Fragmentation pattern of limonoid **17** computed from tandem MS data.

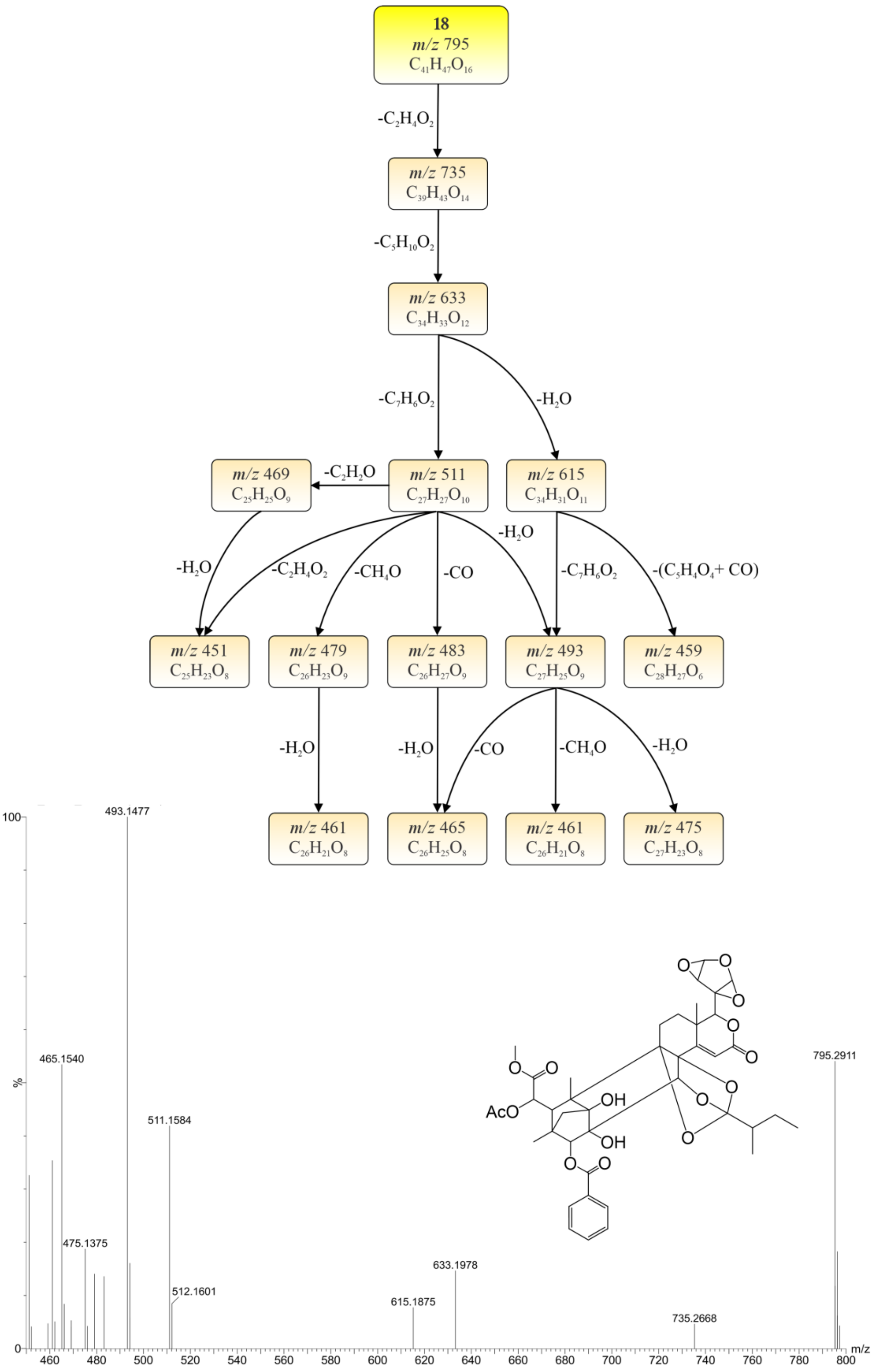

Figure S19. Fragmentation pattern of limonoid **18** computed from tandem MS data.

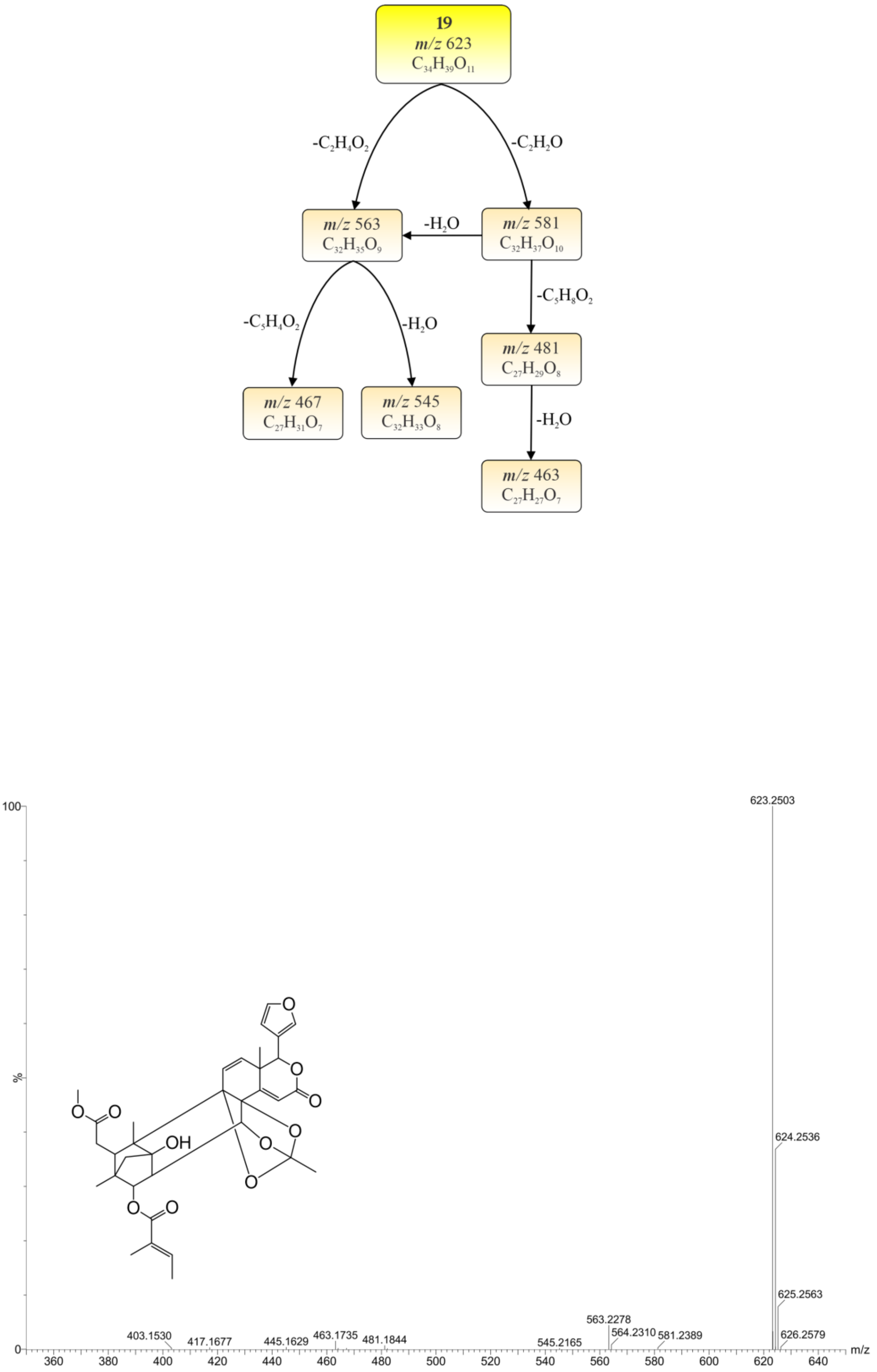

Figure S20. Fragmentation pattern of limonoid **19** computed from tandem MS data.

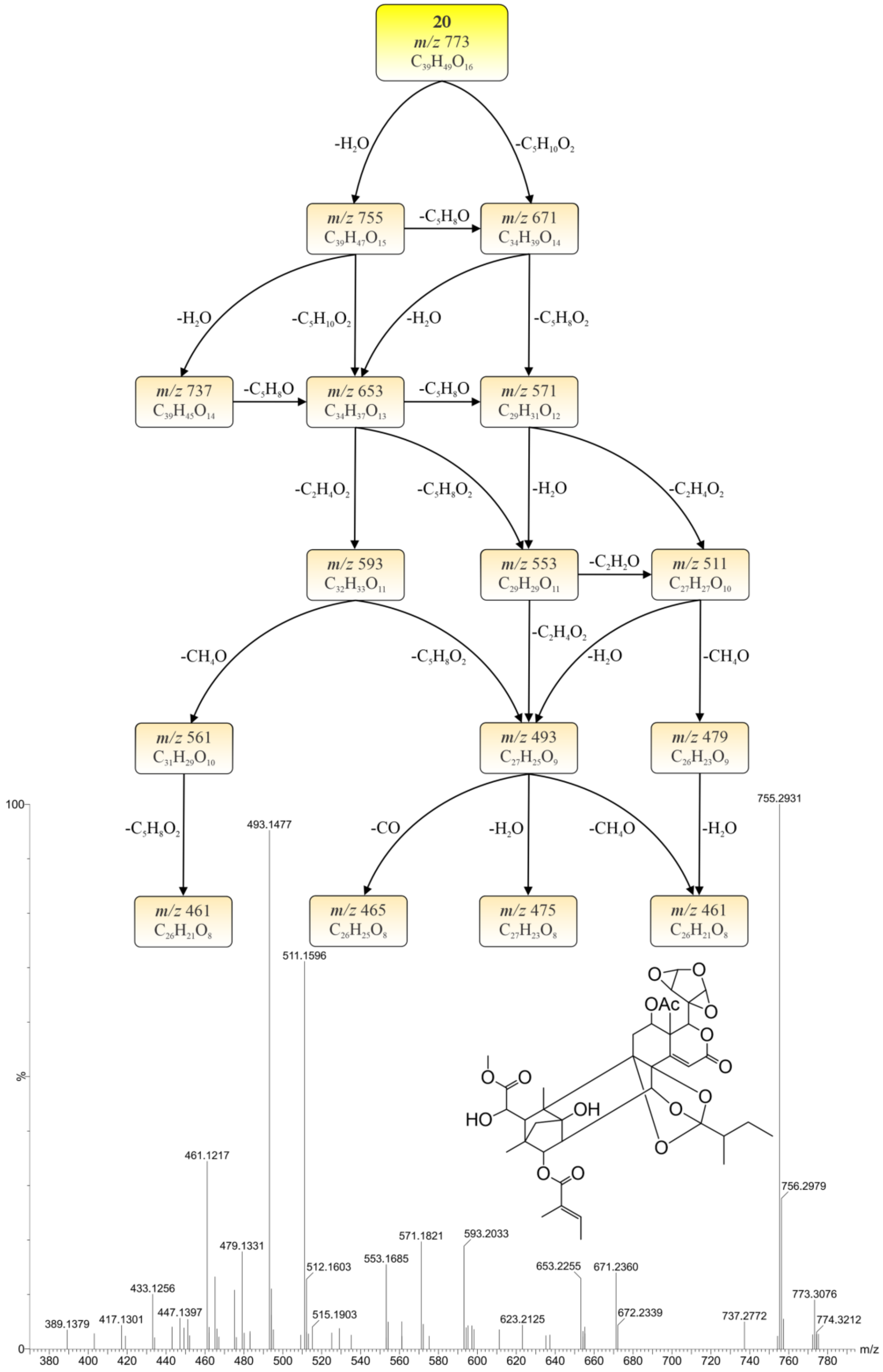

Figure S21. Fragmentation pattern of limonoid **20** computed from tandem MS data.

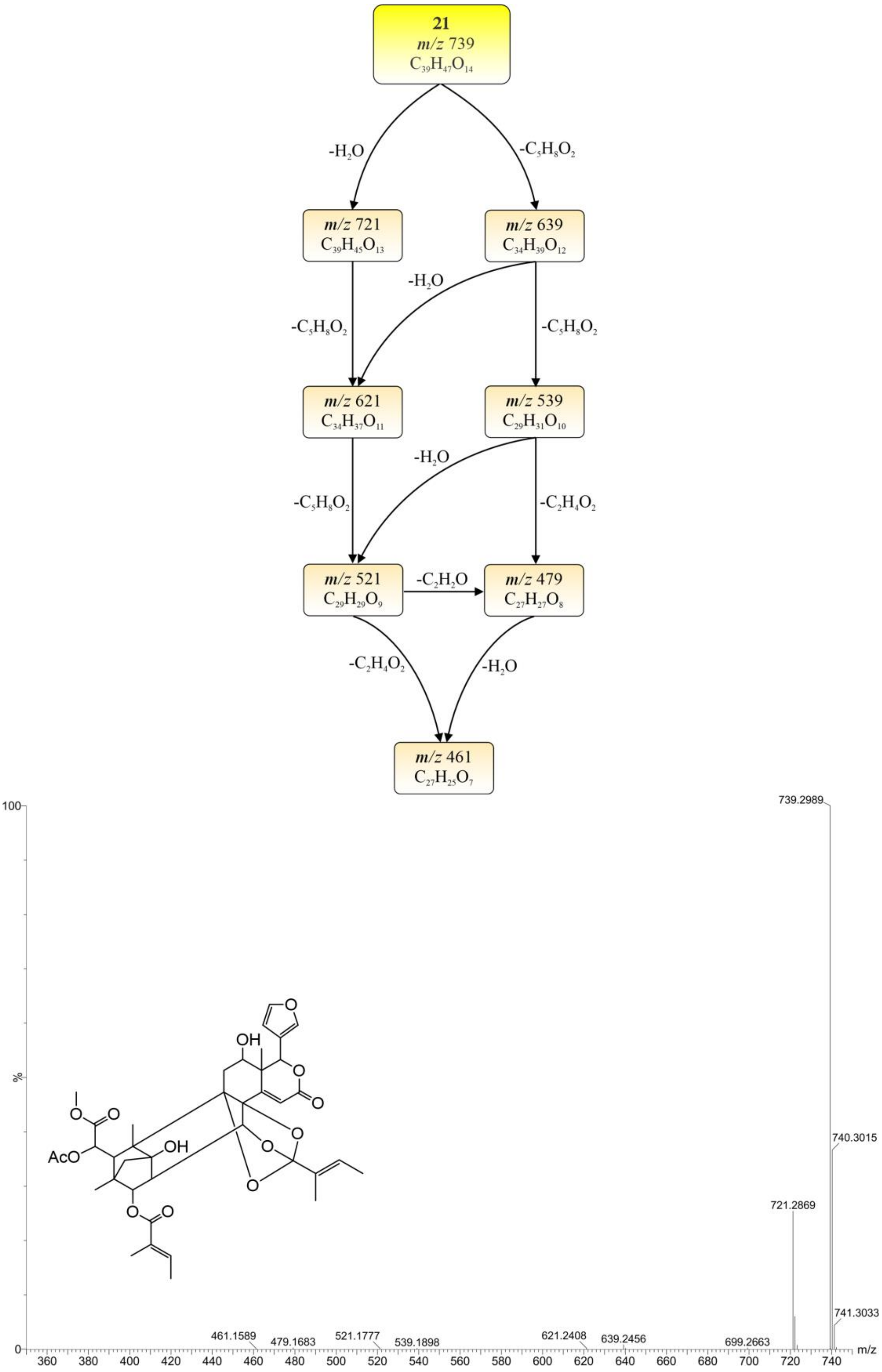

Figure S22. Fragmentation pattern of limonoid **21** computed from tandem MS data.

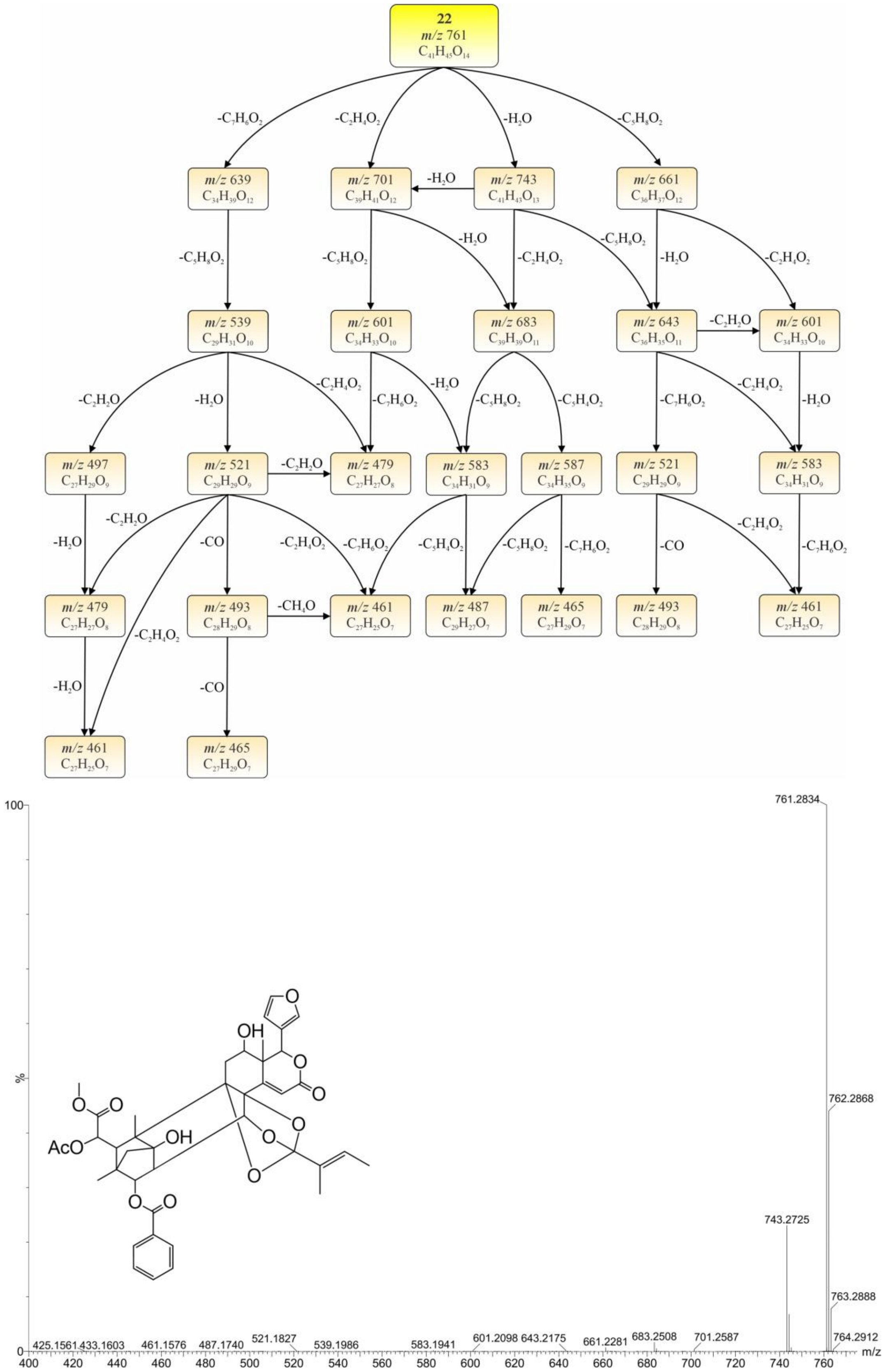

Figure S23. Fragmentation pattern of limonoid **22** computed from tandem MS data.

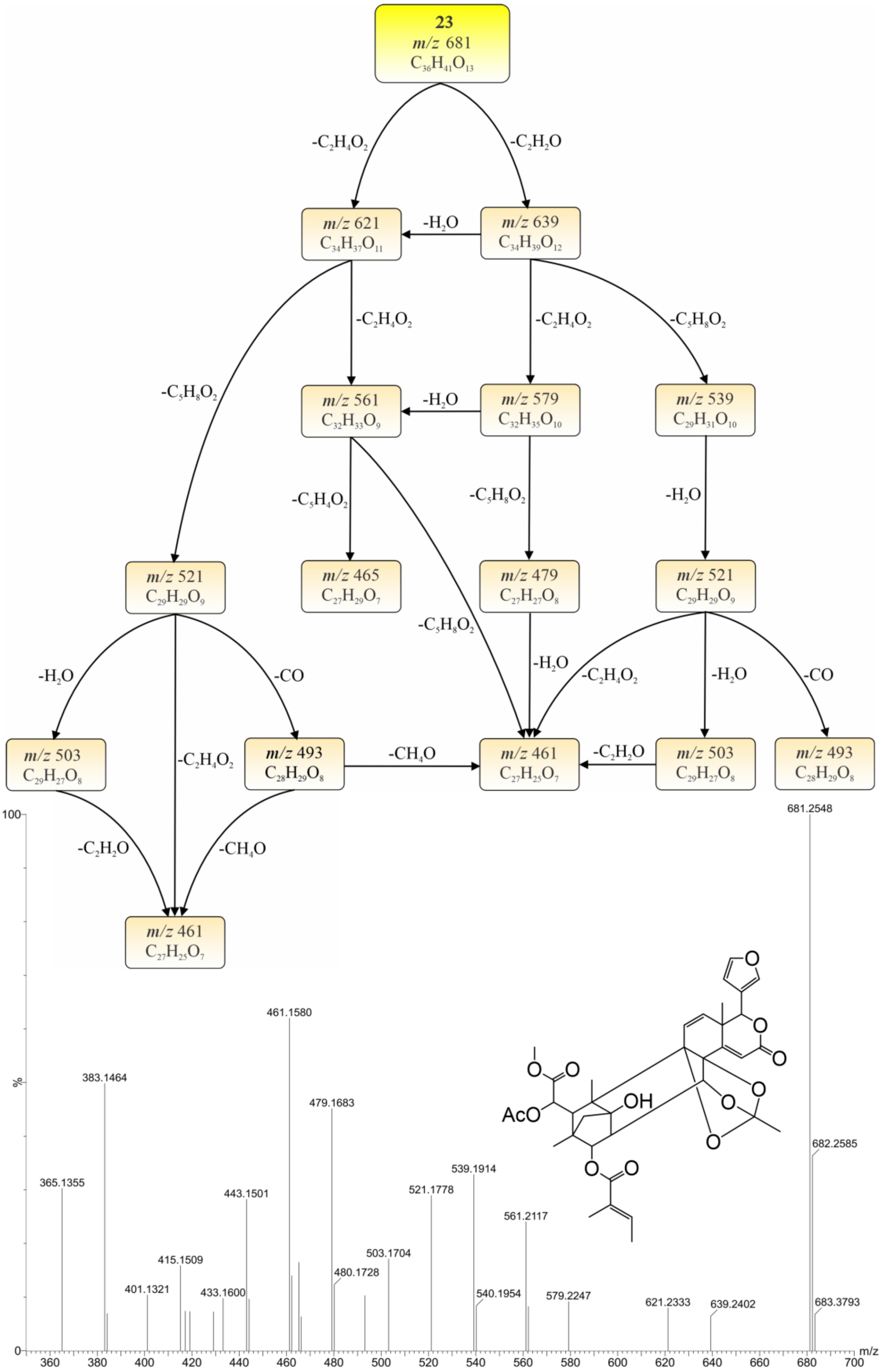

Figure S24. Fragmentation pattern of limonoid **23** computed from tandem MS data.

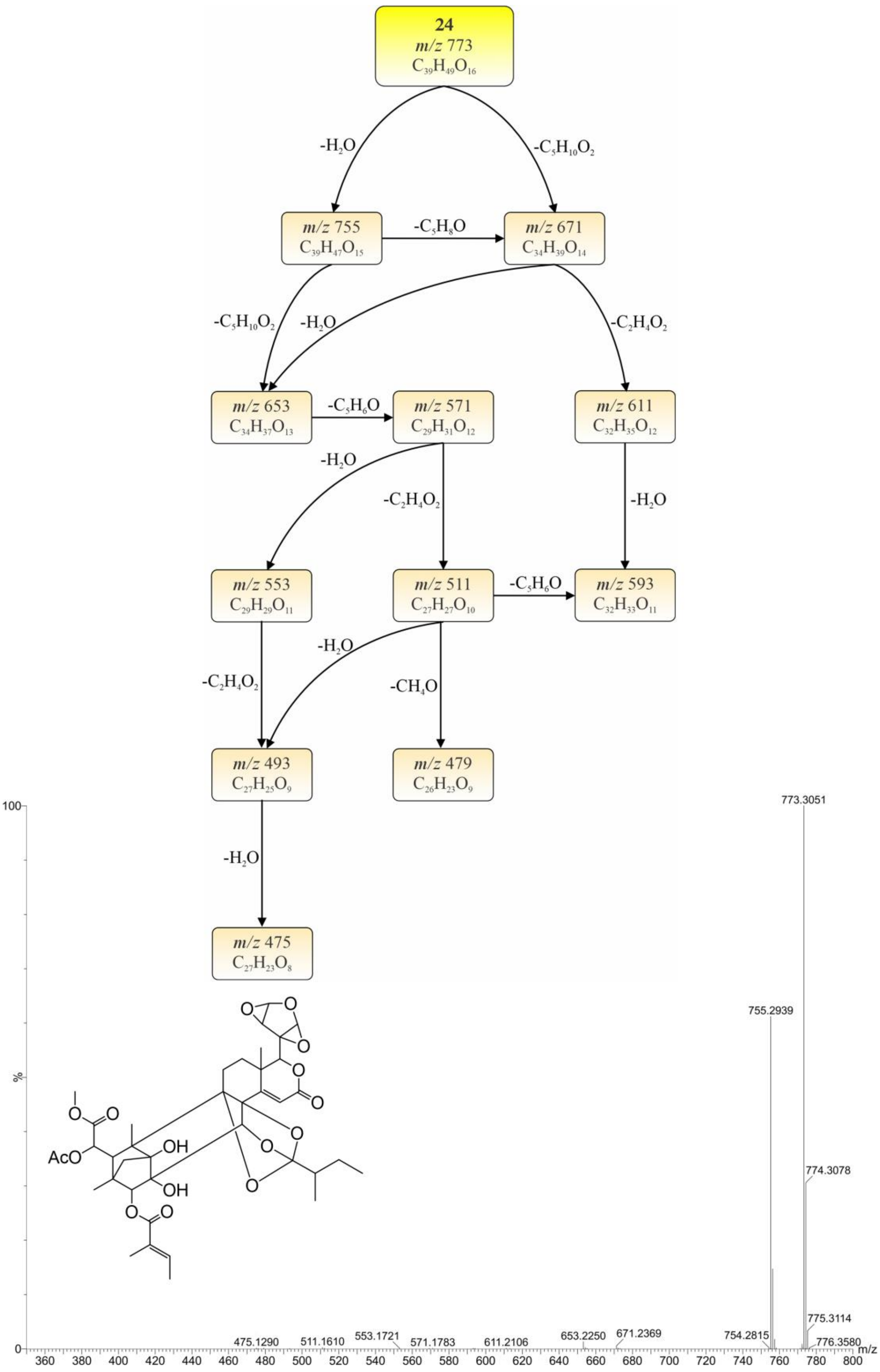

Figure S25. Fragmentation pattern of limonoid **24** computed from tandem MS data.

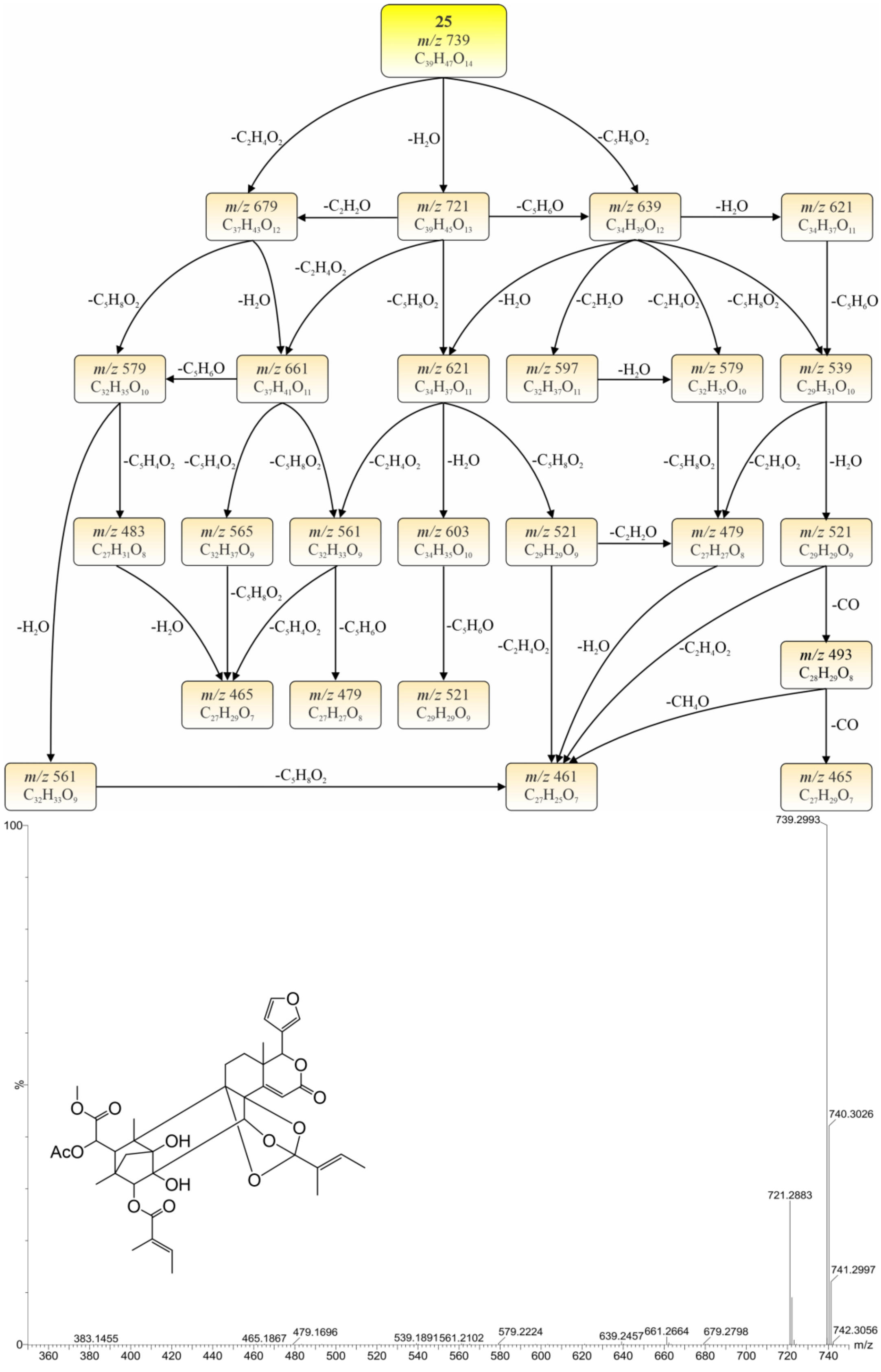

Figure S26. Fragmentation pattern of limonoid **25** computed from tandem MS data.

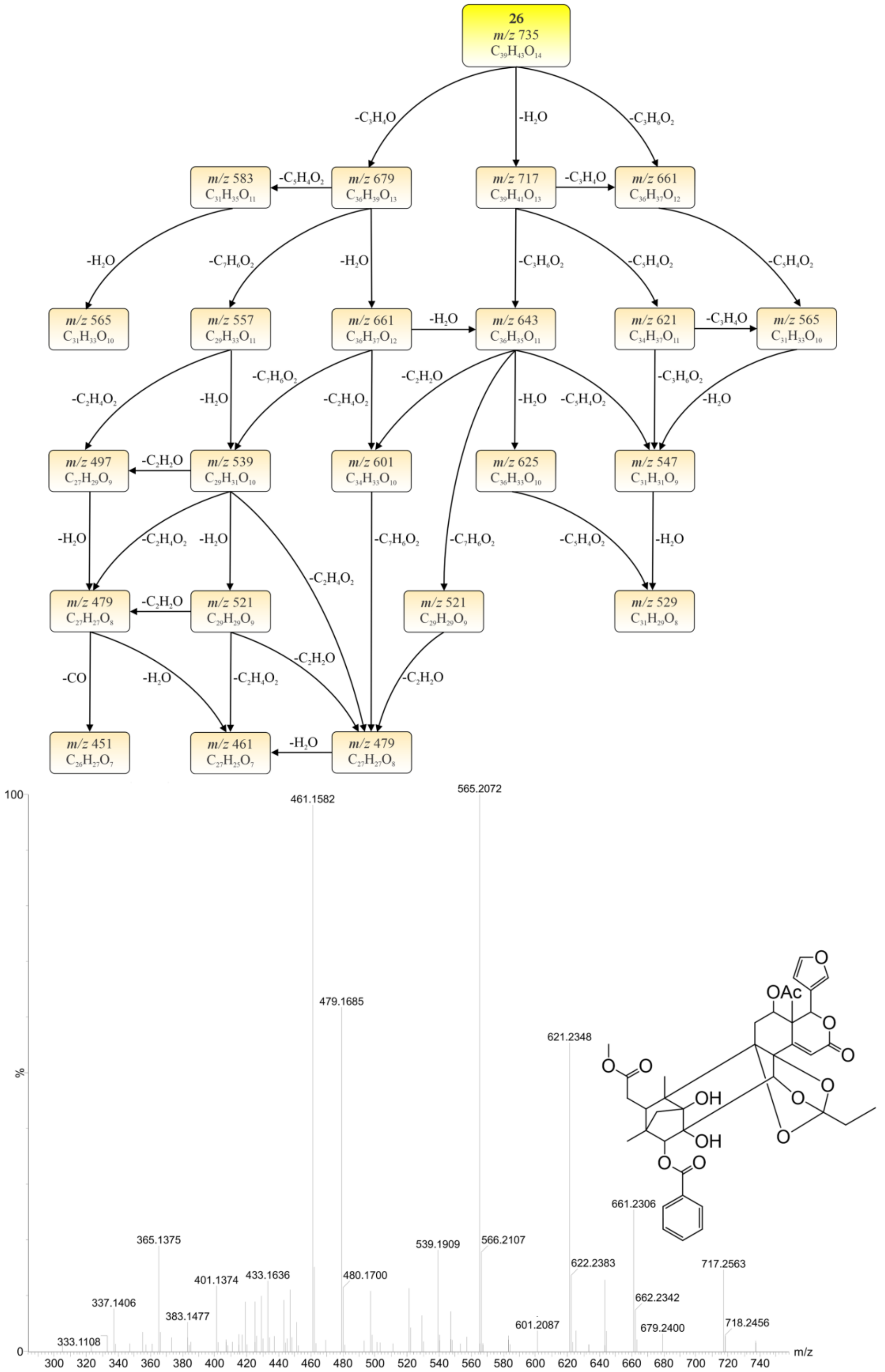

Figure S27. Fragmentation pattern of limonoid **26** computed from tandem MS data.

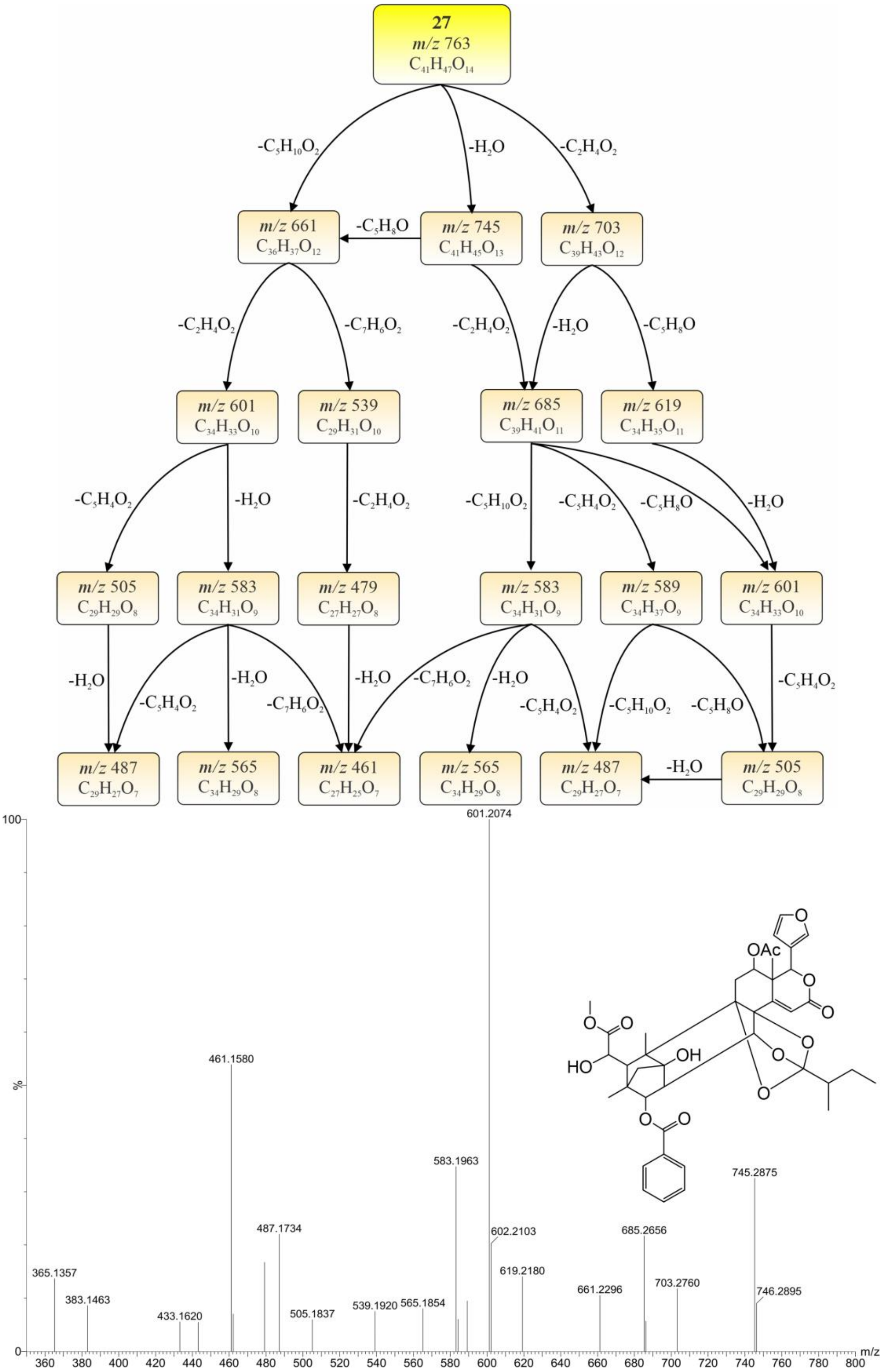

Figure S28. Fragmentation pattern of limonoid **27** computed from tandem MS data.

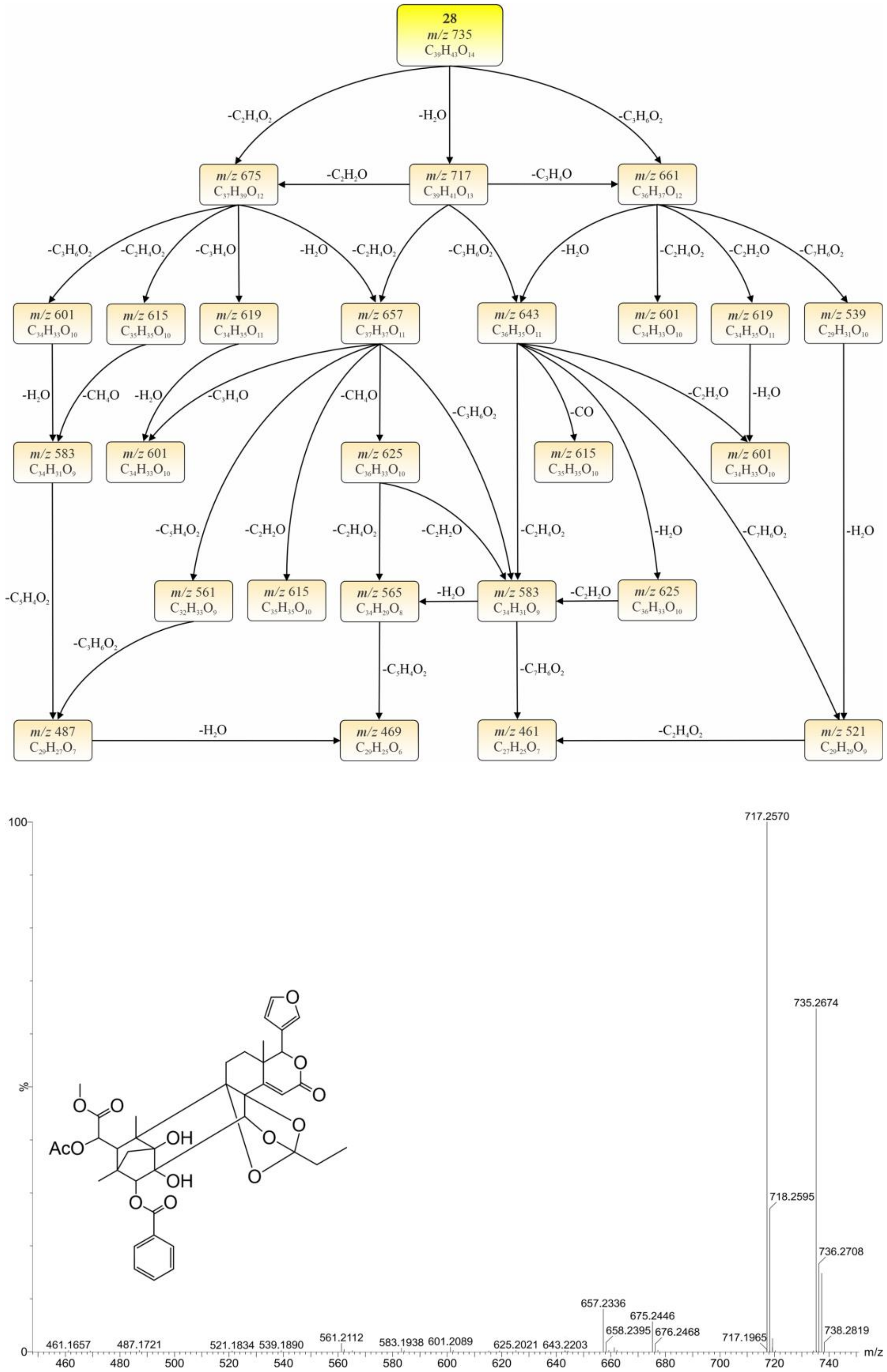

Figure S29. Fragmentation pattern of limonoid **28** computed from tandem MS data.

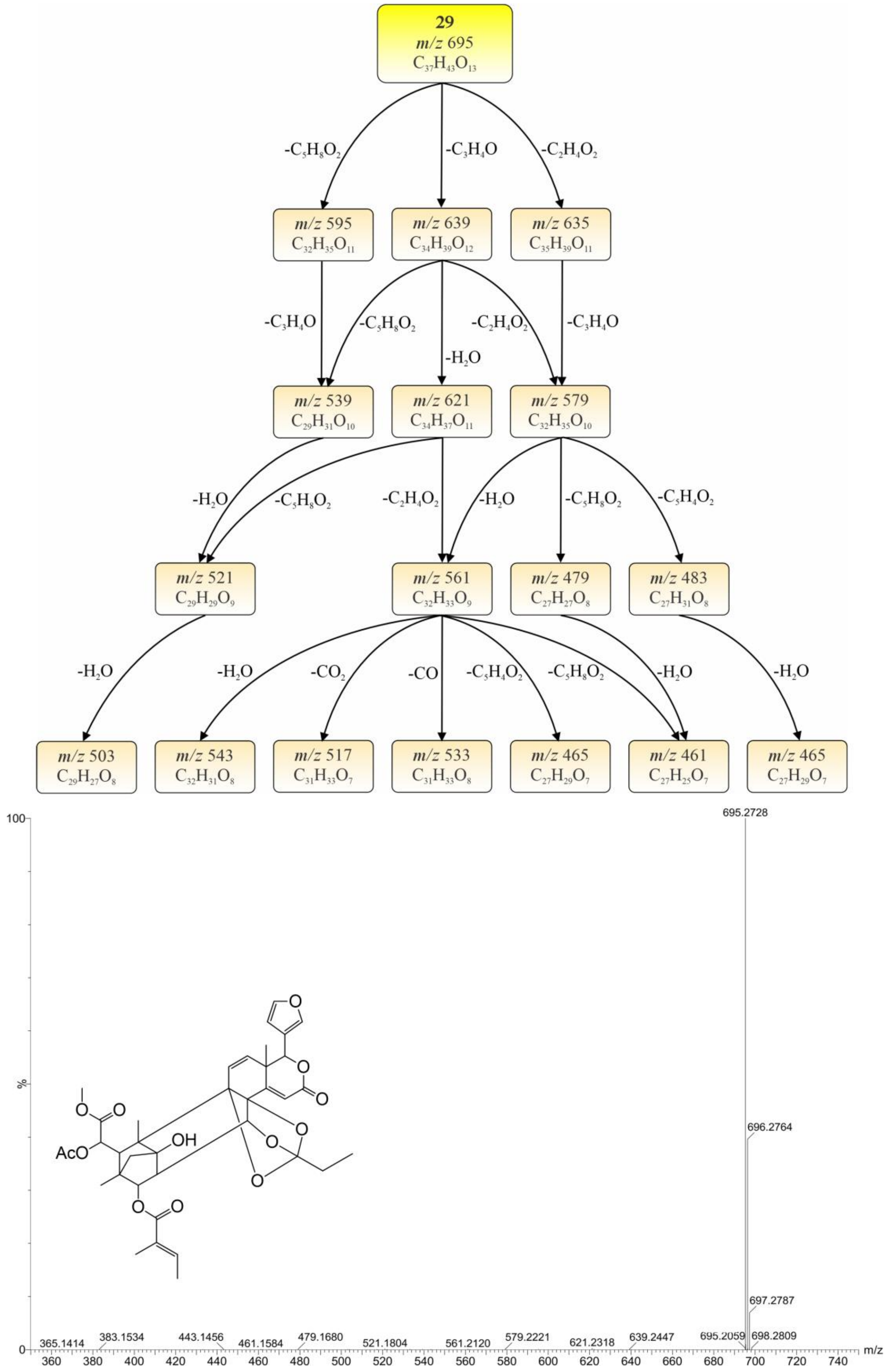

Figure S30. Fragmentation pattern of limonoid **29** computed from tandem MS data.

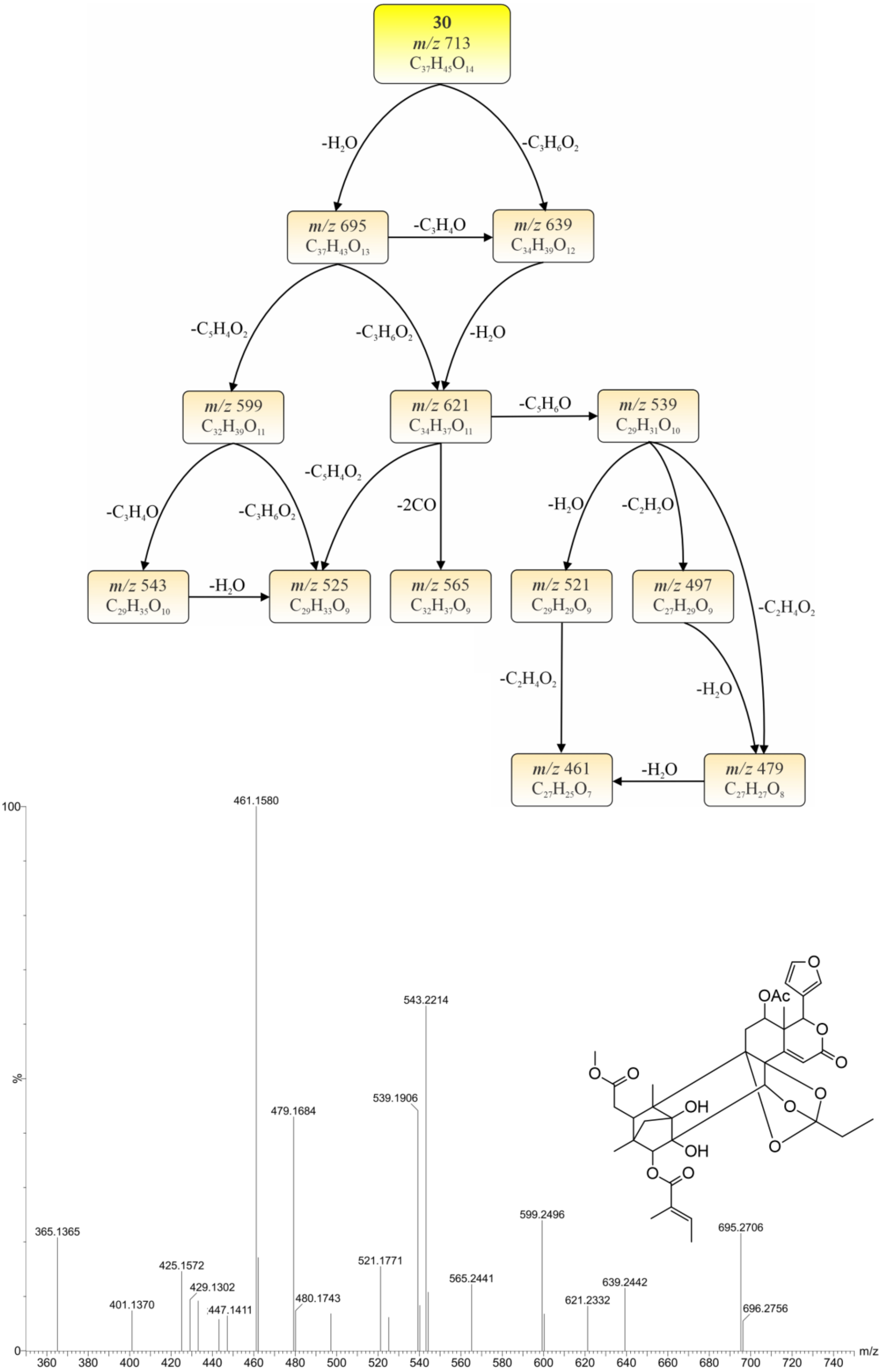

Figure S31. Fragmentation pattern of limonoid **30** computed from tandem MS data.

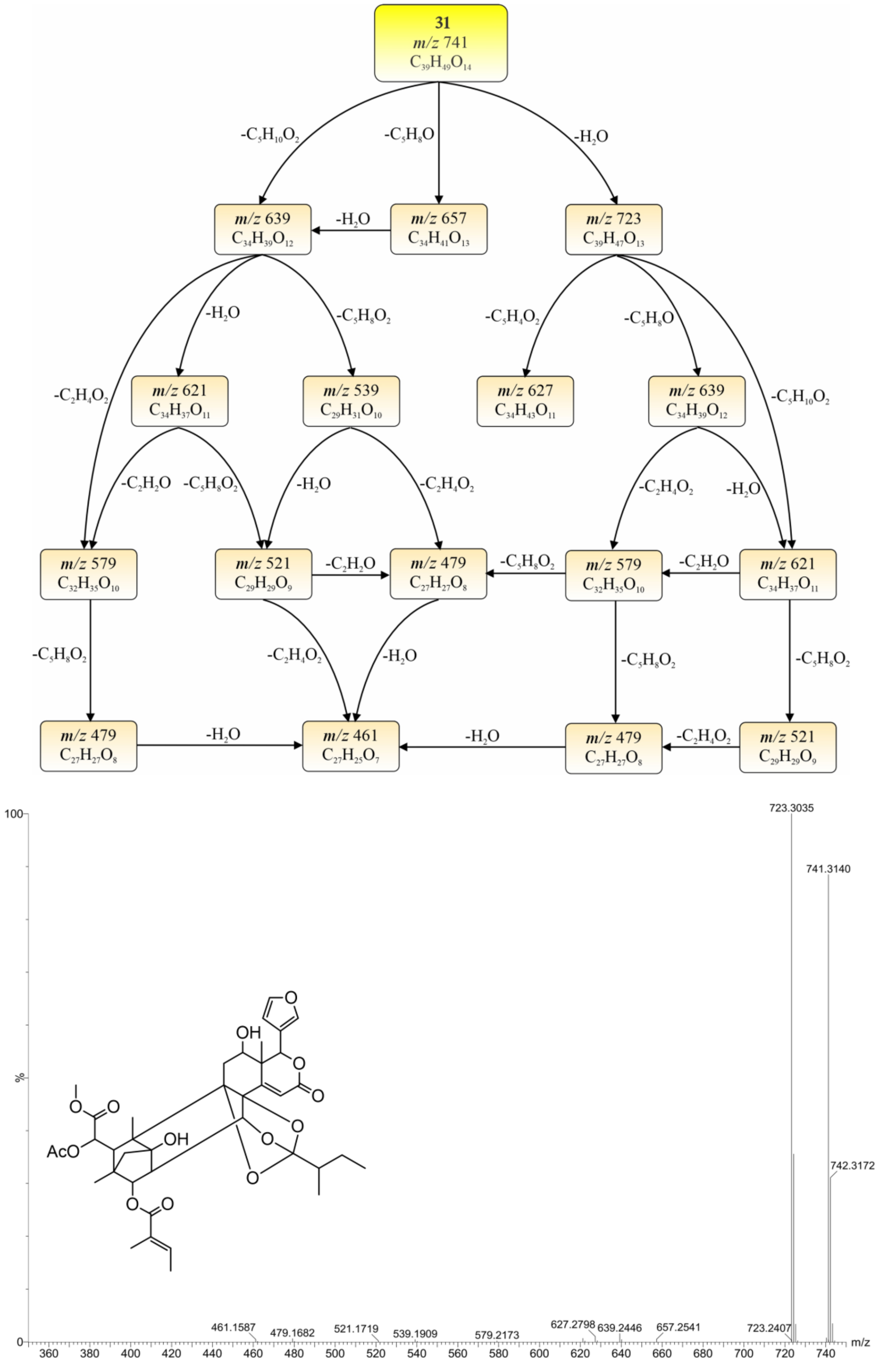

Figure S32. Fragmentation pattern of limonoid **31** computed from tandem MS data.

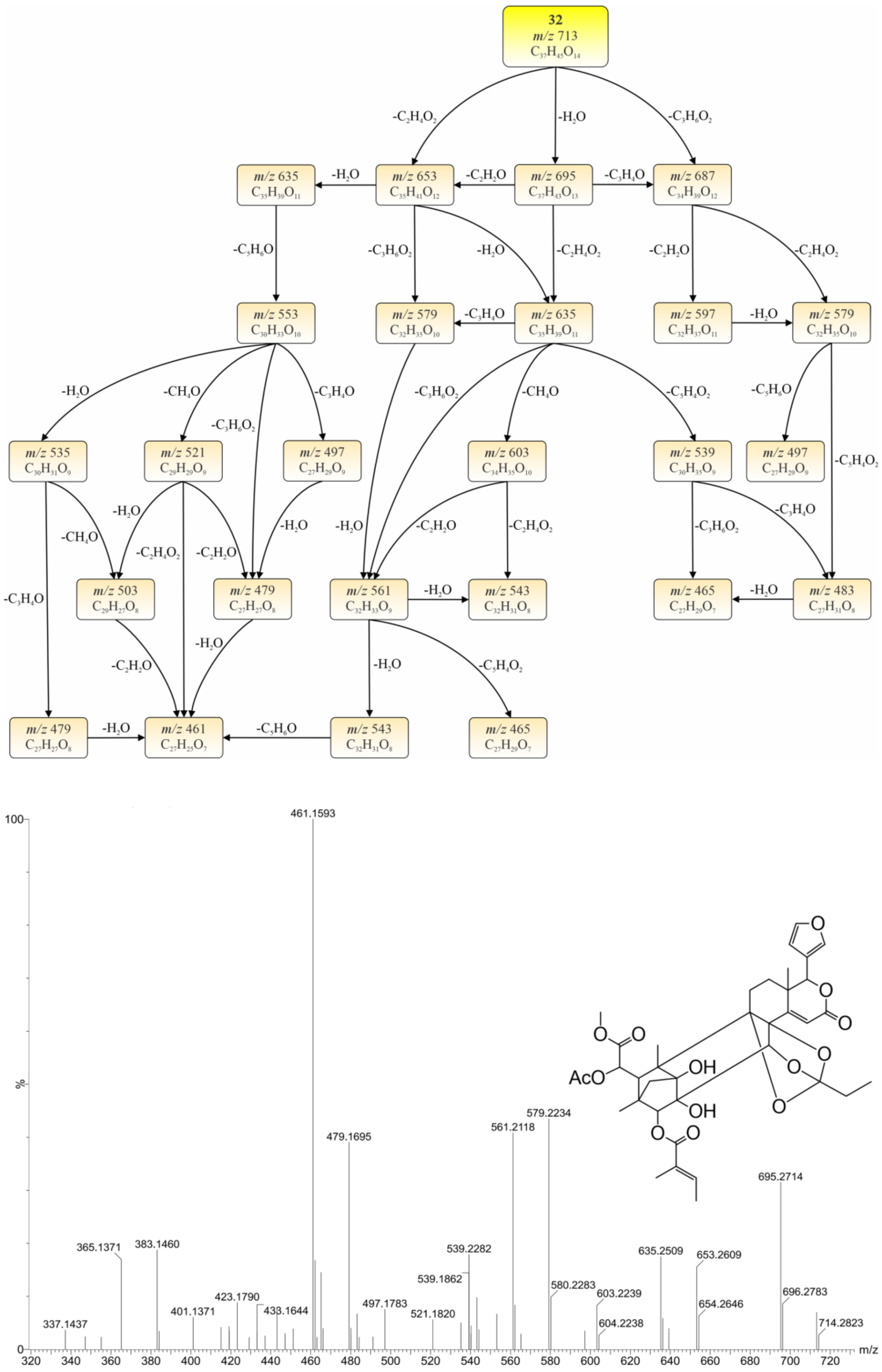

Figure S33. Fragmentation pattern of limonoid **32** computed from tandem MS data.

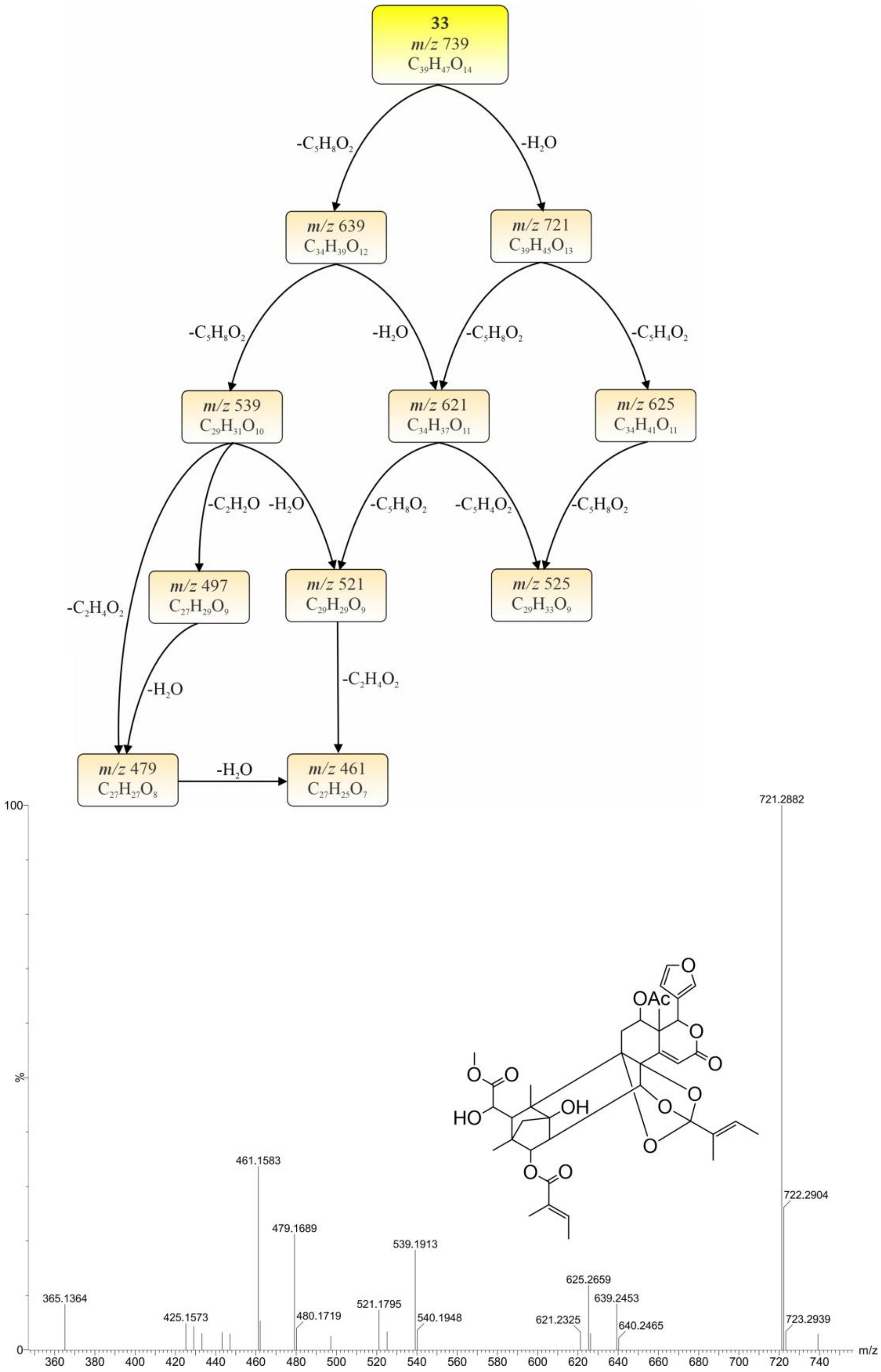

Figure S34. Fragmentation pattern of limonoid **33** computed from tandem MS data.

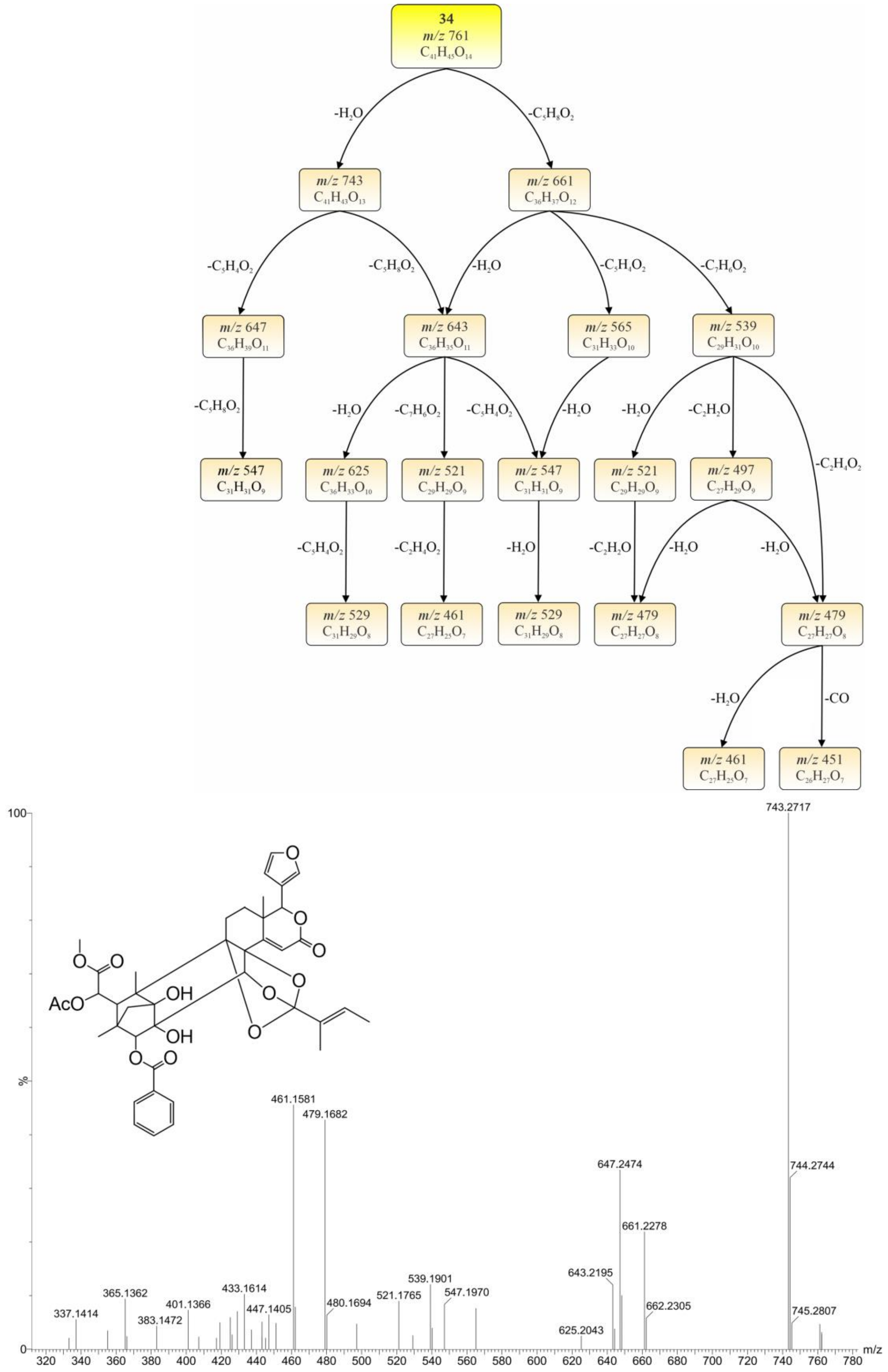

Figure S35. Fragmentation pattern of limonoid **34** computed from tandem MS data.

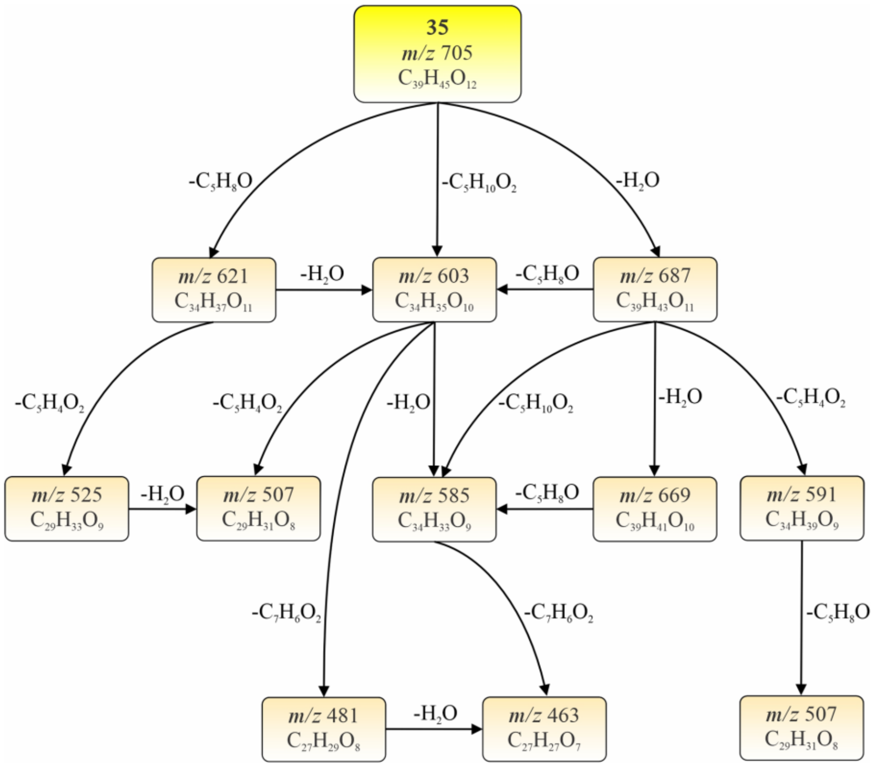

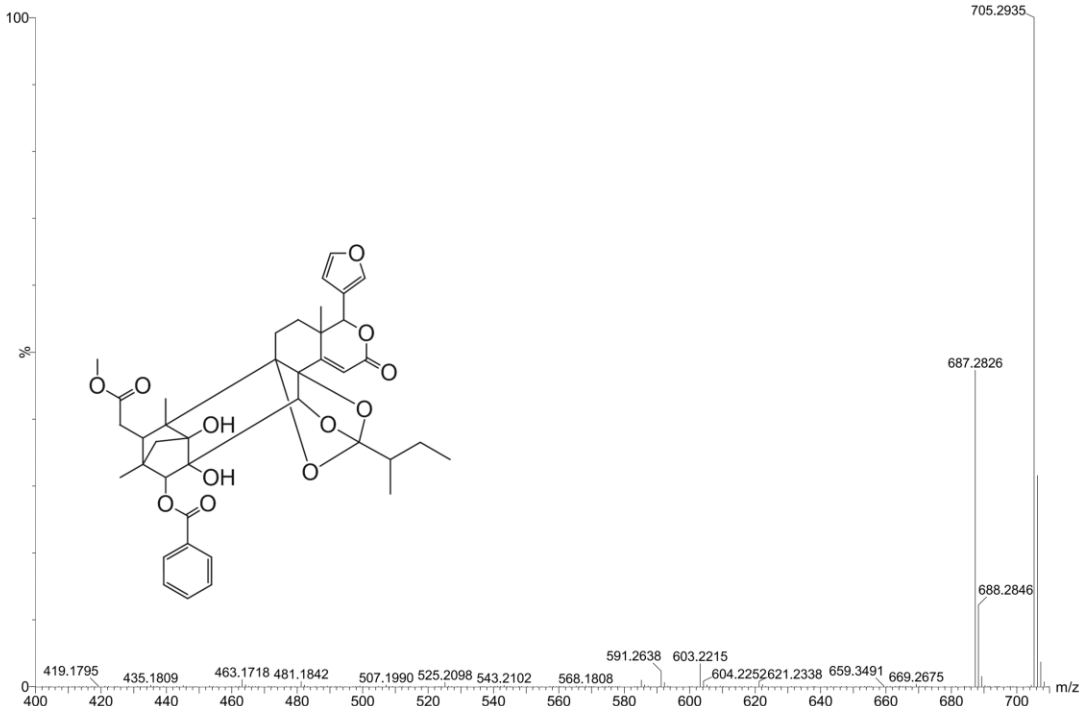

Figure S36. Fragmentation pattern of limonoid **35** computed from tandem MS data.

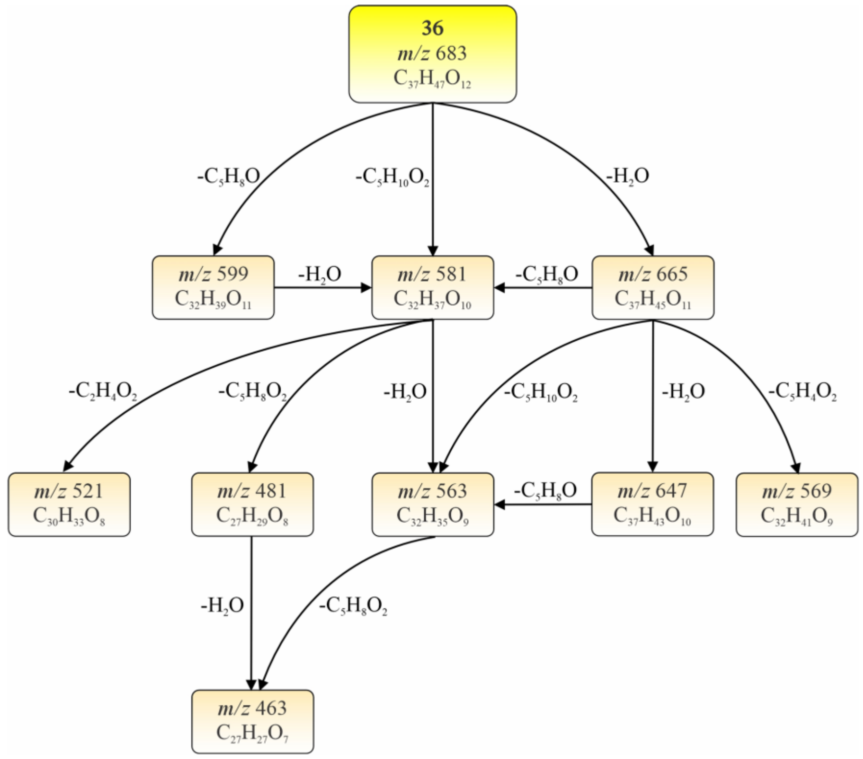

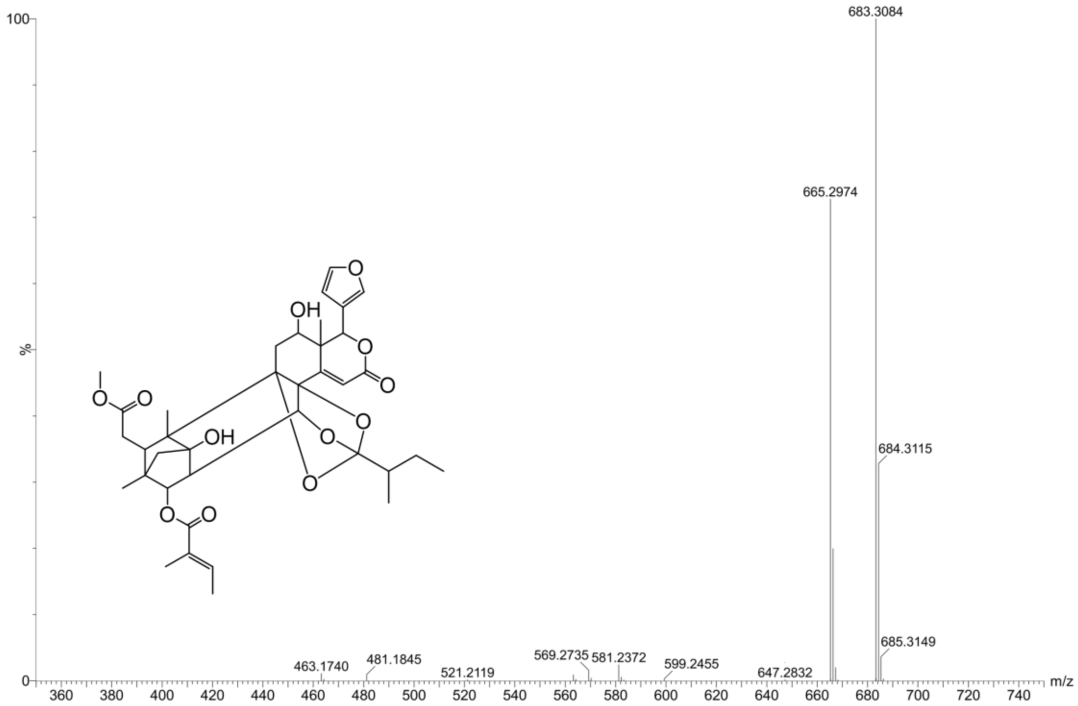

Figure S37. Fragmentation pattern of limonoid **36** computed from tandem MS data.

References

1. Abdelgaleil, S.A.M.; Doe, M.; Morimoto, Y.; Nakatani, M. Rings B,D-Seco Limonoids from the Leaves of Swietenia Mahogani. *Phytochemistry* **2006**, *67*, 452–458.
2. Pamplona, S.G.S.R.; Arruda, M.S.P.; Castro, K.C.F.; Silva, C.Y.Y. e.; Ferreira, A.G.; Silva, M.F.G.F. da; Ohashi, O.S.; Silva, M.N. da Phragmalin Limonoids from Swietenia Macrophylla and Their Antifeedant Assay against Mahogany Predator. *J. Braz. Chem. Soc.* **2018**, *29*, 1621–1629.
3. Abdelgaleil, S.A.M.; Doe, M.; Nakatani, M. Rings B,D-Seco Limonoid Antifeedants from Swietenia Mahogani. *Phytochemistry* **2013**, *96*, 312–317.
4. Shi, Z.; An, L.; Yang, X.; Xi, Y.; Zhang, C.; Shuo, Y.; Zhang, J.; Jin, D.-Q.; Ohizumi, Y.; Lee, D.; et al. Nitric Oxide Inhibitory Limonoids as Potential Anti-Neuroinflammatory Agents from Swietenia Mahagoni. *Bioorg. Chem.* **2019**, *84*, 177–185.
5. Tan, S.-K.; Osman, H.; Wong, K.-C.; Boey, P.-L. New Phragmalin-Type Limonoids from Swietenia Macrophylla King. *Food Chem.* **2009**, *115*, 1279–1285.
6. Chen, J.-J.; Huang, S.-S.; Liao, C.-H.; Wei, D.-C.; Sung, P.-J.; Wang, T.-C.; Cheng, M.-J. A New Phragmalin-Type Limonoid and Anti-Inflammatory Constituents from the Fruits of Swietenia Macrophylla. *Food Chem.* **2010**, *120*, 379–384.
7. Silva, M.N.; Arruda, M.S.P.; Castro, K.C.F.; da Silva, M.F. das G.F.; Fernandes, J.B.; Vieira, P.C. Limonoids of the Phragmalin Type from Swietenia Macrophylla and Their Chemotaxonomic Significance. *J. Nat. Prod.* **2008**, *71*, 1983–1987.
8. Mi, C.-N.; Li, W.; Chen, H.-Q.; Wang, J.; Cai, C.-H.; Li, S.-P.; Mei, W.-L.; Dai, H.-F. Two New Compounds from the Roots of Swietenia Macrophylla. *J. Asian Nat. Prod. Res.* **2019**, *21*, 1005–1012.
9. Zhang, J.; Li, W.; Dai, Y.; Shen, L.; Wu, J. Twenty-Nine New Limonoids with Skeletal Diversity from the Mangrove Plant, Xylocarpus Moluccensis. *Mar. Drugs* **2018**, *16*, doi:10.3390/md16010038.
